# Kyungok-go for fatigue in patients with long COVID: Double-blind, randomized, multicenter, pilot clinical study protocol

**DOI:** 10.1371/journal.pone.0319459

**Published:** 2025-04-01

**Authors:** Jiwon Yoon, Sanghyun Kim, Chan-Young Kwon, Jung Won Kang, Tae-Hun Kim, Sunoh Kwon

**Affiliations:** 1 Korean Medicine Data Division, Korea Institute of Oriental Medicine, Daejeon, Republic of Korea; 2 Department of Medical Classics, College of Korean Medicine, Daejeon University, Daejeon, Republic of Korea; 3 Department of Oriental Neuropsychiatry, Dong-eui University College of Korean Medicine, Busan, Republic of Korea; 4 Department of Acupuncture & Moxibustion, College of Korean Medicine, Kyung Hee University, Seoul, Republic of Korea; 5 Korean Medicine Clinical Trial Center, Korean Medicine Hospital, Kyung Hee University, Seoul, Republic of Korea; 6 Korean Medicine Convergence Research Division, Korea Institute of Oriental Medicine, Daejeon, Republic of Korea; Johns Hopkins University, UNITED STATES OF AMERICA

## Abstract

The most common symptom reported by patients after recovery from coronavirus disease 2019 (COVID-19) is fatigue. However, robust clinical evidence supporting the effectiveness of treatments and interventions for fatigue in COVID-19 survivors is lacking. This pilot clinical trial aims to assess the safety and efficacy of Kyungok-go, a herbal preparation targeting fatigue, in patients after recovering from COVID-19. The study will include 100 participants with persistent fatigue for more than 12 weeks after COVID-19 recovery. They will be randomly allocated into two groups: the Kyungok-go group (n =  50) and the placebo group (n =  50). Kyungok-go or placebo will be administered twice daily for 12 weeks, and the participants will be assessed at 4-week intervals. The primary outcome will be the change in the Fatigue Severity Scale score. Secondary outcomes will include cognitive function, physical function, quality of life, depression, sleep quality, medication adherence, and feasibility. This study is the first attempt to investigate the safety and efficacy of Kyungok-go for relieving fatigue related to long COVID. The results are expected to contribute to the establishment of a knowledge base and reveal the potential of herbal medicine prescriptions for managing and recovering from the most common sequelae of COVID-19.

**Trial registration number:** KCT0008789.

## Introduction

The coronavirus disease 2019 (COVID-19) pandemic was a worldwide phenomenon. As of 13 October, 2024, the World Health Organization had recorded over 776 million confirmed cases of COVID-19 and over 7 million associated deaths [1]. Post-COVID syndrome, also known as long COVID, has been widely reported after COVID-19. According to a recent meta-analysis, approximately 80% of patients experienced at least one symptom that lasted from 2 weeks to 4 months after severe acute respiratory syndrome coronavirus 2 (SARS-CoV-2) infection during the follow-up period [2].

The temporal definition of long COVID, commonly known as post-acute sequelae of SARS-CoV-2 infection, is not clearly established and varies among countries and institutions. The World Health Organization defines post-COVID conditions (i.e., long COVID) as symptoms occurring within 3 months after SARS-CoV-2 infection and persisting for at least 2 months without an alternative diagnosis [3]. The US Centers for Disease Control and Prevention defines post-COVID conditions as symptoms that extend beyond 4 weeks of SARS-CoV-2 infection [4], and the National Institute for Health and Care Excellence defines post-COVID syndrome as symptoms that last for 4–12 weeks after COVID-19 diagnosis and persist beyond 12 weeks without an alternative explanation [5]. In South Korea, long COVID is defined as the persistence of one or more symptoms/signs, such as fatigue, shortness of breath, depression, anxiety, and cognitive decline, after 12 weeks of COVID-19 diagnosis that cannot be explained by other diseases [6].

Fatigue is the most common nonrespiratory manifestation of long COVID and was reported by 28.4% and 34.8% of 735,006 hospitalized patients and outpatients, respectively [7]. Temporally, fatigue was reported in 32%, 36%, 47%, and 41% of COVID-19 patients during follow-up periods of 3– < 6 months, 6– < 9 months, 9– < 12 months, and > 12 months, respectively [8]. Chronic fatigue following viral infection may be due to a complex mechanism related to inflammatory response pathways [9], as well as various central, peripheral, and psychological factors. Thus, a comprehensive approach that restores immune function and improves organ function will be necessary to address the various sequelae observed in long COVID [10]. However, there is a lack of high-level evidence to support the treatment and interventions for managing fatigue in long COVID, and therapy mainly focuses on symptomatic treatment and lifestyle management [11]. Therefore, research in this area is critical.

A survey of Korean medicine doctors reported that herbal medicine was the most effective and widely used treatment for patients with chronic fatigue syndrome and idiopathic chronic fatigue [12]. Among herbal prescriptions, Kyungok-go is frequently prescribed for fatigue recovery [13] and is clinically used to improve postviral fatigue and general symptoms. A study on COVID-19 patients who received Korean medicine treatment during the first half of 2020 reported that the most frequently prescribed herbal medicine was Kyungok-go and that herbal medicine treatment led to the improvement of all COVID-19-related symptoms, including fatigue [14]. Because Kyungok-go is listed by the Korean Ministry of Food and Drug Safety as a herbal medicine with indications for middle-aged and post-illness fatigue, weak constitution, and physical fatigue [15], it is considered suitable for alleviating fatigue associated with long COVID. However, robust clinical evidence is lacking, which is essential for developing a Korean medicine approach to managing long COVID.

## Methods and analysis

### Objectives

This research is a phase II preliminary randomized clinical trial aimed at evaluating the efficacy and safety of Kyungok-go for treating fatigue in patients with long COVID. The secondary aim of this study is to assess the feasibility of the selected study design and explore the potential therapeutic mechanisms of Kyungok-go.

### Study design

**Fig 1:**
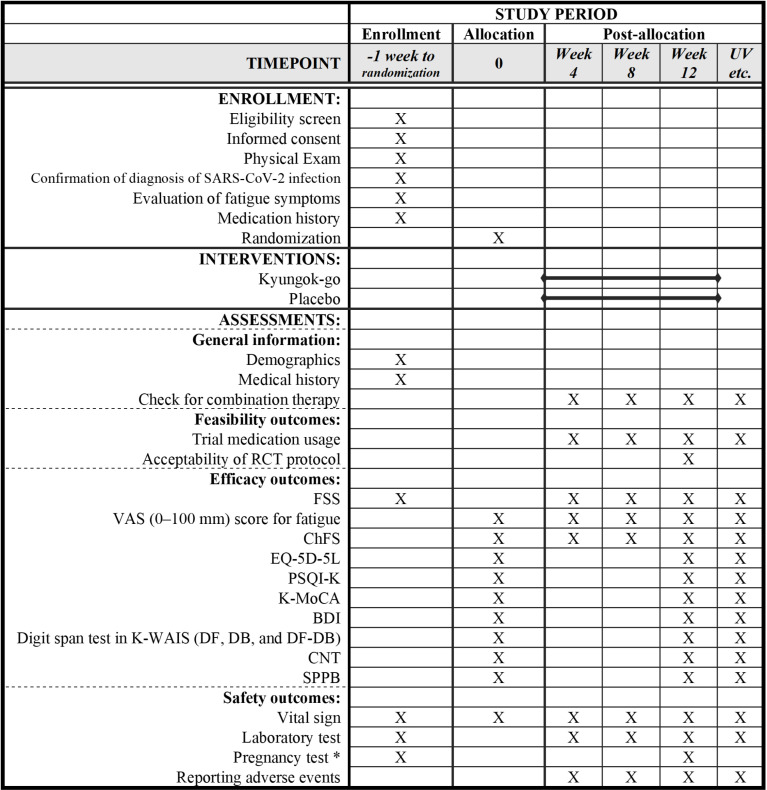
SPIRIT schedule of enrollment, interventions, and assessments. Abbreviations. BDI, Beck’s Depression Inventory; ChFS, Chalder Fatigue Scale; CNT, Computerized neurocognitive function test; DB, digits backward; DF, digits forward; FSS, Fatigue Severity Scale; K-MoCA, Korean-Montreal Cognitive Assessment; K-WAIS, Korean-Wechsler Adult Intelligence Scale; PSQI-K, Korean version of Pittsburgh Sleep Quality Index; SARS-CoV-2, severe acute respiratory syndrome coronavirus 2; SPPB, Short Physical Performance Battery; UV, unscheduled visit; VAS, visual analogue scale.  * Only for women of reproductive potential.

**Fig 2:**
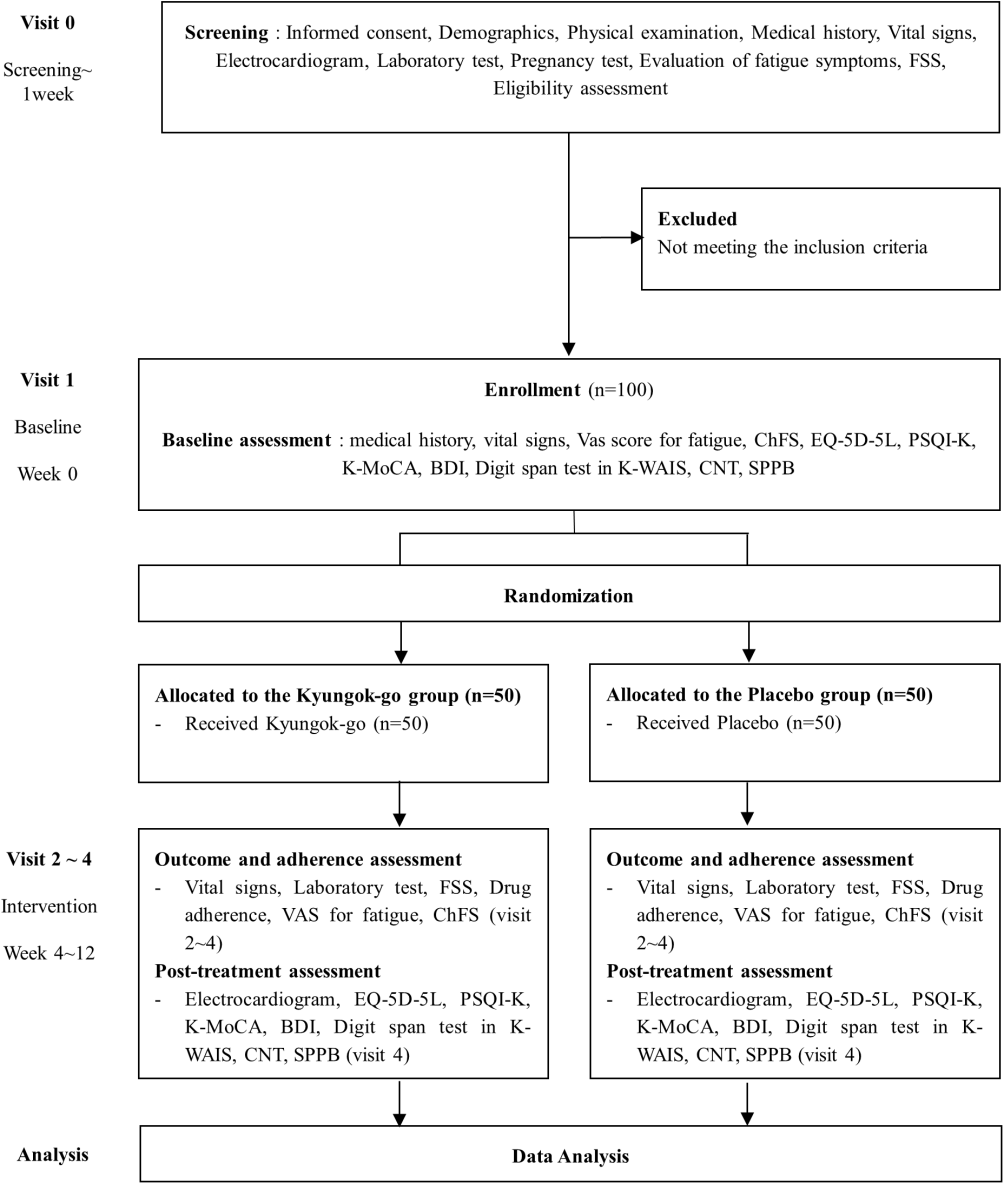
Flow chart of the trial procedure.

Research participants will listen to a sufficiently detailed explanation of this clinical trial, sign the consent form, and undergo a screening process to determine if they meet the study criteria. Then, eligible participants will be randomly assigned to either the treatment group (Kyungok-go administration group) or the control group (placebo group) and will receive the prescribed intervention for 12 weeks. Each subject will undergo monthly follow-ups during the 12-week intervention administration period and then undergo evaluation of symptoms at the end of the 12 weeks. This protocol is in accordance with the Standard Protocol Items: Recommendations for Interventional Trials guidelines [16]. The SPIRIT schedule for this study can be found in Fig 1. The flowchart is presented in Fig 2.

### Sample size

Given the preliminary nature of this clinical study, no formal sample size calculations were performed. However, determining the optimal sample size for such studies remains a nuanced endeavor. Depending on the research aims and design complexities, sample sizes can vary considerably, ranging from as few as 10-12 participants per group to as many as 60-75 participants per group [17]. For a high level of confidence, a sample size of at least 50 per group is recommended for pilot Randomized Controlled Trial (RCT) [18]. A hypothetically calculation the sample size based on the Checklist Individual Strength (CIS) results from our previous clinical study [19], and conservatively adopting the Minimal Clinically Important Difference (MCID) of 10 points [20], 196 participants would be necessary for assessing clinical efficacy. This calculation considers an alpha level of 0.05, a beta of 20%, a dropout rate of 10%, and a standard deviation (SD) of 23.712 [19]. In our study, we have decided to proceed with recruiting half of this number of participants considering that it is a pilot study. Feasibility studies play a crucial role in establishing protocols and refining methodologies for future high-quality RCTs. Therefore, one hundred participants will be recruited, of which 50 will be allocated to the treatment group and 50 to the control group.


$$n=\frac{2{\left(Z_{a}/2+Z_{\text{β}}\right)}^{2}\sigma^{2}}{{\left(\mu_{c}-\mu_{t}\right)}^{2}}.$$

### Participants

#### Inclusion criteria.

The inclusion criteria are as follows:

- Individuals aged > 19 years who have been diagnosed with COVID-19 for a minimum duration of 12 weeks or exhibit persistent fatigue that was not present before COVID-19 diagnosis for > 4 weeks.
- Individuals scoring > 4 points on the Fatigue Severity Scale (FSS).
- Individuals experiencing fatigue not attributable to another medical condition.
- Participants demonstrating no overall cognitive function issues who willingly provide written consent for study participation.

#### Exclusion criteria.

- Individuals with diagnosed diseases that may lead to fatigue, such as cancer, insomnia, chronic hepatitis, liver cirrhosis, chronic renal failure, active tuberculosis, latent tuberculosis, asthma, multiple sclerosis, and other similar conditions, but not limited to those specifically mentioned.
- Individuals experiencing medical issues affecting drug intake or absorption (dysphagia, clinically significant digestive disorders, galactose intolerance, Lapp lactase deficiency, or glucose–galactose malabsorption).
- Individuals with uncontrolled diabetes (HbA1c ≥  6.5%).
- Individuals with uncontrolled hypertension (systolic blood pressure ≥  160 mmHg or diastolic blood pressure ≥  100 mmHg).
- Individuals with current or past allergies to the investigational drug (Kyungok-go).
- Individuals diagnosed with liver or kidney disease or exhibiting abnormal liver and kidney function blood test results (AST, ALT, BUN, or creatinine exceeding twice the upper limit of normal).
- Pregnant, potentially pregnant, or lactating women.
- Individuals who do not consent to using contraception.
- Individuals who participated in another clinical study within 30 days before joining this study.
- Individuals experiencing symptoms of appetite loss, aversion, or vomiting.
- Individuals who consumed herbal or traditional medicines, over-the-counter drugs, nonprescription medications, vitamin supplements, health functional foods, or any substances that could affect fatigue within the month prior to the clinical trial.
- Individuals who have taken medications that can affect fatigue within 30 days before participating in the clinical trial (corticosteroids within 1 month of the final dose, immunosuppressants within 3 months of the final dose, antipsychotic drugs within 1 month of the final dose, hormone preparations within 1 month of the final dose).
- Individuals who have undergone organ or bone marrow transplantation.
- Individuals who have undergone procedures (such as surgery) within the past 3 months that may impact fatigue.
- High-risk alcohol consumers (>7 drinks per occasion for men or > 5 drinks per occasion for women, where one drink is equivalent to 4 cans of beer, 4 bowls of makgeolli, 4 glasses of wine, 1 bottle of soju, or 4 glasses of whiskey for men and three cans of beer, three bowls of makgeolli, three glasses of wine, 3/4 bottle of soju, or 3 glasses of whiskey for women).
- Long-term smokers (>1 pack daily for > 10 years).
- Individuals with clinically significant psychiatric symptoms, medical conditions, or laboratory findings that will make study participation challenging, as judged by the researcher.

#### Withdrawal and discontinuation criteria.

- Severe acute reaction (allergy, hypersensitivity, and others) to the intervention.
- Confirmation of a major violation of the inclusion/exclusion criteria during the trial.
- Inability to take the intervention or make observations due to unexpected diseases or accidents.
- Inability to take the intervention or make observations due to severe adverse events or adverse drug reactions.
- Pregnancy diagnosis.
- Withdrawal of informed consent by the participant or their representative.
- Significant deviations from the clinical trial protocol.
- Emergence of new conditions during the clinical trial period that may impact fatigue (e.g., SARS-CoV-2 reinfection).
- Trial continuation deemed inappropriate by the researchers.

### Recruitment

Recruitment of the participants is planned to start after obtaining approval of investigational new drug application (IND) from the Korean Ministry of Food and Drug Safety, with an expected start date of January 2025. All participants will be recruited, and end of treatment is expected to be by January 2027.

The total number of study participants will be 100, with 50 individuals each assigned to the treatment and the control groups, respectively. Table 1 presents the recruitment distribution across the participating institutions.

**Table 1:** The recruitment distribution across participating institutions.

Institution	Treatment Group (Kyungok-go group)	Control Group (Placebo group)	Total
Kyung Hee University Korean Medicine Hospital	30	30	60
Dong-eui University Korean Medicine Hospital	20	20	40
Total	50	50	100

### Blinding and treatment allocation

The researcher assigns a unique screening number to each subject at Visit 0. This number starts with “CVS1-01” for Kyung Hee University Korean Medicine Hospital and “CVS2-01” for Dong-eui University Korean Medicine Hospital, and is composed of the smallest available number among the assigned serial numbers. Once assigned, a subject number cannot be reused. If the subject is selected as a participant through the screening process, a randomization number is assigned. This number starts with “CVR1-01” for Kyung Hee University Korean Medicine Hospital and “CVR2-01” for Dong-eui University Korean Medicine Hospital and is also composed of the smallest available number among the assigned serial numbers. If, for any reason, treatment is not initiated for the subject at Visit 1 (V1), the assigned subject number and the reason for not initiating treatment must be documented in the screening log and the case report form (CRF).

Block randomization will be implemented, and the block size will remain undisclosed to maintain blinding. An independent statistician will generate random numbers using STATA Version 4.2 (StataCorp LLC., Texas). The generated random numbers will be sent to the labeling department for investigational drugs, where both the investigational product and the control product will be packaged according to the randomization sequence, and the randomization numbers will be labeled on the packaging. This process ensures double blinding for both researchers and participants. The allocation ratio between the treatment and control groups will be 1:1.

To maintain allocation concealment, investigational drugs packaged according to the random numbers generated by the independent statistician will be distributed to the clinical trial sites. Participants who receive sufficient information about the trial, meet the inclusion criteria, do not fall under the exclusion criteria, and voluntarily provide written consent will be enrolled. Investigators at each site will assign randomization numbers sequentially, starting from the smallest available number, to participants based on their order of enrollment. These numbers will be recorded in the CRF, and the investigational drugs will then be dispensed to the participants.

### Intervention

Participants will take one sachet (22.5 g) of Kyungok-go or placebo twice daily before meals or during intervals between meals (between breakfast and dinner). The placebo will be formulated as comparable to Kyungok-go in terms of appearance, taste, color, smell, texture, and weight. Table 2 summarizes the preparations used in this study.

**Table 2:** Summary table of the preparations used by the study.

Preparations	Formulation	Source	Species, concentration	Quality Control reported? (Y/N)	Chemical Analysis reported? (Y/N)
Kyungok-go	Soft extract with dark brown color, viscosity in aluminum stick packaging	KYUNGJIN PHARM CO., Ltd. (Icheon-si, Gyeonggi-do, Korea)	Powder of *Panax ginseng* (6.2 g) Sclerotium of *Poria cocos* (12.4 g) Juice of *Rehmannia glutinosa* (39.9 g) Honey (41.5g)	Y-Prepared in a Good Manufacturing Practice based environment	Y
Placebo			Purified water (65 g) Sodium benzoate (0.06 g) Citric acid hydrate (0.09 g) Sodium citrate monohydrate (0.04g) High-fructose corn syrup-55 (21g) White sugar (14 g) Caramel (5g) Xanthan gum (3.8g) concentrated glycerin (3.5g) ginseng flavor A-980306 (0.01 g)		

### Outcomes

#### Primary outcome.

The primary outcome will involve evaluating and comparing FSS scores between the two groups following intervention administration over a period of up to 12 weeks. The FSS questionnaire, which assesses fatigue in the past week, will be directly administered to the subject under the direction of the research director or person in charge [21]. It consists of nine questions. Each question is evaluated on a scale of 1–7, and the more severe the symptoms, the higher the score. The FSS score is then calculated as the average of the scores of nine items. Based on the treatment guidelines for long COVID and clinical research results, a score greater than 4 points indicates that the person has significant fatigue symptoms [22]. Fatigue during the past week before (Visit 1) and after herbal medicine administration (Visit 4) will be assessed, and comparative analyses will be conducted to compare the two study groups.

#### Secondary outcomes.

The selection of secondary outcome measures was based on the complex and multifaceted nature of fatigue in long COVID [23]. Post-COVID fatigue is closely interconnected with various physical, cognitive, and psychological manifestations that can affect and exacerbate fatigue symptoms [24]. Specifically, the inclusion of cognitive function assessments is justified by the established relationship between cognitive dysfunction and fatigue in long COVID. Also, research has demonstrated that depression and sleep problems are not only common comorbidities but can also amplify fatigue symptoms in long COVID patients [25]. These comprehensive secondary outcome measures will not only help validate the primary findings but also provide valuable insights into the various mechanisms through which Kyungok-go may affect fatigue and related symptoms in long COVID patients.

#### Medication adherence.

Medication adherence will be meticulously tracked by assessing the number of returned and taken medications during each visit. In instances where the count of returned and taken drugs deviates from the number of prescribed drugs, the reasons for this disparity will be methodically documented in the case report form. The medication adherence rate will be calculated as a percentage using the following formula:


$$Medicationadherence\left(\%\right)=\frac{\left(Numberoftakenmedications\right)}{\left(Numberofprescribedmedicationsintheduration*\right)}\times 100$$

*The number of prescribed medications in the duration refers to the medication intended for consumption from the time of initial administration until the return of any untaken drugs.

Medication adherence (%) will be calculated and documented at Visits 2, 3, and 4 (or the endpoint of medication). Descriptive statistics, including the mean, standard deviation, median, and minimum and maximum values of the final medication adherence assessment (Visit 4 or the endpoint of medication), will be presented for each intervention group and then analyzed and compared to identify any significant differences between the two groups.

#### Fatigue severity scale.

FSS scores will be compared between the two groups at Visits 2 and 3.

#### Chalder fatigue scale.

The Korean version of the Chalder Fatigue Scale questionnaire [26] will be administered at Visits 1, 2, 3, and 4, and the results will be recorded. While FSS focuses on the severity and impact of fatigue on daily activities, ChFS provides detailed insights into the physical (questions 1-7) and mental (questions 8-11) components of fatigue, which are particularly relevant for long COVID patients who often report both physical exhaustion and cognitive fatigue [23]. Each item is evaluated on a scale of 0–4, with 0 indicating no symptoms and 4 indicating the maximum level of symptoms. The total score ranges from 0 to 33, reflecting both physical and psychological fatigue.

#### EQ-5D-5L.

The EQ-5D-5L, a standardized measurement of health-related quality of life, measures the quality of life across five dimensions: mobility, self-care, usual activity, pain/discomfort, and anxiety/depression [27]. Each dimension has five levels: 1, no problems; 2, slight problems; 3, moderate problems; 4, severe problems; and 5, unable to (for mobility, self-care, and usual activities), extreme (pain/depression), or extremely (anxious/depressed). The EQ-D-5L will be administered at Visits 1 and 4, and comparisons will be made between the two groups for each visit.

#### Korean version of the pittsburgh sleep quality index.

The Korean version of the Pittsburgh Sleep Quality Index, a self-reported questionnaire, evaluates seven components associated with sleep quality, including subjective sleep quality, sleep latency, sleep duration, habitual sleep efficiency, sleep disturbances, use of sleeping medication, and daytime dysfunction [28]. The global score is determined by summing the scores within the range of 0–21. Global scores will be appraised at Visits 1 and 4, and comparisons will be made between the two groups for each visit.

#### Korean-montreal cognitive assessment.

The Korean-Montreal Cognitive Assessment, a cognitive function assessment tool, comprehensively evaluates various aspects such as visuospatial/executive functions, naming, attention, language, abstraction, orientation, and delayed recall [29]. The maximum score is 30 points, and the normal range is > 23 points. The total scores will be computed during Visits 1 and 4. Comparative analyses will be performed between the two groups for each visit.

#### Beck depression inventory.

The Beck Depression Inventory will be used as an assessment tool for depression and its severity post-COVID-19 or recovery. It comprises 21 items classified into cognitive, emotional, motivational, and physical symptoms of depression, and each item is rated on a scale of 0–3. The cumulative score ranges from 0–63, with higher scores signifying more severe depression symptoms [30]. Beck Depression Inventory assessments will be conducted during Visits 1 and 4, and comparative analyses will be performed between the two study groups for each visit.

#### Digit span test in the Korean-Wechsler adult intelligence scale.

The Korean-Wechsler Adult Intelligence Scale, a widely used intelligence test tool in clinical practice [31], will be employed to evaluate changes in cognitive impairment associated with cognitive dysfunction. A subset of the Korean-Wechsler Adult Intelligence Scale, the digit span test, is a well-validated and convenient measure for assessing verbal short-term memory and working memory capacity. This test comprises two components: digits forward (DF) and digits backward (DB). In the DF component, subjects repeat a series of spoken digits (typically 3–9 digits) in their original order. In the DB component, subjects repeat a series of digits (2–8 digits) in reverse order. The test assesses whether the subject provides consecutive incorrect responses. The DF score indicates the number of digits in the largest number correctly repeated, and the DB score indicates the number of digits in the largest number correctly repeated in reverse order. The higher the DF and DB scores, the better the subject’s short-term memory. The difference between the DF and DB score is also calculated, and if it exceeds 5, this signifies abnormal working memory. The digit span test will be administered during Visits 1 and 4, with subsequent comparisons conducted between the two groups for each visit.

#### Computerized neurocognitive function test (CNT).

A CNT is a tool to evaluate neurocognitive functions, such as language ability, memory, attention, planning, thinking ability, and motor ability. This study will use CNT40, which was developed in South Korea and is in clinical use, to evaluate cognitive decline in patients with long COVID. It comprises five components to evaluate attention and higher cognitive functions. [32]. A language sustainability test, conditional language continuous test, visual continuous test, and conditional visual continuous test will be administered. CNT will be conducted at Visits 1 and 4, and comparisons will be made between the two groups for each visit.

#### Short physical performance battery.

The Short Physical Performance Battery evaluates the physical function of older adults or those who are weak after illness. It consists of a static balance test, Gait speed test, and stand-up test [33] and will be conducted to evaluate physical function decline and functional recovery in patients with long COVID. An AndanteFit (Dyphi Inc., Daejeon, Korea) multisensor-based kiosk, which comprises devices for the performance of gait speed, stand-up, and static balance tests, will be used for accurate measurements. The Short Physical Performance Battery will be conducted at Visits 1 and 4, and comparisons will be made between the two groups for each visit.

#### Feasibility evaluation.

The subject recruitment rate will be determined by calculating the ratio of the overall number of recruited subjects during the study duration to the intended number of study participants (100 subjects: n =  50 in the Kyungok-go and placebo groups, respectively). The study enrollment rate will be derived by dividing the total number of study subjects by the total number of participants who undergo screening. Detailed documentation of the reason for any dropouts will be systematically recorded. The dropout rate will be calculated and compared for all study subjects, as well as individually for each group.

#### Final treatment success rate.

Fatigue symptoms will be assessed on a visual analog scale (0–100 mm) before (Visit 1) and after (Visit 4) intervention administration for 12 weeks. Treatment success will be defined as a difference of more than 15 points, and the frequency and ratio of subjects successfully treated in both groups will be presented [34].

#### Subgroup analysis.

A subgroup analysis will be conducted according to the early warning score (EWS) to explore the differences in the effects based on the severity of COVID-19. The EWS evaluation will involve assessing oxygen saturation, supplemental oxygen usage, heart rate, systolic blood pressure, respiratory rate, body temperature, and level of consciousness [35]. The study participants will be classified into a mild group (EWS < 5) or a severe group (EWS ≥ 5) [36]. This analysis will focus on the primary outcome variable (FSS scores after 12 weeks of intervention).

#### Safety evaluation.

Safety will be assessed with a comprehensive evaluation of various parameters, including adverse events, vital signs, laboratory test results, and electrocardiograms per protocol. Clinically significant abnormal observed values and changes from baseline will be investigated. Adverse events will include any harmful and unintended symptoms, abnormal laboratory test results, or diseases that occur in subjects in the Kyungok-go group, even if there is no direct causal relationship. Study subjects or their representatives will voluntarily and promptly report any adverse effects.

### Statistical analysis

Statistical analysis will be performed using R-software (latest version, retrieved from https://www.R-project.org/) or Jamovi software (latest version, retrieved from https://www.jamovi.org).All statistical tests will be two-sided with a significance level of 5% (p-values <  0.05). In cases of missing data in the full analysis set during the efficacy evaluation, missing data will be imputed using the last observation carried forward method. For other cases, statistical analysis will be conducted using the original data.

### Definitions

*Safety set*: The group of subjects who have received the investigational drug at least once and have undergone safety-related tracking observations at least once.
*Full analysis set:* According to the intention-to-treat principle, this set includes subjects who have undergone measurements for the primary efficacy evaluation variables at least once after receiving the investigational drug.

### Efficacy evaluation

The data for the efficacy evaluation will be primarily analyzed using the full analysis set. For the analysis of efficacy evaluation variables, an analysis of covariance will be conducted with the assigned group as a factor and baseline values as covariates to test the intergroup differences. If conditions for the assumptions of analysis of covariance, such as normality and homogeneity of variance, are not met or if outliers are observed, a rank analysis of covariance will be performed. For categorical data, the frequency and proportion in each group will be calculated for intergroup comparisons, and analysis will be performed using either chi-square test or Fisher exact test (when expected frequencies are ≤ 5).

### Feasibility evaluation

The analysis of feasibility evaluation factors, such as subject recruitment rate, subject enrollment rate, dropout rate, and reasons for dropout will be calculated as the total number of study subjects divided by the total number of participants screened (%). The dropout rate will be calculated for the entire study population and for each intervention group, and a descriptive analysis, including the frequency of dropout and reasons, will be conducted.

### Safety evaluation

Data for the safety evaluation will be assessed using the safety set. Adverse reactions are defined as any non-harmful signs, symptoms, or diseases observed in subjects receiving investigational drugs during clinical trials. For each intervention group, the occurrence of all adverse reactions, drug-related adverse reactions, serious adverse reactions, and serious drug-related adverse reactions is presented, including the number of patients, incidence, manifestation counts, and incidence rates with a 95% confidence interval. Differences between treatment groups are tested using Chi-square test or Fisher’s exact test. All adverse reactions are coded using the Medical Dictionary for Regulatory Activities (MedDRA) terms for system organ class and preferred terms, providing frequencies, proportions, and counts for each group. Vital signs, quantitative laboratory test results, and heart rate variability are presented for each herbal medicine group before and after administration. Descriptive statistics are used to show changes, and differences between groups are assessed with independent two-sample t-tests or Wilcoxon rank-sum tests. Intragroup differences in changes are examined using paired t-tests or Wilcoxon signed-rank tests. Additionally, laboratory test results are presented in contingency tables for normal (including clinically insignificant abnormal) and clinically significant abnormal changes before/after herbal medicine administration. McNamar’s test (or McNamar’s Exact test) is utilized to test intragroup differences in changes. These analyses aim to quantitatively evaluate clinical trial outcomes and assess differences within and between treatment groups.

### Ethics and dissemination

The studies involving human participants were reviewed and approved by the Institutional Review Board of Kyung Hee University Korean Medicine Hospital (IRB approval numbers KOMCIRB 2023-04-005-002). This trial has been registered with Clinical Research Information Service (CRIS). The trial registration number was KCT0008789 (https://cris.nih.go.kr/cris/search/detailSearch.do/25409). The pilot study protocol and final results will be disseminated to the public in peer-reviewed journals.

## Discussion

This clinical trial will be a randomized, double-blind, multicenter study designed to assess the efficacy and safety of Kyungok-go to treat fatigue in patients with long COVID.

The development of post-COVID syndrome involves multiple factors, and several mechanisms may contribute to a wide range of clinical manifestations. Prolonged inflammation plays a significant role in its pathogenesis and may be responsible for certain neurological complications, cognitive dysfunction, and several other symptoms [37]. There are reports of chronic fatigue syndrome observed in patients with various viral infections, and survivors of the SARS outbreak in 2003 showed symptoms corresponding to chronic fatigue syndrome, some of which persisted for years after infection [38]. Therefore, the fatigue symptoms of long COVID can be considered in the category of postviral fatigue syndrome, suggesting that they share similarities with other viral-induced fatigue conditions [10].

Preclinical studies have shown that Kyungok-go significantly reduced blood lactate levels, increased blood glucose levels, and increased glycogen levels in skeletal muscles when administered to ICR mice for 4 weeks. It also exhibited antifatigue effects and led to improved exercise performance, as evidenced by increased grip strength and treadmill exercise capacity and increased duration of forced swimming exercise. These findings suggest that Kyungok-go exerts an antifatigue effect [39]. There is clinical evidence that Kyungok-go may be effective in improving post-exertional malaise and postexercise recovery, even after progressive exercise, and it has been reported to increase maximal oxygen consumption and improve postexercise heart rate recovery in soccer players [40]. Because liver function test results (i.e., AST and ALT) showed no significant difference between the group that took Kyungok-go for 4 weeks and exercised and the control group and both groups showed values within the normal range (<40 U/L), liver function impairment was not considered an issue. Kyungok-go has also been identified as a potential treatment for neurological inflammation and neurodegenerative diseases because it suppressed nitric oxide, inducible nitric oxide synthase, cyclooxygenase 2, and various cytokines that occur during inflammation in BV2 cells, a microglial mouse cell line [41]. A review of the properties of Kyungok-go reported that it possessed antioxidant, anticancer, and anti-inflammatory properties; enhanced immunity; and promoted growth; all of which can be applied to various diseases of the central nervous system, cardiovascular system, digestive system, and respiratory system without toxicity or side effects [13].

The therapeutic effects of traditional medicine are often achieved using multiple ingredients to influence multiple targets. For complex chronic illnesses such as cancer, immune disorders, mental illnesses, cardiovascular diseases, and lifestyle diseases, a multicomponent–multitarget–multipathway approach, which involves controlling multiple target sites, has proven more effective than conventional single-component drugs [42]. SARS-CoV-2 enters cells via the angiotensin-converting enzyme 2 receptor, leading to complications in multiple organs [43]. Once the virus infects these cells, it causes extensive damage, resulting in various persistent symptoms [44]. Consequently, long COVID is characterized by the involvement and impairment of the structure and function of multiple organs in affected individuals. Since Kyungok-go possesses various biological activities, it is expected to exert a range of therapeutic effects on fatigue.

Considering the current circumstances of a high rate of patients experiencing long COVID, this study aims to administer Kyungok-go for a maximum of 12 weeks to patients experiencing fatigue after recovering from COVID-19 and investigate the potential amelioration of symptoms associated with herbal medicine. Overall, this trial will provide evidence of the efficacy and safety of Kyungok-go for treating fatigue in patients with long COVID. Although this study may offer insights into the feasibility of using herbal medications for post-acute sequelae of COVID-19, it is crucial to emphasize that additional RCTs with formal sample size calculations are essential to establish robust clinical evidence regarding the efficacy and safety of these interventions.

## Conclusion

This pilot study will evaluate the feasibility of the trial protocol and provide preliminary data on the efficacy and safety of Kyungok-go for treating fatigue among long COVID patients. The results will be used to determine the appropriate sample size and design for a full-scale RCT.

## Supporting information

S1 File SPIRIT checklist. (DOCX)

S2 File Study protocol. (PDF)

S3 File Study protocol (KOR). (PDF)
