# Supplementary material for: Kyungok-go for fatigue in patients with long COVID: Double-blind, randomized, multicenter, pilot clinical study protocol
Source: PLoS One. 2025 Apr 1;20(4):e0319459. doi: 10.1371/journal.pone.0319459 (PMC11960999; doi:10.1371/journal.pone.0319459)
Supplement: S2 File — (PDF) [file pone.0319459.s002.pdf]

Clinical Trial Protocol

Effectiveness of Kyungokgo on Fatigue in Long COVID Patients: A Double-Blind Randomized Multi-Center Pilot Clinical Study

**Protocol Dumber**	CV2023
**Sponsor**	Korea Institute of Oriental Medicine
**Version Number**	1.2
**Version Date**	2023.06.08

Table of Contents

CLINICAL TRIAL PROTOCOL 1

LIST OF ABBREVIATIONS 10

1. CLINICAL TRIAL TITLE AND PHASE 12

2. NAME AND ADDRESS OF CLINICAL TRIAL INSTITUTION 12

3. PRINCIPAL INVESTIGATOR..12

4. SPONSORING AGENCY 12

5. BACKGROUND AND PURPOSE OF CLINICAL TRIALS 13

6. TARGET DISEASES 19

7. PARTICIPANT SELECTION 19

8. STANDARD TREATMENT METHODS FOR TARGET DISEASE 20

9. INVESTIGATIONAL DRUG USED IN THE CLINICAL TRIALS 22

10. STUDY DURATION 26

11. STUDY METHODS 26

12. PRECAUTIONS FOR USE OF THE INVESTIGATIONAL DRUGS 44

13. CRITERIA FOR DISCONTINUATION AND WITHDRAWAL OF RESEARCH SUBJECTS, CRITERIA FOR CLINICAL TRIAL TERMINATION 45

14. STATISTICAL ANALYSIS METHODS 46

15. CRITERIA, METHODS, AND INTERPRETATION FOR EFFICACY EVALUATION .48

16. CRITERIA, METHODS, AND INTERPRETATION FOR SAFETY EVALUATION INCLUDING ADVERSE REACTIONS..51

17. PROVISIONS FOR PARTICIPANT COMPENSATION..55

18. INFORMED CONSENT 55

19. ACTIONS IN CASE OF ADVERSE REACTIONS 55

20. MEASURES FOR PROTECTING PARTICIPANT SAFETY 56

21. OTHER NECESSARY ITEMS FOR SAFE AND SCIENTIFIC CONDUCT OF THE CLINICAL TRIAL 57

22. BENEFITS OF PARTICIPATING IN CLINICAL TRIALS 59

23. TRANSPORT, STORAGE, ANALYSIS, AND DISPOSAL OF SPECIMENS 60

24. POLICIES ON REPORT SUBMISSION AND PUBLICATION 60

25. PROTECTION MEASURES FOR VULNERABLE PARTICIPANTS 60

26. RECRUITMENT PLAN FOR PARTICIPANTS 60

27. RISK/BENEFIT ASSESSMENT 60

28. ETHICAL ASPECTS OF THE CLINICAL TRIALS 61

REFERENCES 62

Protocol version history

**Protocol ver. 1.0**	**2023.02.10**
**Protocol ver. 1.1**	**2023.05.09**
**Protocol ver. 1.2**	**2023.06.08**

Summary of the Clinical Trial

**Title**	Evaluating the Effectiveness of Kyungok-go on Fatigue in Long COVID Patients: A Double-Blind Randomized Multi-Center Pilot Clinical Study
**Institutions and Principal Investigators**	Kyung Hee University Korean Medicine Hospital: Professor Kim Tae-hoon<br><br>Dong-eui University Korean Medicine Hospital: Professor Kwon Chan-young
**Sponsor**	Korea Institute of Oriental Medicine
**Objective**	This prospective pilot clinical trial aims to explore the efficacy and safety of the traditional Korean medicine Kyungok-go in patients with Long COVID, specifically targeting physical fatigue as the main symptom. Patients will be randomly assigned to the Kyungok-go group or the placebo group and will be administered the treatment for 12 weeks. The primary objective is to assess changes in the Fatigue Severity Scale (FSS) scores after 12 weeks of treatment. Additionally, the study will evaluate the feasibility of the study design, recruitment rates, dropout rates, and the impact of Kyungok-go on COVID-19 immune response and fatigue-related metabolites.
**Target disease**	Fatigue
**Inclusion and Exclusion Criteria**	**1) Inclusion Criteria**<br>● Adults aged 19 years or older and at least 12 weeks have passed since being diagnosed with COVID-19<br>● A person who has continuously experienced fatigue for more than 4 weeks that was not experienced before being diagnosed with COVID-19<br>● Those with an FSS score of 4 or higher<br>● Those who have no problems with overall cognitive function and who voluntarily agree to participate in the study through written consent<br>**2) Exclusion Criteria**<br>● History or current presence of conditions causing fatigue (cancer, sleep disorders, chronic hepatitis, liver cirrhosis, chronic kidney disease, tuberculosis, asthma, multiple sclerosis).<br>● Diseases that may affect drug administration or absorption (dysphagia, clinically significant digestive disorders, galactose intolerance, Lapp lactase deficiency, genetic problems like glucose-galactose malabsorption)<br>● uncontrolled diabetes.<br>● History of allergy to Kyungok-go.

<table>
<tr><td></td><td>● Liver or kidney disease history or lab results exceeding 3 times the upper normal limit for AST, ALT, BUN, Creatinine.
● Women who are pregnant, may become pregnant, or are breastfeeding
● Participation in another study within the last 30 days
● Clinically significant psychiatric symptoms or medical conditions as judged by the investigator</td></tr>
<tr><td>Target Number of Participants</td><td>This pilot study aims to recruit 100 participants, with 50 in the Kyungok-go treatment group and 50 in the placebo group.</td></tr>
<tr><td>Method</td><td>Participants will be screened for eligibility after providing informed consent. Eligible participants will be randomized into the treatment group (Kyungokgo) or placebo group and will receive the intervention for 12 weeks. Monthly visits will be conducted for assessment, and post-treatment evaluations will include symptom and hematologic, immunologic changes.</td></tr>
<tr><td>Duration</td><td>The clinical trial will span 3 years from the start date.</td></tr>
<tr><td>Investigational Medicine and Administration</td><td><table><tr><th>Code name (herbal medicine name)</th><th>usage</th><th>One-time administration unit (dose)</th></tr><tr><td>CV1 (Kyungok-go)</td><td>●/○/●</td><td>1 packet (22.5g)</td></tr></table>●Take, ○Do not take</td></tr>
<tr><td>Efficacy Evaluation Variables</td><td>Primary endpoint
1) Change in FSS score after 12 weeks of intervention.
Secondary endpoint
1) Final medication adherence.
2) Differences in final medication adherence between groups.
3) FSS scores at visits 2 and 3.
4) ChFS total and subscale scores at visits 2, 3, and 4.
5) EQ-5D-5L scores at visit 4.
6) PSQI-K Global score at visit 4.
7) K-MOCA total and domain scores at visit 4.
8) BDI scores at visit 4.
9) DF, DB, and DF-DB scores at visit 4.
10) Computerized neurocognitive function test scores at visit 4.
11) SPPB test scores for walking speed, standing test, static balance test, and total score at visit 4.
12) Analysis of recruitment rates, dropout rates, and reasons for dropout.</td></tr>
</table>

	13) Treatment success rate comparing intervention and placebo groups after 12 weeks, defined as a VAS difference of 15 points or more.
**Safety Evaluation variable**	1) Adverse reactions reported during or after the intervention<br>2) Abnormal findings during physical exams<br>3) Blood chemistry and ECG tests
**Statistical Analysis Method**	**General Principles of Outcome Analysis**<br>Data for efficacy evaluation will primarily use the Full Analysis Set (FAS). Data for safety evaluation will be assessed from the Safety Set (SS). All statistical tests, unless otherwise specified, will be two-tailed with a 5% significance level. For efficacy evaluation, in cases where there are missing values in the FAS, the Last Observation Carried Forward (LOCF) method will be applied for statistical analysis, and all other analyses will be conducted with the original data.<br><br>**Full Analysis Set (FAS):** Following the ITT principle, this set includes participants who have received the herbal medication and have at least one measurement of the primary efficacy variable.<br>**Safety Set (SS):** This set includes participants who have received the intervention at least once and have undergone safety-related follow-up at least once.<br><br>**Analysis of Primary Endpoint**<br>1) FSS Scores After 12 Weeks of Intervention<br>: FSS scores are compared between groups after taking 12 weeks of intervention.<br><br>**Analysis of Secondary Endpoints**<br>1) Final Medication Adherence<br>: Present the mean, standard deviation, median, minimum, and maximum values of the final medication adherence rate (%) for all participants at 12 weeks (Visit 4 or termination point).<br><br>2) Difference in Final Medication Adherence Between Groups<br>: Present and compare the mean, standard deviation, median, minimum, and maximum values of the final medication adherence rate (%) for both groups at 12 weeks (Visit 4 or termination point).

	3) FSS Scores at Visits 2 and 3<br>: Compare the FSS scores between groups at each of these time points.<br>4) ChFS Scores and Subscale Scores at Visits 2, 3, and 4<br>: Record the total score and separate physical (items 1-7) and mental health (items 8-11) subscale scores, and compare these between groups at each time point.<br>5) EQ-5D-5L Scores at Visit 4<br>: Calculate and compare the EQ-5D-5L scores between groups at this time point.<br>6) PSQI-K Global Score at Visit 4<br>: Calculate the total Global score from the 7 component scores on the PSQI-K questionnaire and compare these between groups at this time point.<br>7) K-MOCA Total and Domain Scores at Visit 4<br>: Calculate the scores for the 7 domains, sum these to obtain the total score, and compare these between groups.<br>8) BDI Scores at Visit 4<br>: Sum the scores for the 4 domains to obtain the total score and compare these between groups.<br>9) DF, DB, and DF-DB Scores at Visit 4<br>: Compare these scores between groups at this time point.<br>10) Computerized Neurocognitive Function Test Scores at Visit 4<br>: Compare the scores from the 5 tests between groups.<br>11) SPPB Test Scores at Visit 4<br>: Compare the walking speed test, sit-to-stand test, static balance test scores, and the overall score between groups.<br>12) Analysis for Feasibility Evaluation<br>: Calculate the recruitment rate based on the number of participants recruited during the study period out of the planned number of participants. Calculate the registration rate by dividing the total number of study participants by the total number of screened participants. Calculate and compare the dropout rates and reasons for dropout for the overall study participants and for each group.<br>13) Treatment Success Rate Between the Intervention and Control Groups After 12 Week

	: Define treatment success as a difference of 15 points or more on the 0-100 VAS for fatigue symptoms after 12 weeks of intervention.<br><br>**Analysis of Safety Variables**<br><br>**Adverse Reactions**<br>Adverse reactions are defined as any unfavorable symptoms, signs, or diseases that occur in study participants after administration of the investigational product. Present the number of patients, incidence rate, frequency, and 95% confidence interval for all adverse reactions, adverse drug reactions (ADR), serious adverse reactions (SAE), and serious adverse drug reactions (SADR) for each intervention group. Differences between groups will be tested using the Chi-square test or Fisher's exact test. Code all adverse reactions using the MedDRA (Medical Dictionary for Regulatory Activities) by System Organ Class (SOC) and Preferred Term (PT), and present the frequency, proportion, and number of events for each group.<br><br>**Laboratory Tests, Vital Signs**<br>Present descriptive statistics for pre- and post-intervention values and changes in vital signs and quantitative laboratory test results for each group. Compare changes between groups using the independent two-sample t-test or Wilcoxon rank-sum test, and changes within each group using the paired t-test or Wilcoxon signed-rank test. Additionally, present contingency tables for dichotomous laboratory test results and ECG results showing changes from pre- to post-intervention categorized as normal (including clinically insignificant abnormal) or clinically significant abnormal. Test changes within each group using McNemar's test (or McNemar's Exact test).

List of Abbreviations

**ADR**	**Adverse Drug Reaction**
**A.E.**	**Adverse Event**
**ALT**	**Alanine Transaminase**
**AST**	**Aspartate aminotransferase**
**BDI**	**Becks' depression inventory**
**BMI**	**Body Mass Index**
**BUN**	**Blood Urea Nitrogen**
**C.K.**	**Creatine Kinase**
**CFS**	**Chronic Fatigue Syndrome**
**ChF S**	**Chalder Fatigue Scale**
**C OVID-19**	**Coronavirus disease 2019**
**CRP**	**C-Reactive Protein**
**DB**	**Digit span backward**
**D F**	**D igit span forward**
**EPO**	**Erythropoietin**
**EQ-5D**	**EuroQol five dimensions questionnaire**
**FAS**	**Full Analysis Set**
**FSS**	**F atigue severity scale**
**F /U**	**F ollow-up**
**GCP**	**Good Clinical Practice**
**Hb**	**Hemoglobin**
**HBV**	**Hepatitis B Virus**
**Hct**	**Hematocrit**
**HCV**	**Hepatitis C Virus**
**HIV**	**Human Immunodeficiency Virus**
**ICH**	**International Conference on Harmonization**
**IRB**	**Institutional Review Board**
**ITT**	**Intent To Treat**
**K-MOCA**	**Korean-Montreal cognitive assessment**
**K-WAIS-IV**	**Korean Wechsler Adult Intelligence Scale-IV**
**LDH**	**Lactate Dehydrogenase**
**LOCF**	**Last Observation Carried Forward**
**MedDRA**	**Medical Dictionary for Regulatory Activities**
**PSQI-K**	**English version of Pittsburgh Sleep Quality Index**
**R.B.C.**	**Red Blood Cell**
**SADR**	**Serious Adverse Drug Reaction**
**S.A.E.**	**Serious adverse event**
**SPPB**	**S hort physical performance battery**
**TSH**	**Thyroid Stimulating Hormone**
**U.N.L.**	**Upper Normal Limit**
**WBC**	**White Blood Cell**

Schedule Summary

Visit	0	One	2	3	4	UV *
Week (day)	-1 (-7)	1(0)	4 (28)	8 (56)	1 2 (84)	-
Visit window (day)	-	+2	±2	±2	±2	-
Written Consent	●					
Demographic Survey	●					
Participation in Other Clinical Trials	●					
Physical Exam	●					
Medical History	●	●				
COVID-19 Diagnosis	●					
Concomitant Medication/Treatment	●					
Vital Signs	●	●	●	●	●	●
Electrocardiogram	●				●	●
Laboratory Test	●				●	●
Immune, Metabolite Test (Blood Collection)	●				●	●
Pregnancy Status Evaluation **	●					
Fatigue Symptom Evaluation ***	●					
FSS (Fatigue Severity Scale)	●		●	●	●	●
Inclusion and Exclusion Criteria Evaluation	●					
Random Assignment		●				
Prescription and Delivery of Assigned Intervention (Kyungok-go or placebo)		●	●	●		
Compliance check			●	●	●	●
Combined Medication Evaluation			●	●	●	●
0-100 VAS (Visual Analogue Scale) of Fatigue		●	●	●	●	●
CHFS (Chalder Fatigue Scale)		●	●	●	●	●
EQ-5D-5L		●			●	●
PSQI- K (Korean version of Pittsburgh Sleep Quality Index)		●			●	●
K- MOCA (Korean-Montreal Cognitive Assessment)		●			●	●
BDI (Becks' Depression Inventory)		●			●	●
Test for Memorizing Numbers Straight/Reverse		●			●	●
Computerization Neurocognitive Function Test Score		●			●	●
SPPB (Short Physical Performance Battery)		●			●	●
Adverse Reaction Inspection			●	●	●	●

● Conducted at that visit ; *UV: Unscheduled visit (visiting on a day other than the original visit schedule due to adverse reactions, etc.); * * Only for women of childbearing age ; *** To evaluate the selection criteria, we investigated whether fatigue occurred after becoming sick with COVID-19.

1. Title and Phase of the Clinical Trial

Clinical Trial to Evaluate the Efficacy of Kyungok-go for Fatigue in Patients with Long COVID:

A Double-Blind, Randomized, Multi-Center, Preliminary, Investigator-Initiated Trial

Phase : Not applicable

2. Names and Addresses of Clinical Trials Institutions

Kyung Hee University Korean Medicine Hospital (23 Kyungheedae-ro, Dongdaemun-gu, Seoul, South Korea)

Dong-Eui University Korean Medicine Hospital (62 Yangjeong-ro, Busanjin-gu, Busan, South Korea)

3. Principal Investigators of the Clinical Trials

Institution		Name	Position
Kyunghee University Korean Medicine Hospital	Korean Medicine Clinical Trial Center	Tae-Hoon Kim	Professor
Dongeui University Korean Medicine Hospital	Korean Medicine Neuropsychiatry	Chan-Young Kwon	Professor

4. Supporting Institution for the Clinical Trial

Korea Institute of Oriental Medicine (1672 Yuseong-daero, Yuseong-gu, Daejeon, 042-861-1994)

5. Background and Purpose of the Clinical Trial

1) Research background

Status of Post-COVID-19 Sequelae

Due to the global pandemic of coronavirus disease-19 (COVID-19), medical and research efforts have been concentrated on developing vaccines to prevent infection, halting the spread of the virus, and treating acute infections. However, in addition to the life-threatening acute infections, post-COVID-19 sequelae are also being widely reported. A research team from the University of Michigan conducted telephone interviews to investigate the presence of sequelae in 1,250 severe COVID-19 patients who were hospitalized in 38 hospitals in Michigan, USA, from spring to summer 2020. Among the 488 respondents, about 39% reported that they could not perform normal activities even two months after discharge, and 12% stated that it was difficult to manage daily activities on their own as they did before contract COVID-19. Additionally, 23% reported experiencing shortness of breath when climbing stairs, and one-third mentioned persistent acute symptoms such as loss of taste or smell. Moreover, 40% of the respondents were unable to return to their previous jobs due to health issues, with some becoming unemployed, and 26% of those who returned to work had to reduce their working hours due to health problems [1] . These findings highlight the severe impact of post-COVID-19 sequelae, comparable to the acute symptoms of the disease.

A recent study on the prevalence of post-COVID-19 sequelae reported that fatigue is the most common symptom, affecting 28.4% of hospitalized patients and 34.5% of outpatients among 735,006 COVID-19 patients [2] . Researchers at Trinity College Dublin conducted a follow-up study on 128 COVID-19 patients who were either hospitalized or received outpatient treatment at St. James's Hospital in Dublin. Over half of these patients (52.3%) reported experiencing fatigue for at least six weeks after being diagnosed with COVID-19, with some severe cases

experiencing fatigue for up to 10 weeks. In contrast, 54 respondents (42.2%) reported no post-COVID-19 sequelae. Interestingly, the study found no correlation between the severity of COVID-19 and the level of fatigue, indicating that more than half of the COVID-19 survivors experienced fatigue regardless of the severity of their initial symptoms [3] . The symptoms experienced after recovering from COVID-19 are highly variable, and their mechanisms are not yet clearly understood. However, chronic fatigue syndrome (CFS) has been observed in patients who have suffered from various viral infections. Approximately 27% of survivors of the 2003 SARS outbreak were reported to exhibit CFS symptoms years after their diagnosis [4] . Thus, it is reasonable to consider these symptoms as part of the broader category of post-viral fatigue syndrome.

This situation applies equally to South Korea. On September 29, the Central Disease Control Headquarters announced in a regular briefing that more than 90% of COVID-19 survivors experienced sequelae such as fatigue and decreased concentration. Deputy Director Kwon Jun-wook of the Central Disease Control Headquarters reported that in a survey conducted by Kyungpook National University Hospital involving 5,762 participants, 965 respondents provided answers regarding post-COVID-19 sequelae, with 91.1% (879 respondents) indicating that they experienced at least one sequela. The most common sequelae reported in South Korea include fatigue (26.2%) and decreased concentration (24.6%), as well as psychological and mental sequelae, and loss of smell and taste [5] . This indicates a growing need for treatment strategies for post-COVID-19 sequelae as part of the overall management of the disease.

Hypothetical Mechanism and Conventional Treatment of Long COVID

There is no universally established definition for what is commonly referred to as Long COVID, with different countries and institutions using varying terms and definitions. The World Health Organization (WHO) defines Post-COVID Conditions as symptoms that cannot be explained by an alternative diagnosis, occurring within three months of COVID-19 symptom onset and lasting for at least two months[6] . The U.S. Centers for Disease Control and Prevention (CDC) defines Post-COVID Conditions (Long COVID) as symptoms persisting for more than four weeks after the initial infection. The National Institute for Health and Care Excellence (NICE) in the UK defines Ongoing symptomatic COVID-19 as symptoms lasting 4-12 weeks, and Post-COVID syndrome as symptoms persisting beyond 12 weeks and not explained by other diagnoses[7] .

In South Korea, there is a need to unify terminology for research and case definitions related to post-COVID-19 sequelae. Through discussions between the Korea Disease Control and Prevention Agency (KDCA) and the Korean Society of Infectious Diseases, Long COVID has been defined as symptoms or signs lasting beyond 12 weeks after COVID-19 diagnosis that cannot be explained by other diseases. Acute or subacute complications of COVID-19, such as venous thromboembolism, myocarditis, pericarditis, encephalitis, and thyroiditis, are not classified as Long COVID. The symptoms commonly associated with Long COVID include fatigue, shortness of breath, depression, anxiety, and cognitive decline[8] .

Fatigue

Fatigue is one of the common non-respiratory symptoms experienced by COVID-19 patients, with approximately 41% reporting it. It is reported to occur in 35-45% of patients at four weeks, 30-77% at eight weeks, and 16-55% at twelve weeks post-infection. The fatigue experienced by COVID-19 patients resembles chronic fatigue syndrome (CFS) described after

Middle East respiratory syndrome (MERS), severe acute respiratory syndrome (SARS), or community-acquired pneumonia. However, there has been no reported correlation between the severity of COVID-19 and long-term fatigue symptoms or related laboratory inflammatory markers. Excessive fatigue has been noted in female patients previously diagnosed with depression or anxiety. The fatigue associated with Long COVID can be more extensive and severe, including symptoms such as sore throat, generalized pain, changes in blood pressure, gastrointestinal issues like irritable bowel syndrome, headaches, sleep disturbances, depression, and dizziness. More severe neurological symptoms such as new sensitivities or allergies, burning or tingling sensations in the limbs, may also occur. A key characteristic of this condition is the sudden worsening of symptoms with minimal physical or mental activity. Since these symptoms align with chronic fatigue syndrome (also known as myalgic encephalomyelitis or ME), the WHO classifies these symptoms within the same category of neurological disorders. Clinical evaluations for post-viral fatigue syndrome are conducted similarly to those for chronic fatigue syndrome.

The exact mechanisms behind post-viral fatigue syndrome are not yet fully understood. Hypotheses include abnormal reactions to latent viruses in the body, increased levels of pro-inflammatory cytokines, and neuroinflammation. Specifically, the phenomenon known as a 'cytokine storm,' involving a surge of cytokines like IL-2, IL-7, granulocyte, interferon-gamma, monocyte, and TNF-alpha triggered by viruses, leading to multi-organ damage, can result in long-term functional impairment post-recovery. This may lead to disorders such as post-encephalitis syndrome, with disruptions in cytokine signaling pathways causing symptoms like fatigue. Therefore, a holistic approach is likely needed to recover immune function and improve organ function in individuals experiencing Long COVID[9] . However, there is currently

a lack of high-level evidence supporting the effectiveness of treatments and interventions for managing fatigue in Long COVID patients. As a result, symptomatic and lifestyle management remains the primary mode of treatment, highlighting an urgent need for further research in this area[8] .

Evidence for Kyungok-go in Treating Post-COVID-19 Sequelae

Literature studies on chronic fatigue syndrome in traditional Korean medicine identify several patterns, including spleen qi deficiency, liver-spleen disharmony, qi deficiency with blood stasis, liver-kidney yin deficiency, and spleen-kidney yang deficiency, with spleen qi deficiency being the most common pattern[10] . A survey of Korean traditional medicine practitioners revealed that herbal medicine is the most effective and widely used treatment for chronic fatigue syndrome and idiopathic chronic fatigue, with formulas such as Bojungikgi-tang, Ssanghwa-tang, Sipjeondaebo-tang, Guipi-tang, Palmul-tang, Gongjin-dan, and Kyungok-go frequently selected for treatment[11] . Kyungok-go is clinically used to improve fatigue and related symptoms after illnesses characterized by qi and yin deficiency.

In an experimental study, ICR mice treated with 600 mg/kg of Kyungok-go for four weeks showed significant reductions in blood lactate, increased blood glucose levels, increased glycogen content in skeletal muscle, improved grip strength, enhanced treadmill exercise performance, and prolonged forced swimming exercise duration, indicating anti-fatigue effects and improved exercise performance[12] . Clinical evidence suggests that Kyungok-go is effective in reducing lactate and ammonia levels after incremental exercise, and it has been shown to increase maximal oxygen uptake and heart rate recovery after exercise, aiding in aerobic exercise capacity and post-exercise recovery in soccer players[13] [14] .

Moreover, Kyungok-go was the most prescribed herbal medicine for COVID-19 treatment in Korea during the first half of 2020, and it has been reported to improve COVID-19 related symptoms, including fatigue [15] . tudies also suggest potential applications of Kyungok-go in managing neuroinflammation and neurodegenerative diseases. In BV2 microglial cells, Kyungok-go was found to inhibit the production of NO, iNOS, COX-2, and various cytokines during inflammation [16] . Additionally, literature on Kyungok-go indicates its antioxidant, anticancer, anti-inflammatory, immune-boosting, and growth-promoting effects, making it applicable to a variety of conditions in the central nervous, cardiovascular, digestive, and respiratory systems without significant toxicity or side effects [17] . Kyungok-go is listed in the Korea Ministry of Food and Drug Safety as a herbal medicine indicated for conditions such as physical weakness and fatigue, suggesting its potential application in alleviating fatigue symptoms associated with Long COVID. Establishing clinical evidence for this application is crucial for developing traditional Korean medicine management strategies for Long COVID.

2) Purpose of the Study

The purpose of this study is to conduct a prospective preliminary clinical trial to evaluate the safety and efficacy of an over-the-counter herbal medicine in individuals who experience fatigue as a symptom of Long COVID after recovering from COVID-19. The trial aims to assess the feasibility of the intervention and research design for future large-scale studies. The primary objective is to explore changes in the Fatigue Severity Scale (FSS) after 12 weeks of taking the herbal medicine (Kyungok-go) in patients with Long COVID-related fatigue. The secondary objective is to evaluate the feasibility of the study design by monitoring recruitment rates and dropout rates during the study period. Additionally, the study aims to explore the

impact of the herbal medicine on COVID-19 immune responses and metabolites associated with fatigue in post-COVID-19 patients.

6. Target disease

Persistent fatigue following COVID-19 infection

7 . Subject selection

1) Inclusion criteria

- Adults aged 19 and above who have been diagnosed with COVID-19 for at least 12 weeks.
- Individuals who have experienced fatigue for more than 4 weeks that was not present before their COVID-19 diagnosis.
- Individuals with an FSS score of 4 or higher.
- Individuals with no significant cognitive impairments who voluntarily consent to participate in the study through written consent.

2) Exclusion criteria

- Individuals with a history or current condition of diseases that can cause fatigue (e.g., cancer, sleep disorders, chronic hepatitis, liver cirrhosis, chronic renal failure, tuberculosis, asthma, multiple sclerosis).
- Individuals with conditions that can affect drug absorption or metabolism (e.g., dysphagia, clinically significant gastrointestinal disorders, galactose intolerance, Lapp lactase deficiency, glucose-galactose malabsorption).
- Uncontrolled diabetic patients.
- Individuals with a history of allergies to the investigational medicinal product (herbal medicine).
- Individuals with a history of liver or kidney diseases, or blood tests showing AST, ALT, BUN,

or creatinine levels exceeding three times the upper limit of normal.

- Pregnant or breastfeeding women, or those with the potential to become pregnant.
- Individuals who have participated in another clinical trial within 30 days prior to the study.
- Individuals who have taken herbal or herbal medicine (containing the same ingredients as the investigational medicinal product) within two weeks prior to the study.
- Individuals whom the researcher deems unable to participate in the study due to clinically significant psychiatric symptoms, medical conditions, or laboratory findings.

3) Number of Subjects and Rationale

This study is a prospective pilot investigator-initiated clinical trial. The results obtained from this study will be used to calculate the number of participants for future confirmatory clinical trials. A total of 100 subjects will be recruited, with 50 in the treatment group (receiving Kyung-Ok-Go) and 50 in the control group (receiving placebo).

**Institute**	**Treatment group**	**Control group**	**Sum**
Kyunghee University Korean Medicine Hospital	30	30	60
Dongeui University Korean Medicine Hospital	20	2 0	40
Sum	50	50	100

8 . Standard Treatment Method for Target Disease

Various methods, including medications, alternative medicine, cognitive-behavioral therapy, and exercise, are currently being attempted to alleviate the fatigue symptoms following COVID-19 infection. Some studies have suggested that rintatolimod, a drug used to treat chronic fatigue syndrome, as well as counseling and graded exercise therapy, may offer

benefits, but the evidence for these interventions remains limited. The CDC describes the fatigue following COVID-19 under the category of Chronic Fatigue Syndrome/Myalgic Encephalomyelitis (CFS/ME), focusing on symptom management through gentle exercises such as meditation and yoga, counseling, dietary supplements, and rest [18] . The UK's NHS also recommends rest, reduced activity levels, graded rest and exercise, sleep management, a balanced diet, and concentration training as part of symptom management [19] .

9 . Investigational Medicinal Product

9.1 Investigational drugs Code name, Quantity of raw material of medicine , dosage form (appearance), etc.

Test drug

**Code name (product name)**	CV1 (Jin Kyungok-go)			
**Manufacture company**	Kyungjin Pharmaceutical Co., Ltd.			
**Route of administration**	Oral-			
**Form and appearance**	Black-brown viscous soft extract filled in aluminum stick pouches			
**Ingredients and Composition**	Per 112.5g of this medicine			
	Raw material name	standard	amount	unit
	Fresh Rehmannia glutinosa juice	Attachment specifications	39.9	g
	Poria	K.P.	12.4	g
	Ginseng	K.P.	6.2	g
	Honey	J P	41.5	g
**Expiration date**	36 months from date of manufacture			
**Storage method**	Airtight container, store at room temperature (1~30°C)			

placebo

**Code name (product name)**	P (placebo)
**Manufacture company**	Kyungjin Pharmaceutical Co., Ltd.
**Route of**	Oral-

**administration**				
**Form and appearance**	Black-brown viscous soft extract filled in aluminum stick pouches			
**Ingredients and Composition**	Per 112.5g of this medicine			
	Raw material name	standard	amount	unit
	Purified water	K.P.	65.0	g
	Sodium benzoate	K.P.	0.06	g
	Citric acid hydrate	K.P.	0.09	g
	Sodium citrate hydrate	K.P.	0.04	g
	High fructose 55	Attached specifications	21.0	g
	White sugar	K.P.	14.0	g
	Caramel	NF	5.0	g
	Xanthan gum	EP	3.8	g
	Concentrated glycerin	K.P.	3.5	g
	Ginseng Fureba A - 980306	Attached specifications	0.01	g
**Expiration date**	36 months from date of manufacture			
**Storage method**	Airtight container, store at room temperature (1~30°C)			

9.2 Rationale for Dosage Selection

The herbal medicine provided in this clinical trial is an authorized medicinal product approved by the Ministry of Food and Drug Safety. The dosage and administration method will strictly adhere to the approved specifications. As indicated in the product approval, CV1 (Jin Kyungok-go) will be administered at a dose of 22.5g (one stick) for adults, twice daily (morning and evening) on an empty stomach or between meals. The placebo will be administered in the

same manner as the test drug, at a dose of 22.5g (one stick) for adults, twice daily (morning and evening) on an empty stomach or between meals.

9.3 Packaging and label

Drugs for clinical trials are manufactured by the clinical trial sponsor, packaged and labeled, and supplied to the managing pharmacist of the clinical trial institution. All production and packaging of test and reference drugs are carried out in GMP facilities according to GMP regulations. The label for clinical investigational drugs is written in accordance with Table 11 of the Regulations on Pharmaceutical Manufacturing and Quality Control (enacted on January 1, 2017). A label containing the following information is produced on the packaging of this drug. However, only the (*) items are indicated on the primary packaging of clinical trial drugs, and all items are indicated on the secondary packaging. In the case of the target allocation number, it is not indicated on the primary packaging . Document the relevant information and keep it .

- Subject allocation number
- Reference code *
- Name of investigational drug*
- Expiration date or re-inspection date
- Batch number or code number *
- Storage conditions
- How to take (take)
- **Name, address, and phone number of the person who has received approval for the clinical trial plan ***
- Keep out of reach of children.
- **Cannot be used for purposes other than clinical trials (primary packaging is " for clinical trials ")***

9.4 Management of investigational drugs

Responsibility for the receipt, storage, dispensing, and return of clinical trial drugs lies with the principal investigator and the managing pharmacist of the relevant implementing institution . Clinical investigational drugs are delivered to the managing pharmacist (or principal investigator) of the implementation institution, and the managing pharmacist (or principal investigator) must confirm and sign in writing the receipt and quantity of all supplied clinical investigational drugs and manage them appropriately. . Clinical trial drugs must be secured and stored in a storage location with limited access, ensure that the clinical trial drugs are administered to research subjects in accordance with the clinical trial protocol, and ensure that the quantity and management of the clinical trial drugs provided to each research subject is regulated. Manage records. The monitor must regularly check the inventory maintained by the researcher or the managing pharmacist to confirm receipt and payment of used clinical trial drugs . The managing pharmacist (or principal investigator) must return the empty packaging paper and remaining amount of the clinical trial medicine provided to the research subject after use, and the clinical trial medicine, unused clinical trial medicine, used/unused packaging paper and medicine returned by the research subject. Return it to the principal investigator.

9.5 Concurrent treatment

Acceptable concomitant medications and treatments

Drug treatment for fatigue (herbal medicine, Prohibit the use of herbal medicines (dry food , etc.) and herbal medicines containing the medicinal ingredients of the test drug. also Limit participation in medical treatment such as cognitive behavioral therapy or counseling. However, exercise to improve general health is permitted.

10 . Research period

3 years after IRB approval

1 1 . Research method

11.1 Research design

Patients will undergo a screening process to determine their eligibility after receiving a detailed explanation of the clinical trial and signing the consent form. Eligible patients will be randomly assigned to the treatment group (CV1, Jin Kyungok-go) or the control group (placebo) and will receive the respective intervention for 12 weeks. Each participant will visit the study site monthly during the intervention period and will undergo symptom evaluation and specimen collection (e.g., blood samples) after the 12-week intervention period, at which point the study will conclude. Recruitment will be conducted through internal and external advertisements.

11.2 Dosage and administration of the herbal medicine

The dosage and administration method for the herbal medicine during the study period are as follows:

[Usage/Dosage]

Code name (herbal medicine name)	usage	One-time administration unit (dose)
CV 1 (Jin Kyungok-go)	●/○/●	1 packet (22.5g)
P (placebo)	●/○/●	1 packet (22.5g)

●Take, ○Do not take

[Administration method]

Administer 2 times a day on an empty stomach or between meals as per the specified method.

11.3 Blinding, etc.

At Visit 0, each subject will be assigned a unique screening number by the researcher. This number will begin with CVS1-01 at Kyung Hee University Korean Medicine Hospital and CVS2-01 at Dong-Eui University Korean Medicine Hospital, and will be assigned in ascending order from the smallest available number. Once assigned, the screening number will not be reused. If a subject is not treated at Visit 1 for any reason, the screening number assignment and the reason for non-treatment will be documented in the Screening Log and the case report form (CRF).

Block randomization will be employed, and the block size will not be disclosed to maintain blinding. An independent statistician will generate random numbers using STATA Version 4.2 (StataCorp LLC., Texas), and these numbers will be sent to the labeling site for the IMPs, where they will be labeled and packaged in the order of randomization. This ensures both the researcher and the subjects are blinded. The probability of allocation to the treatment or control group is 1:1.

To ensure allocation concealment, the IMPs packaged according to the random numbers generated by the independent statistician will be delivered to the trial sites. Subjects who meet the inclusion criteria, do not meet the exclusion criteria, and voluntarily consent in writing will be assigned the lowest available random number in the order of their participation. This number will be recorded in the CRF, and the IMP will be dispensed accordingly.

11.4 Observation and inspection items

1 1.4.1 Clinical trial progress schedule

11.4.1.1 Visit 0 (screening visit)

Participants who apply to join this clinical trial will receive an explanation of the study and then undergo the following tests. Screening tests can be repeated once within the screening period and must be completed before assigning a participant number. If a retest is conducted, the suitability for participation is determined based on the retest results, and the investigator should document the reasons for the retest, the basis for the decision, and the final results in as much detail as possible.

① Before enrolling a clinical trial participant, the principal investigator or the person in charge explains the study process and obtains written consent from the participant.
② Screening numbers and random assignment numbers are assigned in the order of written consent completion.
③ Investigate and record whether the participant is involved in other clinical trials (within the last 30 days), demographic information, medical history (including diabetes), COVID-19 information (date of onset, recovery date, vaccine details including date, manufacturer, doses, and side effects), past fatigue-related history, and current fatigue symptoms. Even if the participant is receiving diabetes treatment, they can join the study if their test results are within controlled ranges (HbA1c within normal range).
④ The physical exam covers general condition, nutritional status, skin/mucosa, eyes (excluding vision impairment), ENT, thyroid, lungs, cardiovascular system, abdomen, renal/urinary system, neurological/psychiatric system, spine/extremities/tumors, peripheral circulation, and lymphatic system.
⑤ If possible, confirm and document COVID-19 infection status through the following:
-SMS notifications from the Disease Control and Prevention Agency regarding confirmed cases.
-Medical records and diagnostic certificates from the time of COVID-19 diagnosis and treatment.
-PCR test records from the time of diagnosis.
-Certificates related to COVID-19 diagnosis or release from quarantine issued by public institutions.
⑥ Verify current medications.
⑦ Measure vital signs.

⑧ Conduct an electrocardiogram (ECG) test.

⑨ Collect blood samples for laboratory tests and immune and metabolite analysis (laboratory test results within 28 days of the screening visit can be used):
-Blood chemistry: AST, ALT, γ-GTP, ALP, HbA1c (considered diabetes if abnormal), BUN, creatinine, total bilirubin, glucose.
-Immunology: COVID-19 specific antibody/neutralizing antibody tests, single-cell transcriptome analysis (scRNA seq.) for immune-related gene expression comparison, FACS for immunophenotyping, serum cytokine levels.
-Metabolomics: primary and secondary metabolite profiling.

⑩ Perform a pregnancy test (urine or serum β-hCG) for women of childbearing potential (test results within 28 days of screening can be used).

⑪ Participants follow the investigator's instructions for the following evaluations:
-Fatigue assessment using FSS.
-Evaluation of whether fatigue symptoms have persisted for more than four weeks.

⑫ Confirm inclusion/exclusion criteria.

11.4.1.2 Visit 1 (start of intervention , Week 1)

If all tests and eligibility assessments are completed during the screening visit, Visit 1 can be conducted on the same day to assign participant numbers. If the screening visit and assignment of participant numbers occur on the same day, overlapping evaluations are performed only once.

① Measure vital signs.

② Investigate past and current medical history (changes between Visit 0 and Visit 1).

③ Assign random numbers.

④ Prescribe a four-week supply of the assigned herbal medicine based on the random number.

⑤ Participants follow the investigator's instructions for the following evaluations:
- Assess fatigue severity using a 0-100 VAS.
- Evaluate fatigue using the Chalder Fatigue Scale (ChFS).
- Assess quality of life using EQ-5D-5L.
- Evaluate sleep status using PSQI-K (Korean version of the Pittsburgh Sleep Quality Index).
- Conduct cognitive function tests using K-MOCA (Korean-Montreal Cognitive Assessment).
- Evaluate depression using Beck's Depression Inventory (BDI).

- Perform digit span tests (forward and backward).
- Conduct computerized neurocognitive function tests.
- Perform the Short Physical Performance Battery (SPPB).

11.4.1.3 Visits 2 and 3 (mid-term visit, Week 4 (28 days) ±2 days, Week 8 (56 days) ±2 days)

The following tests are conducted during mid-term visits:

① Measure vital signs.

② After collecting the prescribed herbal medicine from the previous visit, verify the dosage taken.

③ Participants follow the investigator's instructions for the following evaluations:

- Fatigue assessment using FSS.
- Evaluate fatigue using the Chalder Fatigue Scale (ChFS).
- Assess fatigue severity using a 0-100 VAS.

④ Prescribe a four-week supply of the clinical trial medication based on the random number.

⑤ Investigate concomitant medications.

⑥ Confirm any adverse reactions after taking the clinical trial medication.

11.4.1.4 Visit 4 (End of Intervention, Week 12 (84 days) ±2 days)

During Visit 4, the administration of the clinical trial medication is completed, and the following tests are conducted, marking the end of the clinical trial.

① After collecting the prescribed herbal medicine from the previous visit, verify the dosage taken.

② Measure vital signs.

③ Conduct blood tests and ECG:

- Blood chemistry : AST , ALT , BUN, creatinine
- Electrocardiography: EKG

④ Collect blood samples for immune and metabolite tests:

- Immunology: COVID-19 specific antibody/neutralizing antibody tests, single-cell transcriptome analysis (scRNA seq.) for immune-related gene expression comparison, FACS for immunophenotyping, serum cytokine levels.
- Metabolomics: primary and secondary metabolite profiling.I

⑥ Participants follow the investigator's instructions for the following evaluations:

- Fatigue assessment using FSS.
- Evaluate fatigue using the Chalder Fatigue Scale (ChFS).
- Assess fatigue severity using a 0-100 VAS.
- Assess quality of life using EQ-5D-5L.
- Evaluate sleep status using PSQI-K (Korean version of the Pittsburgh Sleep Quality Index).
- Conduct cognitive function tests using K-MOCA (Korean-Montreal Cognitive Assessment).
- Evaluate depression using Beck's Depression Inventory (BDI).
- Perform digit span tests (forward and backward).
- Conduct computerized neurocognitive function tests.
- Perform the Short Physical Performance Battery (SPPB).

⑤ Investigate concomitant medications.

⑥ Confirm any new adverse reactions since the last visit and follow up on previously reported adverse reactions.

⑦ Conclude the study and provide guidance for any necessary follow-up visits.

11.4.1.7 Unscheduled Visit

If a participant visits on a non-scheduled date due to adverse reactions, changes in concomitant medications, early dropout, or the need for medical treatment based on clinical trial measurements, document the related details as thoroughly as possible. Ensure that the planned trial schedule is not altered due to unscheduled visits. Visits to the hospital due to underlying conditions or screenings not related to the primary condition planned for the clinical trial are not considered unscheduled visits.

Unscheduled visits can occur as needed based on participant requests or investigator judgment. During these visits, conduct all tests corresponding to Visit 4 as much as possible, but do not impose strict restrictions, allowing for additional appropriate tests based on the investigator's discretion.

11.4.2 Observation items

11.4.2.1 Participation in other clinical trials

To prevent participants from concurrently participating in other clinical trials, establish an identity verification process and confirm their participation in other clinical trials during each visit, including the screening visit. Participants cannot enroll in this study if they have participated in another study within the last 30 days.

11.4.2.2 Demographic survey

Collect information on date of birth, age, gender, height, weight, BMI, highest education level, and occupation. Record the date of birth according to the official solar calendar date on the participant's identification card, and calculate age based on the date of written consent. Participants select their highest education level from the options: below elementary school, elementary school, middle school, high school, or university and above. Occupation is categorized as physical labor, non-physical labor, or other (unemployed, etc.).

11.4.2.3 Investigation of past and present medical history

Investigate and record any clinically significant medical or abnormal conditions that the study subjects have experienced in the past or are currently experiencing. This includes the diagnosis, start date, end date, whether the condition persists at the time of screening, and observations. Additionally, collect information on the date of COVID-19 diagnosis. Special attention is given to conditions that might affect drug intake or absorption, such as swallowing disorders, severe digestive disorders, galactose intolerance, Lapp lactase deficiency, glucose-galactose malabsorption, and other genetic problems. Also investigated are past histories of heart diseases like myocardial infarction or heart failure, allergies to the study drug, liver or kidney diseases, and diabetes.

11.4.2.4 Investigation of fatigue-related medical history

Investigate whether the study subjects have previously experienced or are currently experiencing clinically significant conditions that could cause fatigue, such as cancer, sleep disorders, chronic hepatitis, liver cirrhosis, chronic renal failure, tuberculosis, asthma, or multiple sclerosis.

11.4.2.5 Assessment of current fatigue symptoms

Evaluate whether the study subjects have the following fatigue symptoms, and confirm the presence of fatigue if all criteria are met:[21, 22]

- Persistent fatigue exacerbated by physical or mental activity.
- Difficulties in at least three of the four areas: daily activities, occupational ac tivities, social activities, and leisure activities.
- No diagnosis of physical or mental diseases causing fatigue.
- Non-refreshing sleep.

11.4.2.6 Investigation of COVID-19 diagnosis

To determine the COVID-19 diagnosis, verify the following provided by the subjects: text messages from the Disease Control and Prevention Agency, COVID-19 related diagnosis or opinion documents, hospitalization records, and PCR test records at the time of diagnosis. Collect information on the vaccination status (whether vaccinated, type of vaccine, vaccination dates, and any adverse reactions) and the timing of the COVID-19 diagnosis along with the following data:

-Medical history during COVID-19 diagnosis and treatment.

-Early Warning Score (EWS).

The EWS is extracted from the subject's medical records if available, or collected based on the subject's memory if not. Evaluate EWS using SpO2, supplemental oxygen, heart rate, systolic BP, respiratory rate, body temperature, and CNS level. Classify COVID-19 severity based on the National Early Warning Score (NEWS; mild < 5, severe ≥ 5).

1) EWS assessment date (on the worst symptom day), respiratory rate, oxygen saturation, oxygen supplement, body temperature, systolic blood pressure, heart rate, and consciousness.
2) Calculate and record EWS in the Case Report Form (CRF) according to the provided table.

변수	3	2	1	0	1	2	3
산소포화도	91이하	92-93	94-95	96이상			
산소투여여부	예	예	아니오	아니오			
맥박	40이하		41-50	51-90	91-110	111-130	130이상
수축기혈압	90이하	91-100	101-110	111-219			220이상
호흡수	8이하		9-11	12-20		21-24	25이상
체온	35.0이하		35.1-36.0	36.1-38.0	38.1-39.0	39.1이상	
의식수준				정상			이상

11.4. 2.7 Investigation of current medications

Investigate the current medications the subjects are taking. Record the drug name, usage, dosage, unit, administration route, start date, end date, and whether the medication is ongoing, as well as the purpose of the medication.

11.4.2.8 Measurement of vital signs

Measure blood pressure (systolic and diastolic), body temperature, respiratory rate, and pulse rate. All measurements should be taken with the subject in a seated position after 5 minutes of rest.

11.4.2.9 Electrocardiogram examination

Evaluate for severe heart diseases such as acute myocardial infarction and ventricular fibrillation.

11.4.2.10 Laboratory tests

Conduct laboratory tests to perform a basic examination for other diseases. Specific details can be found under "Other Observation Items" in the "Observation Items and Observation Methods" section.

11.4.2.11 Immunological test and metabolite analysis

1. Sample collection and storage

1.1 Blood sampling

Blood sampling is conducted at the designated clinical trial sample analysis institution within Kyung Hee Medical Center, with consent from the subjects. Collect 25ml per draw (4 x 5ml EDTA tubes, 1 x 5ml general tube). After anonymizing samples, quickly transport them in an ice-filled cold box to ROKIT Genomics for scRNA-seq analysis. Two 5ml EDTA tubes are processed to isolate peripheral mononuclear cells using the RBC lysis method, and the remaining two using centrifugation. Blood in the 5ml general tube is separated into serum. Processed samples are temporarily stored at ROKIT Genomics

Temporary Storage Location: ROKIT Genomics, 12th Floor, Gasan Hi-Heel, 9 Digital-ro 10-gil, Geumcheon-gu, Seoul

Storage Conditions: Peripheral blood mononuclear cells (-190℃, liquid nitrogen), serum (-80℃, ultra-low temperature freezer)

Samples are transported to the Korea Institute of Oriental Medicine for storage according to the human-derived material research consent form. Anonymized personal information collected at Kyung Hee University Oriental Medicine Hospital is provided to the Korea

Institute of Oriental Medicine and used solely for human-derived material analysis research, complying with research security regulations. Disposal of human-derived materials is conducted as per the preservation period in the consent form and according to the medical waste disposal manual of the Disease Control and Prevention Agency.

Storage Location: Korea Institute of Oriental Medicine, Room 201, Gu-Am Building, Infection Disease Research Laboratory, 1672 Yuseong-daero, Yuseong-gu, Daejeon

Storage Conditions: Peripheral blood mononuclear cells (-190℃, liquid nitrogen), serum (-80℃, ultra-low temperature freezer)

Human-derived materials are preserved for the duration specified in the research consent form and are not used for secondary purposes. No separate consent is obtained for secondary usage, and personal information for secondary use is not attached.

1.2 Separation and storage of blood single cells and serum

Separate blood single cells from peripheral venous blood using RBC lysis or density gradient centrifugation. Freeze in cryoprotective medium and store in liquid nitrogen until use. Serum is separated post-clotting (30-60 minutes) and aliquoted into cryovials (0.5ml each, 2-3 cryovials per serum), stored in a freezer box at -80℃ until analysis.

① Immunological test

Immunological test items, specimen collection and storage, transport method, and sample analysis subject and analysis method etc.

1. Observed items for immunological testing:

-Immune gene expression analysis using single-cell RNA sequencing (scRNA-seq)
-Immunophenotyping analysis using flow cytometry for SARS-CoV-2 specific antibodies
-Quantitative analysis of cytokine levels in serum
-Quantitative analysis of SARS-CoV-2 specific antibodies in serum
-Quantitative analysis of SARS-CoV-2 neutralizing antibody titers in serum
-Immunological testing schedule: Immediately before herbal medicine administration, immediately after administration, 12 weeks post-administration, 24 weeks post-administration (total 4 times)

2 . Experiment method

2.1 Single-cell transcriptome analysis using scRNA-seq

Analyze the transcriptome of PBMCs from subjects to assess the distribution and expression of immune cells (B cells, plasma cells, NK cells, CD4 T cells, CD8 T cells, myeloid cells, epithelial cells) and changes in specific immune cells' transcriptome expression (cytokine expression, transcription factors, signal pathways) before and after herbal medicine administration. Sample transport and data analysis for scRNA-seq are conducted by ROKIT Genomics. Create scRNA-seq libraries using the Chromium Single Cell 3′ Library & Gel Bead Kit v3 (10×Genomics). Nanoliter-scale droplets isolate thousands of cells, reverse transcribe to cDNA, and assign cell barcoding sequences and UMIs to each cDNA molecule. Analyze sequencing data using the Nextseq 550 or Novaseq 6000 platform (Illumina). Present changes in immune-related gene expression before and after herbal medicine administration using various plots (Barplot-Significant mRNAs, Scatter plot, Volcano plot, Hierarchical clustering heatmap) and summarize candidate mRNAs. Transfer remaining samples to the Korea Institute of Oriental Medicine for storage post-data analysis. Verify selected significant markers via real-time qPCR in individual samples.

2.2. Immunophenotyping analysis using flow cytometry (FACS)

Thaw single cells preserved at the Korea Institute of Oriental Medicine, identify dead cells using Live/Dead Fixable Cell Stain kit, and compare immune cell distribution and activation markers before and after herbal medicine administration. Stain with fluorochrome-conjugated antibodies (anti-CD3, anti-CD4, anti-CD8, anti-CD19) and analyze via FACS.

2.3 Quantitative analysis of cytokines, SARS-CoV-2 specific antibodies, and neutralizing antibody titers in serum

Quantify pro-inflammatory cytokines (IL-8, IL-1beta, IL-6, IL-10, TNF, and IL-12p70) to evaluate the inflammation response and potential sequelae like organ failure post-severe inflammation.
- Compare cytokine levels before and after herbal medicine administration using Cytometric Bead Array kit (BD Biosciences) and FACS at the Korea Institute of Oriental Medicine.
- Measure SARS-CoV-2 specific antibodies in serum using ELISA and HRP-conjugated secondary antibodies at the Korea Institute of Oriental Medicine.
- Assess SARS-CoV-2 neutralizing antibody titers using SARS-CoV-2 Neutralization Detection Kit (GenScript) and plaque reduction neutralization test at Chungbuk National University's biosafety level 3 facility.

① Metabolite analysis

Metabolites are various products generated during metabolism, influenced by both endogenous processes and external factors (infection, gut microbiota), affecting a wide range of physiological and biochemical processes. Using LC-MS and GC-MS, obtain metabolite profiles to observe metabolic changes before and after herbal medicine administration.

1. Overview of blood metabolomics analysis via mass spectrometry

- Metabolites refer to various products generated during metabolic processes. They include direct metabolic products of organisms as well as those produced by external factors (such as infections and gut microbiota) and have extensive physiological and biochemical impacts. Metabolites are involved in regulating direct mechanisms of action and contribute to homeostasis by modulating bacterial metabolic mechanisms. Therefore, integrating and interpreting information about metabolite mechanisms with existing biological data to propose new analytical approaches is crucial for understanding basic biological functions and diseases. High-resolution mass spectrometers are highly efficient devices for simultaneously analyzing the entire metabolome, often combined with separation methods such as gas chromatography (GC) and liquid chromatography (LC).

2. Items to be observed through metabolite analysis

- The types of metabolites analyzed vary according to LC-MS and GC-MS, as shown in the figure below. In this study, both LC-MS and GC-MS analysis methods are selected to obtain metabolic profiles, including primary and secondary metabolites such as alkaloids and derivatives, benzenoids, homogeneous non-metal compounds, lipids and lipid-like molecules, nucleosides, nucleotides, and analogues, organic acids and derivatives, organic nitrogen compounds, organic oxygen compounds, organohalogen compounds, organoheterocyclic compounds, phenylpropanoids, and polyketides, in the serum. These profiles are used to identify metabolic changes before and after herbal medicine administration.

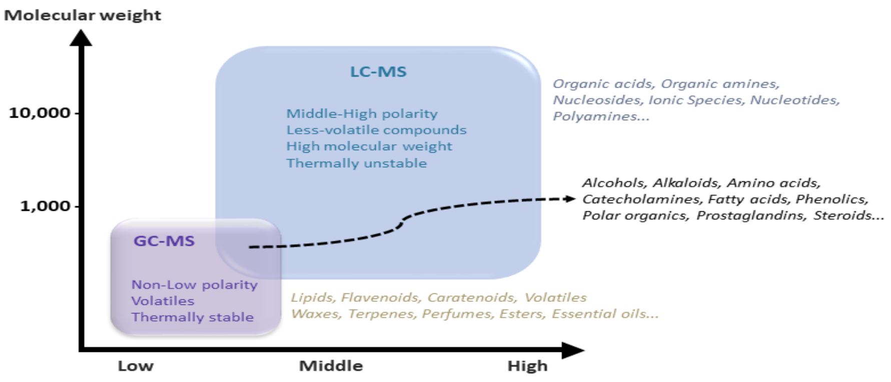

Sample	Analysis method	Performing institution	Analysis purpose
Blood/single cell	scRNA -Seq	ROKIT Genomics	Verify changes in immune-related transcriptome before and after herbal medicine administration
	Real - time qPCR	Korea Institute of Oriental Medicine	Confirm immune-related transcriptome changes before and after herbal medicine administration
	Immunophenotyping using flow cytometry (FACS)	Korea Institute of Oriental Medicine	Verify changes in immune cell distribution before and after herbal medicine administration
Blood/serum	Cytokine analysis (CBA)	Korea Institute of Oriental Medicine	Verify changes in cytokine levels in serum before and after herbal medicine administration
	SARS-CoV-2 specific antibody analysis	Korea Institute of Oriental Medicine	Verify changes in SARS-CoV-2 specific antibody levels before and after herbal medicine administration
	SARS-CoV -2 neutralizing antibody analysis	Korea Institute of Oriental Medicine/ Chungbuk National University	Verify changes in SARS-CoV-2 neutralizing antibody levels before and after herbal medicine administration
	Metabolite analysis	Korea Institute of Oriental Medicine	Verify changes in metabolites before and after herbal medicine administration

<Table> Types of human specimens, analytical methods, performing institution and analysis purpose

11.4.2.12 Assessment of pregnancy and lactation

For women of childbearing age, a β-hCG test is performed using serum or urine tests (sticks). If there are test results within 28 days before herbal medicine administration, this test can be omitted. Women who are menopausal (at least one year of amenorrhea), have undergone a hysterectomy, or have had both ovaries (or fallopian tubes) removed are excluded from the test. The potential for pregnancy and lactation is confirmed verbally.

11.4.2.13 FSS (Fatigue severity scale)

The FSS is an index that evaluates fatigue over the past week. The subject assesses it directly using a questionnaire under the guidance of the principal investigator or person in charge. Before the subject evaluates the questionnaire, the examiner explains the evaluation method. It consists of nine items, each scored from 1 to 7, with higher scores indicating more severe symptoms. The FSS score is calculated as the average of the nine items. Based on guidelines and clinical research results on chronic COVID-19 syndrome, a score above 4 is considered to indicate significant fatigue symptoms.[23, 24]

11.4.2.14 Investigate whether symptoms of fatigue have lasted for more than 4 weeks

The duration of fatigue symptoms is investigated to confirm whether they have persisted for more than four weeks.

11.4.2.15 0-100 Visual analogue scale (VAS) of fatigue

Subjects are asked to mark their average fatigue over the past week. On the 0-100 mm VAS line, 0 means no symptoms, and 100 means very severe symptoms.

11.4.2.16 Chalder Fatigue Scale(ChFS)

The Korean version of the Chalder Fatigue Scale is used for evaluation. Scores are divided into total, physical (items 1-7), and mental health (items 8-11) sub-scales[25] .

11.4.2.17 EQ-5D-5L

EQ-5D classifies health status profiles into five dimensions with five levels: mobility, self-care, usual activities, pain/discomfort, and anxiety/depression. Levels range from 1 (no problem) to 5 (severe problem). Quality of life scores are calculated using the following equation[26] :

$$
\begin{aligned}
\text{Quality weight} = 1 - (&0.096 + 0.046 \times M2 + 0.058 \times M3 + 0.133 \times M4 + 0.251 \times M5 + \\
&0.032 \times S2 + 0.050 \times S3 + 0.078 \times S4 + 0.122 \times S5 + \\
&0.021 \times U2 + 0.051 \times U3 + 0.100 \times U4 + 0.175 \times U5 + \\
&0.042 \times P2 + 0.053 \times P3 + 0.166 \times P4 + 0.207 \times P5 + \\
&0.033 \times A2 + 0.046 \times A3 + 0.102 \times A4 + 0.137 \times A5 + 0.078 \times N4)
\end{aligned}
$$

(N4: 1 if there is any level 4 or above, otherwise 0; M = Mobility, S = Self-care, U = Usual ac tivities, P = Pain/discomfort, A = Anxiety/depression)

11.4.2.18 Korean version of Pittsburgh Sleep Quality Index (PSQI-K)

The PSQI-K consists of 19 self-reported questions and 5 questions for the roommate or bed partner. Only the 19 self-reported questions are used to calculate the global score, which ranges from 0 to 21. Seven component scores are derived from the 19 questions, and the global score is the sum of these component scores.

11.4.2.19 Korean-Montreal cognitive assessment (K- MOCA)

Although the K-WAIS-IV is a good tool for comprehensively assessing cognitive function, it takes more than an hour per evaluation, making it challenging for repeated assessments. Therefore, the K-MOCA, a simpler tool that is more sensitive to cognitive decline than the MMSE, is used. K-MOCA includes visuospatial ability, executive function (3 points), language ability - vocabulary (3 points), language ability - sentence construction (3 points), attention (6 points), calculation (2 points), and orientation (6 points). Memory is included but not scored. The total score is 30 points, with a score of 23 or higher considered normal cognitive function.[30] .

11.4.2.20 Becks' depression inventory (BDI)

This inventory assesses the presence and severity of depressive symptoms following COVID-19 infection or recovery. It consists of 21 items covering cognitive, emotional, motivational, and physical symptoms of depression, with a total score range of 0-63. Higher scores indicate more severe depressive symptoms.[31] [32] .

11.4.2.21 Digit Span Test (Forward and Backward)

The Wechsler intelligence scale, widely used in clinical settings, includes the digit span subtest, which is a simple tool to assess cognitive decline, short-term memory, and working memory. In the digit span test, the examiner reads a series of numbers, and the subject repeats them in the same order (DF) or in reverse order (DB). DF ranges from 3 to 9 digits, and DB ranges from 2 to 8 digits. The test is stopped if the subject fails twice consecutively. The score is the highest number of digits correctly repeated, with separate scores for DF and DB. High scores indicate good short-term memory, and a DF-DB score difference of 5 or more indicates abnormal working memory. [44] .

11.4.2.22 Computerized Neurocognitive Function Test (CNT40)

CNT40 evaluates various neurocognitive functions such as language ability, memory, attention, planning ability, and motor skills through 17 tests using computerized devices. It

assesses cognitive decline in chronic COVID-19 syndrome patients. This study uses CNT40, developed and clinically used in Korea, analyzing five items related to attention and higher cognitive functions: [33, 34].

- Auditory C.P.T (Continuous Performance Test): Measures the subject's accurate response to auditory stimuli.
- Auditory Controlled C.P.T: Measures the subject's accurate response to the same continuous auditory stimuli.
- Visual C.P.T: Measures the subject's accurate response to visual stimuli.
- Visual Controlled C.P.T: Measures the subject's accurate response to the same continuous visual stimuli.
- Trail Making Test: Measures the time taken to connect numbers and letters in sequence.

CNT 40 (MaxMedica Co., Ltd.) equipment	Example of test results
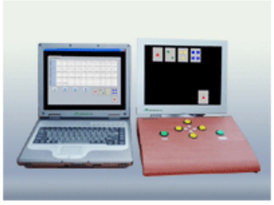	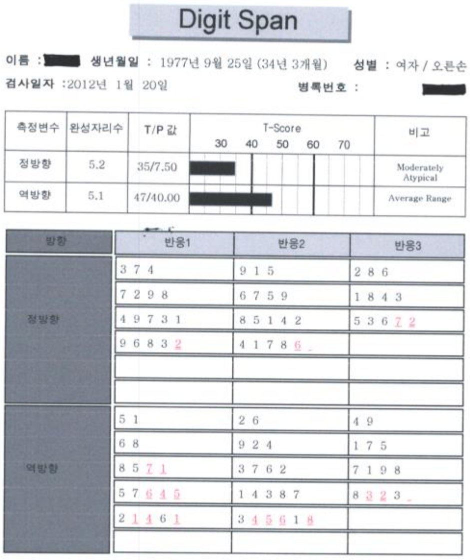

Digit Span

이름 : [redacted] 생년월일 : 1977년 9월 25일 (34년 3개월) 성별 : 여자 / 오른손
검사일자 :2012년 1월 20일 병록번호 : [redacted]

측정변수	완성자리수	T/P 값	T-Score 30 40 50 60 70	비고
정방향	5.2	35/7.50		Moderately Atypical
역방향	5.1	47/40.00		Average Range

방향	반응1	반응2	반응3
정방향	3 7 4	9 1 5	2 8 6
	7 2 9 8	6 7 5 9	1 8 4 3
	4 9 7 3 1	8 5 1 4 2	5 3 6 7 2
	9 6 8 3 2	4 1 7 8 6	
역방향	5 1	2 6	4 9
	6 8	9 2 4	1 7 5
	8 5 7 1	3 7 6 2	7 1 9 8
	5 7 6 4 5	1 4 3 8 7	8 3 2 3
	2 1 4 6 1	3 4 5 6 1 8	

11.4.2.23 Short Physical Performance Battery (SPPB)

The Short Physical Performance Battery (SPPB) is a test that assesses the physical function of elderly individuals or those with post-illness frailty. It comprises three tests: static balance test, gait speed test, and chair stand test. It is administered to evaluate the decline and recovery of physical function in patients with chronic COVID-19 syndrome. The specific methods are as follows:

Static Balance Test: This test evaluates balance in three postures: side-by-side, semi-tandem, and tandem. The subject is required to maintain each posture without losing balance for 10 seconds. The maximum score is 4 points. Successfully maintaining the side-by-side and semi-tandem postures earns 1 point each. For the tandem posture, maintaining it for 10 seconds earns 2 points, for 3-9.99 seconds earns 1 point, and for less than 3 seconds earns 0 points.

Gait Speed Test: This test measures the time taken to walk a distance of 4 meters. The maximum score is 4 points. Completing the walk in less than 4.82 seconds earns 4 points, 4.82-6.20 seconds earns 3 points, 6.21-8.70 seconds earns 2 points, more than 8.70 seconds earns 1 point, and inability to walk earns 0 points.

Chair Stand Test: This test measures the time taken to stand up from a chair five times using only the leg muscles, without the aid of arms. The maximum score is 4 points. Completing the task in less than 11.20 seconds earns 4 points, 11.20-13.69 seconds earns 3 points, 13.70-16.69 seconds earns 2 points, more than 16.70 seconds earns 1 point, and inability to complete five stands or taking more than 60 seconds earns 0 points.

In this study, the Andante Fit system is used for precise measurement. The Andante Fit consists of a gait speed measurement device, a chair stand measurement device, and a static balance measurement device. The system takes 2-3 minutes to complete the measurements, and scores for each item and the total score are automatically generated upon completion. [35] .

Composition of Andante Fit	Example of test results

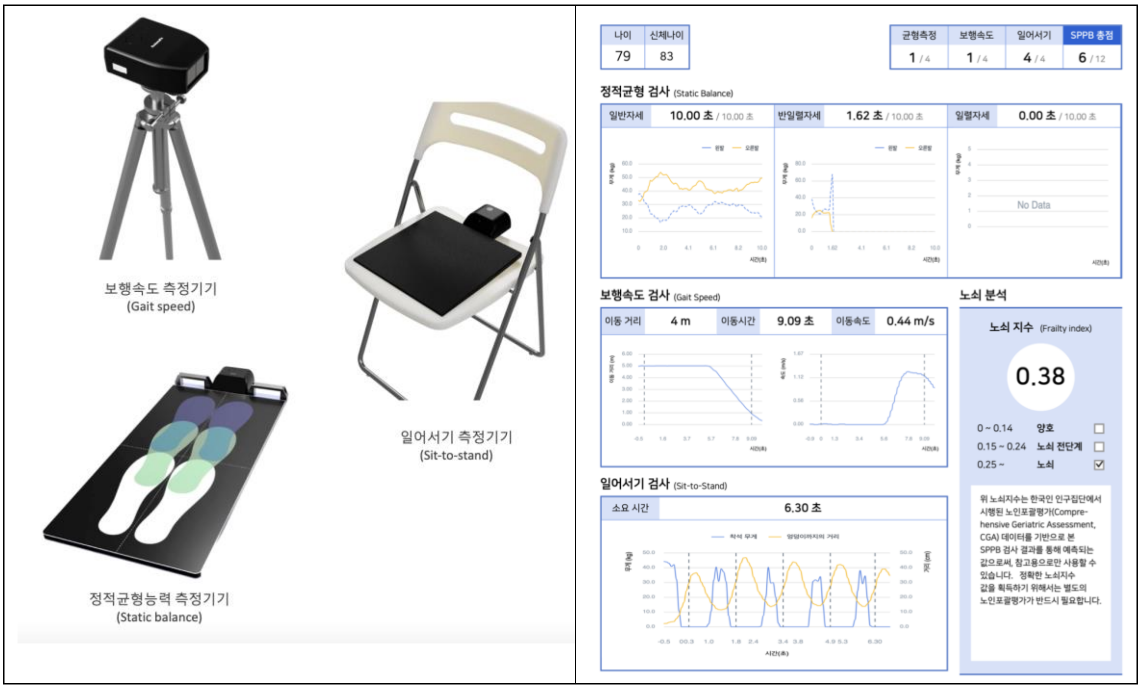

11.4.2.24 Medication compliance

The number of returned and taken medications will be investigated and recorded at each visit. If the number of returned medications and taken medications does not match the prescribed amount, the reason for the discrepancy should be recorded in the case report form. Medication adherence is evaluated as follows:

$$\text{Medication compliance (\%)} = \frac{\text{Number of medications actually taken}}{\text{Number of medications prescribed during the period*}} \times 100$$

* The number of medications prescribed during the period refers to the number of medications that should be taken from the initial administration to the visit immediately prior to returning the medications.

The final medication adherence is evaluated at Visit 4, combining the sum of the herbal preparations actually taken and the clinical trial medications that should have been taken during Visits 2, 3, and 4.

11.4.2.25 Feasibility evaluation

To evaluate the feasibility of the study design, data will be collected on the recruitment rate (overall and by institution), dropout rate (overall and by institution), and reasons for dropout during the study period.

11.4.2.26 Adverse event monitoring

Information about adverse events occurring in study participants should be reported spontaneously by the participants or their proxies and confirmed through interviews and medical examinations by the principal investigator or research staff. The investigation of adverse events includes the date of onset and resolution, severity and outcome of the adverse event, actions taken, the causal relationship with the administration of herbal preparations, and whether and how the adverse event was treated.

- Monitoring subjective and objective symptoms

The degree of adverse event manifestation through subjective and objective symptoms will be investigated during the investigator's medical examinations. The manifestation degree will be recorded according to the evaluation criteria, and the causal relationship with the medication and herbal preparations will also be assessed.

- Monitoring laboratory tests, vital signs, and other measurements

Clinically significant abnormal changes in laboratory tests, vital signs, and other measurements will be followed up until symptoms improve. Additionally, any factors that might have interfered with the test contents during the examination will be recorded through interviews.

12. Precautions for use of investigational medicinal products

12.2 CV1 (Jin Kyungok-go) and P (placebo)

**1. not take this medication if you:**<br>an infant under 3 months of age (newborns, infants).<br>**2. Consult a doctor, herbal doctor, dentist, pharmacist, or herbal pharmacist before taking this medication if you:**<br>1) Are currently taking other medications.<br>2) Are elderly (Generally, elderly individuals have diminished physiological functions and should take care to reduce dosage accordingly).<br>3) Have a significantly weakened stomach (may cause loss of appetite, stomach discomfort, nausea, vomiting, diarrhea, etc.).<br>4) Have symptoms of loss of appetite, nausea, or vomiting (symptoms may worsen).<br>5) Are an infant under 1 year of age (newborns, infants).<br>**3. Discontinue this medication immediately and consult a doctor, herbal doctor, dentist, pharmacist, or herbal pharmacist if:**

bring this information leaflet when consulting.

1) You experience any of the following symptoms after taking this medication:

(1) Skin: Rash, hives, etc.

(2) Digestive system: loss of appetite, stomach discomfort, nausea, vomiting, diarrhea, etc.

2) Your symptoms do not improve after several days of taking the medication.

4. Other precautions while taking this medication:

1) Follow the prescribed dosage and administration instructions carefully.

2) Be cautious of overlapping ingredients when taking with other herbal (medicinal) preparations.

3) When giving this medication to children, ensure it is taken under the guidance and supervision of a guardian.

4) Do not insert a spoon that has been soaked in hot or cold water into the medication bottle (for extract preparations).

5) When reusing a medication spoon, clean it thoroughly without any moisture before using it again (for extract preparations).

5. Storage precautions

1) Store in a cool, dry place away from direct sunlight (ensure it is tightly sealed after use).

2) Keep out of reach of children.

3) Do not transfer the medication to another container, as this may lead to misuse or deterioration in quality. Always keep it in its original container and ensure it is tightly closed.

1 3. Criteria for discontinuation and withdrawal of subjects, and termination of the clinical trial

The participation status of all subjects in this clinical trial will be recorded. If the administration of the investigational medicinal product or observation is discontinued, the reason will be documented. The conditions under which a subject may discontinue participation are as follows:

① In case of acute reactions (allergy, hypersensitivity, etc.) to the herbal preparation.

② If it is found that a subject does not meet the inclusion/exclusion criteria.

③ If unforeseen diseases or accidents occur making administration and observation impossible.
④ If "serious adverse reactions" or "severe adverse drug reactions" occur, making continued administration and observation inappropriate.
⑤ If the subject becomes pregnant.
⑥ If the subject or their proxy requests to discontinue participation (withdrawal of consent).
⑦ Any other reasons deemed appropriate by the researcher for discontinuing the trial.

The termination criteria for this clinical trial are as follows.

The clinical trial will be terminated when the target number of subjects is enrolled, and data integrity is ensured. The principal investigator will record the termination date and conduct data analysis once the follow-up for the last subject is completed. For individual subjects, the clinical trial will conclude upon completion of planned visits and evaluations, and the processes specified in the protocol.

14. Statistical Analysis Methods

14.1 General principles of result analysis

The primary analysis for efficacy evaluation will use the Full Analysis Set (FAS). Safety evaluations will be conducted using the Safety Set. All statistical tests will be two-sided with a significance level of 5%. For efficacy evaluation, missing data in the FAS will be handled using the Last Observation Carried Forward (LOCF) method for statistical analysis, while all other data will be analyzed as originally recorded.

Safety Set (SS): A group of subjects who have received the investigational medicinal product at least once and have undergone at least one safety-related follow-up.
Full Analysis Set (FAS): A group of subjects who, in accordance with the ITT principle, have received the investigational medicinal product and have at least one measurement of the primary efficacy variable.

For analyses other than efficacy evaluation, such as feasibility evaluation (e.g., recruitment rate, dropout rate, reasons for dropout), the recruitment rate will be calculated as the number of enrolled subjects divided by the total number of screened subjects (%). Dropout rates and

reasons will be analyzed descriptively for both the entire study and each herbal preparation group.

To compare differences between groups for efficacy variables, ANCOVA will be conducted with the assigned group as the factor and the baseline value as the covariate (two-sided $p < 0.05$). If assumptions of ANCOVA (normality, homoscedasticity) are violated or outliers are observed, Ranked ANCOVA will be performed. For categorical data, frequencies and percentages will be calculated, and comparisons between groups will be made using the Chi-square test or Fisher's exact test (if expected frequency is ≤ 5). Statistical analyses will be conducted using R-software (latest version) or jamovi software (latest version).

14.2 Demographic baseline data

To test for statistical differences between the two groups in baseline demographic data, FSS scores, and 0-100 VAS scores for symptoms, the following steps will be taken:
Continuous data: mean, standard deviation, median, minimum, and maximum will be calculated, and comparisons will be made using the independent two-sample t-test or Wilcoxon rank-sum test.
Categorical data: frequencies and percentages will be calculated, and comparisons will be made using the Chi-square test or Fisher's exact test (if expected frequency is ≤ 5).
To ensure comparability between the two groups, the FSS score, ChFS score and subscale scores, EQ-5D-5L score, PSQI-K Global score, K-MOCA score, BDI score, DF score, DB score, DF-DB score, K-BNT-15 score, computerized neurocognitive test scores, and SPPB test scores will be calculated for each group and compared using the independent two-sample t-test or Wilcoxon rank-sum test.

14.3 Analysis of efficacy variables

14.3.1 Primary efficacy variable

FSS score after 12 weeks of intervention

14.3.2 Secondary efficacy variables

1) Final medication adherence
2) Differences in final medication adherence between groups
3) FSS scores at visit 2 and visit 3
4) ChFS scores and subscale scores at visits 2, 3, and 4
5) EQ-5D-5L scores at visit 4
6) PSQI-K Global score at visit 4
7) K-MOCA total and domain scores at visit 4

8) BDI scores at visit 4
9) DF score, DB score, and DF-DB score at visit 4
10) Computerized neurocognitive test scores at visit 4
11) SPPB test scores for walking speed, sit-to-stand test, balance test, and total score at visi t 4
12) Analysis of recruitment rate, dropout rate, and reasons for dropout for feasibility evalua tion
13) Treatment success rate in fatigue symptoms after 12 weeks of intervention compared between the treatment and control groups

14.4 Analysis of safety variables

14.4.1 Adverse events

Adverse events (AEs) are defined as any unfavorable symptoms, signs, or diseases occurring in subjects administered the investigational medicinal product. The number of subjects experiencing all AEs, adverse drug reactions (ADRs), serious adverse events (SAEs), and serious adverse drug reactions (SADRs), along with their incidence rates and 95% confidence intervals, will be presented for each group. Differences between groups will be tested using the Chi-square test or Fisher's exact test. All AEs will be coded using the Medical Dictionary for Regulatory Activities (MedDRA) and presented by system organ class (SOC) and preferred term (PT).

14.4.2 Laboratory tests, vital signs

Vital signs and quantitative laboratory test results, including heart rate variability, will be presented as descriptive statistics before and after administration for each herbal preparation group. Changes will be compared between groups using the independent two-sample t-test or Wilcoxon rank-sum test. Within-group changes will be tested using the paired t-test or Wilcoxon signed-rank test. Additionally, laboratory test results before and after administration will be categorized as normal (including clinically insignificant abnormal) or clinically significant abnormal changes. Changes within groups will be tested using McNemar’s test (or McNemar’s exact test).

15. Criteria, methods, and interpretation of efficacy evaluation

15.1 Evaluation items and methods

15.1.1 Primary evaluation variable

1) FSS score after 12 weeks of intervention

FSS scores will be compared between groups after taking 12 weeks of intervention.

15.1.2 Secondary evaluation variables

1) Final medication adherence
: The mean, standard deviation, median, minimum, and maximum of the final medication adherence (%) for all subjects after 12 weeks (visit 4 or end of the study) will be presented.

2) Differences in final medication adherence between groups
: The mean, standard deviation, median, minimum, and maximum of the final medication adherence (%) will be calculated and compared between the two groups after 12 weeks of intervention (visit 4 or end of the study).

3) FSS scores at visits 2 and 3
: The FSS scores at each visit will be compared between groups.

4) ChFS scores and subscale scores at visits 2, 3, and 4
: The total score and the subscale scores for physical (items 1-7) and mental health (items 8-11) will be recorded. The total scores and subscale scores at each visit will be calculated and compared between the groups.

5) EQ-5D-5L scores at visits 2, 3, and 4
: The EQ-5D-5L scores will be calculated, and the scores at each visit will be compared between the groups.

6) PSQI-K global scores at visits 2, 3, and 4
: The scores for the 7 components from the PSQI-K questionnaire will be calculated and summed to determine the Global score. The Global scores at each visit will be compared between the groups.

7) K-MOCA total and domain scores at visit 4
: The scores for the 7 domains will be calculated and summed to determine the total score. The total scores will be compared between the groups at visit 4.

8) BDI score at visit 4
: The scores for the 4 domains will be summed to determine the total score, which will be compared between the groups at visit 4.

9) DF score, DB score, DF-DB scores at visit 4
: These scores will be compared between the groups at visit 4.

10) Computerized neurocognitive test scores at visit 4
: The scores for the 5 computerized neurocognitive tests will be compared between the groups at visit 4.

11) SPPB test scores for walking Speed, sit-to-stand test, balance test, and total score at visit 4
: The scores for these 3 tests and the total score will be compared between the groups at visit 4.

12) Analysis of recruitment rate, enrollment rate, dropout rate, and reasons for dropout for feasibility evaluation
: The recruitment rate will be calculated as the number of enrolled subjects divided by the total number of screened subjects (%). The enrollment rate will be calculated as the total number of subjects divided by the total number of screened subjects. The dropout rates and reasons for dropout will be analyzed for the entire study and for each treatment group, and the results will be compared.

13) Final treatment success rate
: Based on existing data regarding the minimal clinically important difference for fatigue, treatment success is defined as a change of 15 points or more on the 0-100 VAS for fatigue symptoms from pre-treatment (visit 1) to post-treatment (visit 4). The frequency and proportion of subjects achieving treatment success in each group will be presented [36] .

1 6. Methods, criteria, and interpretation for safety evaluation including adverse reactions

16.1 Evaluation methods

Adverse Event (AE) refers to any harmful and unintended sign (including abnormal laboratory results), symptom, or disease that occurs in a study participant who has been administered the herbal preparation, regardless of a causal relationship with the herbal preparation.
Adverse Drug Reaction (ADR) refers to any harmful and unintended reaction at any dose of the herbal preparation, where a causal relationship with the herbal preparation cannot be ruled out. In this study, all adverse events except those assessed as "unrelated" will be classified as ADRs.

The Case Report Form will document the symptoms and signs of adverse reactions, their duration (start and end dates), severity, causal relationship with the herbal preparation, actions taken regarding the adverse reaction, and outcomes comprehensively.

The safety evaluation will include:
- Adverse events, vital signs, laboratory tests, heart rate variability tests

16.2 Evaluation criteria

(1) Severity of adverse events

The severity of adverse events (Intensity) will be evaluated as follows:

severity	explanation
Mild	Minimal interference with normal daily activities (function) and requires no special treatment.
Moderate	Causes discomfort and interferes with normal daily activities (function). The participant can continue the trial but may require treatment.
Severe	Causes significant discomfort, making normal daily activities (function) impossible, requiring treatment or hospitalization.

(2) Causal relationship with the administered herbal medicine preparations

The relationship with the herbal preparation will be classified by the investigator according to the criteria in Annex 77 of the Rules on Safety of Drugs and Medical Devices, and the investigator’s opinion will also be recorded.

Causal Relationship	Judgement Criteria
Clear Relationship	• Evidence of administration of the drug and a plausible temporal sequence of adverse event onset.<br>• The adverse event is more likely explained by drug administration than by any other cause.<br>• The adverse event disappears upon discontinuation of the drug.<br>• Positive re-challenge (if feasible).<br>• Consistency with known information about the drug or its class.
Probable Relationship	• Evidence of administration of the drug and a plausible temporal sequence of adverse event onset.<br>• The adverse event is more likely explained by drug administration than by any other cause.<br>• The adverse event disappears upon discontinuation of the drug.
Possible Relationship	• Evidence of administration of the drug and a plausible temporal sequence of adverse event onset.<br>• The drug administration is as likely as other causes to explain the adverse event.<br>• The adverse event disappears upon discontinuation of the drug (if conducted).
Unlikely Relationship	• Evidence of administration of the drug, but other causes are more likely to explain the adverse event.<br>• The adverse event does not disappear upon discontinuation of the drug (if conducted) or results are ambiguous.
No Relationship	• The participant did not receive the drug, or the temporal sequence of drug administration and adverse event onset is not plausible.<br>• There is a clear alternative cause for the adverse event.
Not Assessable	• Incomplete information prevents assessing the relationship with the drug.

(3) Actions taken in response to adverse events

The actions taken in response to the adverse event will be categorized as follows:

- Drug withdrawn
- Dose reduced

- Dose not changed
- Unknown
- Not applicable

(4) Outcomes of adverse events

The outcomes of adverse events that occur during the study will be classified as:

- Recovered/Resolved
- Recovering/Resolving
- Not recovered/Not resolved
- Recovered with Sequelae/Resolved with Sequelae
- Death
- Unknown

(5) Predictable adverse reactions to the investigational drug

- Skin: Rash, urticaria, eczema, dermatitis exacerbation.
- Gastrointestinal: Loss of appetite, gastric discomfort, nausea, vomiting, diarrhea.

(6) Identification of major adverse reaction symptoms

- Skin: subjective sensations, physical examination
- Gastrointestinal: subjective sensations

16.3 Reporting adverse events

The principal investigator will educate the study personnel and participants or their guardians about all potential adverse reactions that may occur after the administration of the investigational drug and instruct them to report any occurrences.

The Case Report Form will document and retain records of all symptoms, their duration, severity, treatment, medication, progress, and causal relationship as per clinical trial management standards.

16.3.1 Serious adverse events(SAE) and Serious adverse drug reactions(SADR)

SAEs and SADRs refer to adverse events or drug reactions occurring at any dose of the herbal preparation that result in:

① Death or life-threatening condition.

② Necessity for hospitalization or extension of hospitalization.

③ Permanent or significant disability or functional impairment.

④ Congenital anomalies or birth defects.

⑤ Medically significant conditions such as drug dependence, abuse, or blood disorders.

Any other condition that significantly impacts the participant's well-being or health, in the investigator's and related experts' medical judgment, will be considered a serious adverse reaction and appropriate actions will be taken.

The principal investigator will report all newly occurring serious adverse events during the clinical trial to the IRB following the institution's regulations. Continuous monitoring and additional reporting to the clinical trial support organization and IRB will be conducted. The principal investigator will notify the clinical trial sponsor, the Korea Institute of Oriental Medicine, and the monitoring personnel immediately to ensure appropriate actions are taken. When reporting a death, the principal investigator must submit additional information such as an autopsy report (if performed) and final medical records to the clinical trial support organization and the review board.

The following are not considered serious adverse events for "hospitalization or extended hospitalization":

- Diagnosis purposes
- Cosmetic surgery
- Precautionary examination
- Minor admissions (e.g., elective admission for social reasons)
- Long-term care or rehabilitation
- Relocation to a facility due to inability to continue home care
- Efficacy and safety assessments related to the study
- Scheduled treatment of the disease under study
- Pre-planned admissions without patient condition deterioration

16.3.2 Unexpected adverse drug reactions

Unexpected ADRs differ in nature or severity from the available drug information (e.g., package insert).

When "Suspected Unexpected Serious Adverse Drug Reactions (SUSAR)" occur, the IRB will be notified to decide whether to continue or suspend the trial, and the clinical trial support organization will report to the Ministry of Food and Drug Safety within the specified period.

The principal investigator will conduct the study in accordance with the Declaration of Helsinki.

16.4 Follow-up of adverse events

The principal investigator will follow up on all serious adverse events until resolution, stabilization, or loss to follow-up. In cases of significant changes, such as discharge from hospitalization, a follow-up report will be documented and submitted to the clinical trial support organization via email or fax.

17. Compensation regulations for subjects

If physical injury (including death) occurs to the study participant due to the research, the clinical trial support organization is legally responsible for compensation in accordance with compensation regulations. Compensation will also be provided for injuries occurring during corrective treatments related to adverse reactions.

18. Informed consent of participants

The researcher must explain all aspects of the study, including the effects, adverse reactions, and safety of the investigational drug, to the participant and obtain voluntary signed consent before proceeding with the study.

19. Actions in case of adverse events

Participants will receive necessary examinations and treatment immediately from the researcher in case of adverse events. The study will be immediately suspended in case of serious ADRs, and appropriate actions will be taken according to the principles for responding to adverse events.

Participants with adverse events will be continuously monitored via phone or outpatient visits until resolution or stabilization, or until follow-up is no longer possible. The duration of monitoring will be documented in the case report form.

During the clinical trial, responsibilities in the event of a "serious adverse event" are as follows:

1) Principal investigator responsibilities

The principal investigator must immediately report any serious adverse event to the clinical trial

support organization or monitoring personnel within 24 hours of recognition and report to the IRB according to its regulations. Detailed follow-up reports must be provided within the same timeframe. Unexpected serious ADRs must be promptly reported to the clinical trial support organization and the IRB. Additional information, such as autopsy reports (if applicable) and final medical records, must be submitted when reporting deaths.

2) Study Personnel Responsibilities

Study personnel must immediately report any serious adverse event to the principal investigator and the clinical trial support organization.

3) IRB responsibilities

The IRB must take necessary actions, such as ordering the suspension of all or part of the study, if unexpected serious ADRs or new information that may negatively affect the participant's safety or the study's conduct arises.

4) Clinical trial support organization responsibilities

The clinical trial support organization must report all serious and unexpected ADRs to other relevant researchers, the Ministry of Food and Drug Safety, and, if necessary, the IRB within 15 days. If the event results in death or is life-threatening, reporting must occur within 7 days, with detailed information provided within 8 days of the initial report. When reporting unexpected serious ADRs to the Ministry of Food and Drug Safety, the clinical trial support organization must include the ADR report and a summary in the CIOMS-Ⅰ format. Any additional information must be reported periodically until the adverse reaction is resolved or follow-up is no longer possible. Researchers must actively cooperate in providing data and information for these reports.

20. Measures for protecting the safety of research subjects

20.1 Clinical trial institution

The head of the institution must ensure that each clinical trial phase is equipped with the necessary facilities and professional staff, thoroughly preparing to conduct the trial properly.

20.2 Principal investigator

The principal investigator must secure the necessary personnel, equipment, and facilities to conduct the research appropriately and safely throughout the study period.

20.3 Research staff

Research staff must fully understand the research protocol, information about the herbal preparations used in the study, and their duties and responsibilities related to the research.

20.4 Approval and modification of the research protocol

When seeking approval for the research protocol or making changes to an approved study, the protocol or amended protocol must be approved by the Institutional Review Board (IRB) for each stage of the study.

20.5 Familiarity with the research protocol

This clinical trial is prepared with the rights and welfare of the subjects in mind, based on the Declaration of Helsinki. Investigators and staff must thoroughly analyze and understand the protocol and proactively address any issues related to the subjects.

20.6 Accurate selection of research subjects

Prior to the clinical trial, a thorough evaluation is conducted to assess the suitability of subjects through sufficient interviews and examinations.

20.7 Actions in case of adverse reactions

In case of adverse reactions, immediate management is required to ensure subjects receive necessary tests and treatment from the investigator. If serious drug reactions occur, the trial must be stopped, and swift, appropriate action must be taken according to the adverse event management guidelines.

Subjects experiencing adverse reactions must be continuously monitored through calls from the investigator or outpatient visits until the reaction resolves, stabilizes, or follow-up fails. The duration must be recorded in the case report form.

21. Other necessary measures for safe and scientific conduct of clinical trials

21.1 Monitoring

Monitoring is conducted to protect the rights and welfare of research subjects, ensure that reported trial data is accurate, complete, and verifiable against source documents, and confirm that the study is conducted according to the approved protocol, Good Clinical Practice (GCP), and relevant regulations.

Monitoring will involve regular visits and calls by monitoring personnel delegated by the clinical trial support institution or the clinical trial institution. During visits, monitors will verify original records, data storage (research files), and other relevant documents.

Monitors will observe the research process and consult with the principal investigator if issues arise. The appropriate timing of these visits will be mutually agreed upon by the investigator and the monitoring personnel. Researchers must allow monitors access to subject source documents (hospital or personal charts, lab result records, appointment records, etc.) as defined by GCP.

21.2 Progress review

The principal investigator must regularly report adverse reactions, trial progress, and results to the clinical trial support institution. The institution may conduct inspections to ensure the reliability of the collected data according to the protocol, standard operating procedures (SOPs), and relevant regulations.

21.3 Data storage

The principal investigator is obligated to maintain and provide basic research documents, which include all work logs, source documents, monitoring records, scheduled agreements, correspondence between the clinical trial support institution and the investigator, and regulatory documents (e.g., protocols and amendments, IRB correspondence, signed informed consent forms, case report forms, clinical trial drug receipt records, use and return records, lab results). Source documents include all observation records, clinical activity records, and reports and records necessary for trial evaluation and reconstruction.

Source documents and other research-related documents must always be stored at the research institution. The investigator must retain all records until the final report is completed, after which they will be transferred to the responsible custodian of the research institution. The institution's head and the clinical trial support institution must retain the protocol, documents, approvals, and all other materials for three years post-trial completion. Expired records must be shredded to prevent content leakage, and personal information must be

destroyed according to Article 16 of the Personal Information Protection Act Enforcement Decree.

21.4 Confidentiality of research subjects

Records identifying research subjects will be kept confidential. Even if research results are published, subject identities will remain confidential. Specific details are as follows:

The clinical trial support institution, monitors, and inspectors related to this study may review subjects' records for monitoring, inspection, and progress management purposes. By signing this protocol, the investigator acknowledges that monitors and inspectors may review or copy subjects' charts and case report forms to verify data, in accordance with domestic laws and ethical standards. These documents must be kept confidential, with appropriate facilities and management standards for confidentiality. All clinical trial documents, such as case report forms, must be recorded and identified with codes rather than subject names.

21.5 Measures to protecting information on human-derived materials

- Privacy protection and safe handling measures for research data on blood sample donors: For the protection of participants' personal information during sample testing, labels indicating initials, collection date, and sample type (blood sample) will be attached and managed. All data will be managed with assigned numbers, preventing personal information from being leaked.

- Disposal of blood samples: As described in the informed consent for human-derived material research, samples will be immediately disposed of according to the standards and methods under Article 13 of the Waste Management Act after analysis, with procedures followed in the event of abnormal termination of the study.

22. Benefits of clinical trial participation

The costs incurred during the clinical trial (registration, examination, and treatment) will be borne by the investigator. Participants will receive transportation and compensation (a total of 350,000 KRW: 100,000 KRW for 2 visits, 50,000 KRW for 3 visits) when blood is drawn during visits, with 100,000 KRW for visit 4, and 50,000 KRW for other visits (Visits 1, 2, and 3). Non-trial-related medical expenses must be covered by the participants. Transportation and compensation will be provided per visit, post-completion. If participation is discontinued due to dropout or withdrawal, compensation for visits beyond the discontinuation point will not be provided. Payments will be made via bank transfer and may take some time post-visit

completion. Participants assigned to the placebo group will receive a 12-week supply of Kyungok-go after the trial ends.

23. Transport, storage, analysis, and disposal of clinical trials samples

The test results from the subjects' samples collected for this trial will be used solely for this research purpose. Samples will not be stored long-term and will be disposed of according to the analysis institution's SOPs post-trial. Samples or results will not be sold, rented, or provided to independent third parties for other purposes.

24. Policy on report submission and publication

The principal investigator must strive to facilitate the free exchange of relevant scientific information. Presentation or publication of results must align with the principal investigator's plans. The final report will be prepared once all subjects have completed all visits, but interim reports may be drafted upon request from the supporting institution.

25. Protection measures for vulnerable research subjects

Vulnerable subjects, as defined in Annex 4 of the "Regulations on the Safety of Pharmaceuticals, etc.", include those whose voluntary participation decision may be influenced by expectations of benefits or fear of disadvantages from superiors, incurable patients, those in institutions, unemployed, impoverished, emergency patients, minority races, homeless, refugees, minors, and those unable to provide voluntary consent. This trial allows participation from healthcare workers, unemployed, impoverished, and those aged 65 and older. Researchers will not engage in recruitment inducements but will allow participation within the scope of protecting their rights and welfare if they voluntarily wish to participate. To confirm voluntary consent, a statement, "I have voluntarily participated in this clinical trial after hearing the explanation," will be included and signed in the consent form.

26. Recruitment plan for research subjects

Subjects will be recruited by posting notices inside and outside the institution and on online platforms. Recruitment notices will include contact information for research staff or coordinators, and interested subjects will undergo written consent and screening evaluation.

27. Risk/benefit assessment

Potential risks of the study: rare occurrences of hyperaldosteronism, myopathy, liver dysfunction, interstitial pneumonia, skin rash, gastrointestinal issues like anorexia.
Potential benefits of the study: Expected to help improve subjects' symptoms.

Risk/benefit analysis: Considering potential risks and benefits, the benefits are not expected to significantly outweigh the risks, justifying the clinical trial's value.

28. Ethical aspects of clinical trials

28.1 Institutional review board

The IRB must review the protocol and all related documents to ensure ethical principles based on the Declaration of Helsinki are sufficiently reflected. The IRB must also review the validity of participation for subjects in vulnerable situations to ensure their protection.

28.2 Research Director

The principal investigator must conduct the trial at their affiliated institution, ensuring the trial adheres to the protocol, ethical principles of the Declaration of Helsinki, ICH GCP regulations (ICH Topic E6), or relevant laws. Only subjects who voluntarily consent to participate should be included in the trial.

references

1. Chopra, V., et al., *Sixty-Day Outcomes Among Patients Hospitalized With COVID-19.* Annals of Internal Medicine, 2020.
2. O'Mahoney, LL, et al., *The prevalence and long-term health effects of Long Covid among hospitalized and non-hospitalised populations: A systematic review and meta-analysis.* EClinical Medicine, 2023. **55** :p. 101762.
3. Townsend, L., et al., *Persistent fatigue following SARS-CoV-2 infection is common and independent of severity of initial infection.* Plos one, 2020. **15** (11): p. e0240784.
4. Clayton, EW, *Beyond myalgic encephalomyelitis/chronic fatigue syndrome: an IOM report on redefining an illness.* Jama, 2015. **313** (11): p. 1101-1102.
5. Shin, CM, et al., *DA-9701 on gastric motility in patients with Parkinson's disease: a randomized controlled trial.* Parkinsonism & related disorders, 2018. **54** : p. 84-89.
6. Soriano, JB, et al., *A clinical case definition of post-COVID-19 condition by a Delphi consensus.* The Lancet Infectious Diseases, 2022. **22** (4): p. e102-e107.
7. *COVID-19 rapid guideline: managing the long-term effects of COVID-19. Nice. '2022.1.3.*
8. Kim, Y., et al., *Preliminary guidelines for the clinical evaluation and management of long COVID.* Infection & chemotherapy, 2022. **54** (3): p. 566-597.
9. Islam, MF, J. Cotler, and LA Jason, *Post-viral fatigue and COVID-19: lessons from past epidemics.* Fatigue: Biomedicine, Health & Behavior, 2020. **8** (2): p. 61-69.
10. Nam Dong-hyeon , *chronic fatigue syndrome About Bojungikgitang and that frontier Effect : Systematic Literature review* . Journal of Korean Medicine , 2020. **41** (1): p. 93-106.
11. Jiwon Kim , et al., *Chronic fatigue Current status of oriental medicine clinical care About inspection research* . Journal of Consent Physiology and Pathology , 2018. **32** (2): p. 126-133.
12. Yongan Kim , et al., *Gyeongokgoui Anti-fatigue Efficacy* . Journal of Herbal Medicine , 2016. **47** (3): p. 258-263.
13. Joo Hee-cheol , *Gyeongok High School Dosage cardio When exercising blood To recover from fatigue crazy effect* . Chung-ang University Graduate school Department of Physical Education doctoral degree Thesis , 2004.
14. Dong-gun Kim , Won-hyung Park , and Yun-yeop Cha , *Gyeongok High School intake high school soccer player exercise performance Improving and fatigue to recovery crazy effect* . Journal of Consent Physiology and Pathology , 2011. **25** (5): p. 934-944.

15. Jang, S., et al., *Telemedicine and the use of Korean medicine for patients with COVID-19 in South Korea: observational study.* JMIR Public Health and Surveillance, 2021. **7** (1): p. e20236.
16. Song, H.-S., et al., *Anti-inflammatory activity of Kyungok-go on Lipopolysaccharide-Stimulated BV-2 Microglia Cells.* Journal of Korean Medicine, 2022. **43** (4): p. 20-32.
17. Kim, J.-W., et al., *The efficacy, effectiveness, and safety of Kyung-ok-ko: A narrative review.* Medicine, 2022. **101** (45): p. e31311.
18. Pinto, C., et al., *Automated Mechanical Peripheral Stimulation Improves Gait Parameters in Subjects With Parkinson Disease and Freezing of Gait: a Randomized Clinical Trial.* American journal of physical medicine & rehabilitation, 2018. **97** (6): p. 383-389.
19. Zhang, J., YZ Ma, and XM Shen, *Evaluation on the efficacy and safety of Chinese herbal medication Xifeng Dingchan Pill in treating Parkinson's disease: study protocol of a multicenter, open-label, randomized active-controlled trial.* Journal of integrative medicine, 2013. **11** (4): p. 285-290.
20. Bültmann, U., et al., *Measurement of prolonged fatigue in the working population: determination of a cutoff point for the checklist individual strength.* Journal of occupational health psychology, 2000. **5** (4): p. 411.
21. Fukuda, K., et al., *The chronic fatigue syndrome: a comprehensive approach to its definition and study.* Annals of internal medicine, 1994. **121** (12): p. 953-959.
22. Sharpe, M., *A report–chronic fatigue syndrome: guidelines for research.* Journal of the Royal Society of Medicine, 1991. **84** (2): p. 118-121.
23. Korean Society of Infectious Diseases , *Chronic COVID- 19 Syndrome (Long COVID) Treatment Guidelines Spare Recommendation .* 2022.
24. Naik, H., et al., *Evaluating fatigue in patients recovering from COVID-19: validation of the fatigue severity scale and single item screening questions.* Health and Quality of Life Outcomes, 2022. **20** (1): p. 1-9.
25. Ha, H., et al., *Cross-Cultural Validation of the Korean Version of the Chalder Fatigue Scale.* International Journal of Behavioral Medicine, 2018. **25** (3): p. 351-361.
26. *University of Ulsan Industry-academia cooperation group , health related of life quality Measurement tool (EQ-5D) validity Evaluation , Korea Centers for Disease Control and Prevention .*
27. Herdman, M., et al., *Development and preliminary testing of the new five-level version of EQ-5D (EQ-5D-5L).* Quality of life research, 2011. **20** (10): p. 1727-1736.
28. Buysse, DJ, et al., *The Pittsburgh Sleep Quality Index: a new instrument for psychiatric practice and research.* Psychiatry research, 1989. **28** (2): p. 193-213.
29. Teasdale, H., et al., *Safety and efficacy of high definition tDCS for proprioception and balance in Parkinson's disease: a pilot randomized trial.* Movement disorders, 2018. **33** : p. S516-.
30. Seo, J., H. Lee, and M.K. Sunwoo, *Validation of MoCA–MMSE conversion scales in Korean Patients with Cognitive Impairments (4013)* . 2020, AAN Enterprises.

31. Teasdale, HE, et al., *Abstract #31: safety and efficacy of high definition tDCS for proprioception and balance in Parkinson's disease.* Brain stimulation, 2019. **12** (2): p. e11-e12.
32. Youngho Lee and Jongyong Song , *BDI, SDS, MMPI-D scales reliability and To validity About research* . Korean Journal of Clinical Psychology, 1991. **10** (1): p. 98-113.
33. Calabria, M., et al., *Post-COVID-19 fatigue: the contribution of cognitive and neuropsychiatric symptoms.* Journal of neurology, 2022. **269** (8): p. 3990-3999.
34. Koo Shin-sil and Park Jae-guk , *to children with cerebral palsy About Computerized Neurocognitive Test (CNT) Usefulness research* . Research on physical overlapping health disorders (formerly duplication · Education for children with disabilities), 2010. **53** (2): p. 137-155.
35. Jung, HW, et al., *Validation of a multi–sensor-based kiosk for Short Physical Performance Battery.* Journal of the American Geriatrics Society, 2019. **67** (12): p. 2605-2609.
36. Nordin, Å., et al., *Minimal important differences for fatigue patient reported outcome measures—a systematic review.* BMC Medical Research Methodology, 2016. **16** (1): p. 1-16.
