# Supplementary material for: Kyungok-go for fatigue in patients with long COVID: Double-blind, randomized, multicenter, pilot clinical study protocol
Source: PLoS One. 2025 Apr 1;20(4):e0319459. doi: 10.1371/journal.pone.0319459 (PMC11960999; doi:10.1371/journal.pone.0319459)
Supplement: S3 File — (PDF) [file pone.0319459.s003.pdf]

임상시험계획서

만성 코로나 19 증후군(Long COVID) 환자의 피로에 대한 경옥고의 효과 평가 임상시험: 이중맹검, 무작위배정, 다기관, 예비, 연구자 임상시험

Kyungokgo for fatigue in long COIVD patients: double-blind, randomized, a multi-center, pilot clinical study

**Protocol number**	CV2023
**지원기관**	한국한의학연구원
**Version number**	1.2
**Version date**	2023.06.08

목차

임상시험계획서 1

LIST OF ABBREVIATIONS 10

1. 임상시험 명칭과 단계 12

2. 임상시험 실시기관명과 주소 12

3. 임상시험의 책임자 12

4. 임상시험 지원기관 12

5. 임상시험의 배경과 목적 13

6. 대상질환 18

7. 대상자 선정 18

8. 대상질환의 표준 치료 방법 20

9. 임상시험에 사용되는 의약품 21

10. 연구기간 24

11. 연구방법 24

12. 임상시험용의약품의 사용상 주의사항 41

13. 연구대상자의 중지 및 탈락기준, 임상시험 종료기준 42

14. 통계 분석 방법 43

15. 유효성 평가기준, 평가방법 및 해석방법 45

16. 이상반응을 포함한 안전성 평가방법, 평가기준 및 해석방법 47

17. 피해자 보상에 대한 규약 51

18. 연구대상자 동의 51

19. 이상반응 발생 시 조치 51

20. 연구대상자의 안전 보호에 관한 대책 53

21. 기타 임상시험를 안전하고 과학적으로 실시하기 위하여 필요한 사항 54

22. 임상시험 참여에 대한 혜택 56

23. 임상시험용 검체의 이송, 보관, 분석, 폐기 56

24. 보고서 제출 및 출판에 관한 방침 56

25. 취약한 연구대상자에 대한 보호대책 57

26. 연구대상자 모집방안 57

27. 위험/이익 평가 58

28. 임상시험의 윤리적 측면 58

참고문헌 59

Protocol version history

**Protocol ver. 1.0**	**2023.02.10**
**Protocol ver. 1.1**	**2023.05.09**
**Protocol ver. 1.2**	**2023.06.08**

임상시험요약

**임상시험 제목**	만성 코로나 19 증후군(Long COVID) 환자의 피로에 대한 경옥고의 효과 평가 임상시험: 이중맹검, 무작위배정, 다기관, 예비, 연구자임상시험
**임상시험 기관 및 책임자**	경희대학교한방병원 김태훈 교수<br>동의대학교부속한방병원 권찬영 교수
**지원기관**	한국한의학연구원
**임상시험 목적**	본 연구는 피로를 주증상으로 하는 만성 코로나 19 증후군(Long COVID) 환자를 대상으로 육체피로를 효능효과로 시판중인 한약제제(경옥고)의 목표 적응증에 대한 유효성과 안전성을 탐색하고, 후속 확증 임상시험을 위한 시험설계, 평가항목, 평가방법 등에 대한 근거를 마련하기 위해 시행되는 전향적 예비임상시험으로, COVID-19 의 진단 후 최소 12 주가 지났음에도 지속적으로 피로를 호소하는 환자를 경옥고군 또는 위약군으로 무작위배정하고 12 주간 투여 후 허가된 용법, 용량대로 총 12 주간 복용하게 하고, 치료 전후 증상에 대해서 평가하며, 중재의 적용기간 중 복약순응도와 부작용을 관찰하여 기초적인 분석을 실시하는 것을 연구목적으로 한다. 본 연구의 1 차 목적은 만성 코로나 19 증후군 환자의 피로에 대한 12 주 한약제제(경옥고) 복용 후 FSS(Fatigue severity scale)의 변화를 탐색해 보는 것이다. 또한 연구기간동안 대상자의 모집율과 중도탈락율 등 연구설계의 적용가능성(feasibility)을 평가하는 것도 본 연구의 목적이다. 더불어 경옥고의 복용이 COVID-19 면역반응에 미치는 영향 및 피로와 유관한 대사체(metabolites)에 미치는 영향을 평가하는 것을 부수적으로 탐색하는 것을 목적으로 한다.
**대상질환**	피로
**선정 및 제외기준**	**1) 선정 기준**<br>● 만19세 이상 성인으로 COVID-19에 확진된 후 최소 12주를 경과한 자<br>● COVID-19에 확진되기 이전에 경험하지 않았던 피로를 지난 4주이상 지속적으로 경험하는 자<br>● FSS 점수가 4점 이상인 자<br>● 전반적 인지기능에 문제가 없고, 자발적으로 서면동의를 통해 연구 참여에 동의한 자<br>**2) 제외 기준**<br>● 피로를 유발할 수 있는 기질적인 질환(암, 수면장애, 만성 간염, 간경화, 만성 신부전, 결핵,천식,다발성경화증)의 과거력 혹은 현병력이 있는 자<br>● 약물의 복용이나 흡수에 영향을 줄 수 있는 질환(연하장애, 임상적으로 심각한 소화장애, 갈락토오스 불내성(galactose intolerance), Lapp 유당분해효소 결핍증(Lapp lactase deficiency), 포도당-갈락토오스

	흡수장애(glucose-galactose malabsorption) 등의 유전적인 문제 등)이 있는 경우<br>● 조절되지 않는 당뇨환자<br>● 임상시험용 의약품(경옥고)에 대한 알러지 과거력이 있는 경우<br>● 간질환이나 신장질환의 과거력이 있거나 혈액검사상 AST, ALT, BUN, Creatinine이 정상상한치의 3배를 초과하는 경우<br>● 임신 중이거나, 임신가능성 있거나, 수유 중인 여성<br>● 임상시험 참여 전 30일 이내에 다른 연구에 참여한 경우<br>● 임상적으로 유의한 정신과적인 증상이나 의학적인 질환, 검사실 소견 등에 의해 연구 참여가 어렵다고 연구자가 판단한 경우
**목표 대상자수**	본 연구는 예비연구로서 치료군(경옥고투여군) 50명, 대조군(위약투여군) 50명으로 총 100명의 연구대상자를 모집하여 진행한다.
**시험 방법**	연구참여자는 본 임상시험에 대한 충분한 설명을 듣고 동의서에 서명한 다음 연구대상자로서 적합한지 스크리닝 과정을 거친다. 연구대상자로 적합한 참여자는 무작위배정을 통해 치료군(경옥고투여군) 혹은 대조군(위약군)으로 배정된 후 12주간 각 중재를 투여 받게 된다. 각 대상자는 12주의 중재 투여기간 중 한달간격으로 방문하며, 중재 투여가 종료된 후 증상 및 혈액학적 변화, 면역학적 변화를 평가한 후 참여가 종료된다.
**임상시험기간**	임상시험 개시일로부터 3 년
**임상시험용 의약품 및 투여 방법**	코드명(한약제명) / 용법 / 1 회투여단위(용량)<br>CV1(진경옥고) / ●/○/● / 1포(22.5g)<br>●복용, ○복용하지 않음
**유효성 평가변수**	**일차 평가변수**<br>1) 12 주 중재의 복용 후 FSS 점수<br><br>**이차 평가변수**<br>1) 최종 복약 순응도<br>2) 최종 복약 순응도의 군별 차이<br>3) 방문2, 3 시점에서의 FSS 점수<br>4) 방문2, 3, 4 시점에서의 ChFS 점수 및 subscale 점수<br>5) 방문4 시점에서의 EQ-5D-5L 점수<br>6) 방문4 시점에서의 PSQI-K의 Global score점수<br>7) 방문4 시점에서의 K-MOCA 총 점수와 영역별 점수<br>8) 방문4 시점에서의 BDI 점수<br>9) 방문 4 시점에서의 DF 점수, DB 점수, DF-DB 점수<br>10) 방문 4 시점에서의 전산화 신경인지기능 검사 점수

<table>
<tr><td></td><td>11) 방문 4 시점에서의 SPPB 검사의 보행속도 검사, 일어서기 검사, 정적균형 검사, 종합점수
12) Feasibility 평가를 위한 대상자 모집율, 중도탈락율, 중도탈락 이유 등에 대한 분석
13) 피로 증상에 대한 12주 중재의 복용 후 치료군과 대조군 간의 치료성공률</td></tr>
<tr><td>안전성 평가변수</td><td>1)중재의 복용 시 또는 복용 후 발생한 이상 반응에 대한 연구대상자의 호소 증상
2)이학적 검진 상 발견된 이상증상
3)혈액화학적 검사와 심전도 검사</td></tr>
<tr><td>통계분석 방법</td><td>결과분석의 일반적 원칙
유효성 평가에 대한 자료는 FAS 를 주 분석으로 한다. 안전성 평가에 대한 자료는 safety set 에서 평가한다. 별도 정의하지 않은 모든 통계 검정은 양측 검정, 5% 유의수준에서 검정한다.
유효성 평가 시 FAS 에 대해 결측치가 발생한 경우, Last Observation Carried Forward(이하, 'LOCF') 방법을 적용하여 통계분석을 실시하고, 그 외에는 원래 자료대로 통계분석을 실시한다.

<u>Full Analysis Set(FAS)</u>: ITT 원칙에 따르며, 한약제제 투여 후 주요 유효성 평가변수에 대한 측정이 1 회 이상 이루어진 대상자 집단
<u>Safety Set(SS)</u>: 중재를 1 회 이상 투여한 대상자 중, 안전성 관련 추적 관찰이 한번이라도 시행된 대상자 집단

일차 평가변수에 대한 분석
1) 12 주 중재의 복용 후 FSS 점수
: FSS 점수를 12주 중재의 복용 후 군간 비교한다.

이차 평가변수에 대한 분석
1) 최종 복약 순응도
: 최초 임상시험약의 투여 후 12 주째(방문 4 또는 종료시점) 전체 연구 대상자의 최종 복약 순응도(%)의 평균, 표준편차, 중앙값, 최소값 및 최대값을 구하여 제시한다.

2) 최종 복약 순응도의 군별 차이
: 최초 임상시험약의 투여 후 12 주째 (방문 4 또는 종료시점) 두 군의 최종 복약 순응도(%)의 평균, 표준편차, 중앙값, 최소값 및 최대값을 구하여 제시하고, 비교한다.</td></tr>
</table>

	3) 방문 2, 3 시점에서의 FSS 점수<br>: FSS 점수를 각 시점에서 군간 비교한다.<br><br>4) 방문 2, 3, 4 시점에서의 ChFS 점수 및 subscale 점수<br>: 총 점수 및 physical(1-7 번)/mental health(8-11 번) sub-scale 로 나누어 기록하고, 각 시점별 총 점수과 각 sub-scale 점수를 구하여 군간 비교한다.<br><br>5) 방문 4 시점에서의 EQ-5D-5L 점수<br>: EQ-5D-5L 의 점수를 산출하고, 각 시점 별 점수를 군간 비교한다.<br><br>6) 방문 4 시점에서의 PSQI-K 의 Global score 점수<br>: 대상자 설문지 중 7 종류의 component 점수를 구하고 이를 총 합산하여 구한 Global score 의 총점을 산출하고, 각 시점 별 점수를 군간 비교한다<br><br>7) 방문 4 시점에서의 K-MOCA 의 총점수와 영역별 점수<br>: 7 개 영역의 점수를 구하고, 이를 총 합산하여 총점수를 산출하여, 군간 비교한다.<br><br>8) 방문 4 시점에서의 BDI 점수<br>: 4 개 영역의 점수를 총 합산하여 총점수를 산출하여, 군간 비교한다.<br><br>9) 방문 4 시점에서의 DF 점수, DB 점수, DF-DB 점수<br>: 각 점수를 군간 비교한다.<br><br>10) 방문 4 시점에서의 전산화 신경인지기능 검사 점수<br>: 5 종 검사의 점수를 각각 군간 비교한다.<br><br>11) 방문 4 시점에서의 SPPB 검사의 보행속도 검사, 일어서기 검사, 정적균형 검사, 종합점수<br>: 3 종 검사의 점수와 종합점수를 군간 비교한다.<br><br>12) Feasibility 평가를 위한 대상자 모집율, 등록율, 중도탈락율, 중도탈락 이유 등에 대한 분석<br>: 대상자 모집율은 계획된 연구대상자 중 연구기간동안 모집한 대상자의 수로 계산한다. 대상자 등록율은 총 연구대상자를 총 스크리닝 대상자

	수로 나누어 계산한다. 중도탈락율 및 중도탈락의 이유를 전체 연구대상자 및 각 처방군별로 계산하여 비교한다.<br><br>13) 피로 증상에 대한 12 주 중재의 복용 후 치료군과 대조군 간의 치료성공률 : 최초 한약제제 투여 후 12 주에 피로증상에 대한 0-100 VAS 의 차이가 15 점이상인 경우 치료성공으로 정의<br><br>**안전성 변수에 대한 분석**<br><br>**이상반응**<br>이상반응은 임상시험용의약품을 투여한 시험대상자에게 발생한 모든 유해하지 않은 증후, 증상 또는 질병으로 정의하며 각 중재군 별로 모든 이상반응과 약물이상반응(ADR), 중대한 이상반응(SAE), 중대한 약물이상반응(SADR)에 대한 발생환자 수, 발생률, 발현건수 및 발생률에 대한 95% 신뢰구간을 제시하고 투여군 간 차이는 Chi-square test 또는 Fisher's exact test 로 검정한다. 모든 이상반응은 MedDRA(Medical Dictionary for Regulatory Activities)의 신체기관(SOC) 및 선호용어(PT)로 코드화하여 군 별로 빈도, 비율 및 건수를 제시한다.<br><br>**실험실 검사, 활력징후**<br>활력징후 및 정량적인 실험실적 검사치는 군별로 투여 전/후 그리고 변화량에 대해 기술통계량을 제시하고 변화량에 대한 군간 차이는 independent two sample t-test 또는 Wilcoxon rank sum test, 각 군내 변화의 차이는 paired t-test 또는 Wilcoxon signed rank test 로 검정한다. 추가적으로, 이분형 변수의 실험실적 검사 결과와 심전도 검사는 중재 투여 전/후 정상(임상적으로 의미 없는 비정상 포함)/임상적으로 의미 있는 비정상 변화에 대한 분할표를 제시하고 각 군 내 변화는 McNemar's test(또는 McNemar's Exact test)로 검정한다.

List of Abbreviations

**ADR**	**Adverse Drug Reaction**
**AE**	**Adverse Event**
**ALT**	**Alanine Transaminase**
**AST**	**Aspartate aminoTransferase**
**BDI**	**Becks' depression inventory**
**BMI**	**Body Mass Index**
**BUN**	**Blood Urea Nitrogen**
**CK**	**Creatine Kinase**
**CFS**	**Chronic Fatigue Syndrome**
**ChFS**	**Chalder Fatigue Scale**
**COVID-19**	**Coronavirus disease 2019**
**CRP**	**C-Reactive Protein**
**DB**	**Digit span backward**
**DF**	**Digit span forward**
**EPO**	**Erythropoietin**
**EQ-5D**	**EuroQol five dimensions questionnaire**
**FAS**	**Full Analysis Set**
**FSS**	**Fatigue severity scale**
**F/U**	**Follow-up**
**GCP**	**Good Clinical Practice**
**Hb**	**Hemoglobin**
**HBV**	**Hepatitis B Virus**
**Hct**	**Hematocrit**
**HCV**	**Hepatitis C Virus**
**HIV**	**Human Immunodeficiency Virus**
**ICH**	**International Conference on Harmonisation**
**IRB**	**Institutional Review Board**
**ITT**	**Intent To Treat**
**K-MOCA**	**Korean-Montreal cognitive assessment**
**K-WAIS-IV**	**Korean Wechsler Adult Intelligence Scale-IV**
**LDH**	**Lactate DeHydrogenase**
**LOCF**	**Last Observation Carried Forward**
**MedDRA**	**Medical Dictionary for Regulatory Activities**
**PSQI-K**	**Korean version of Pittsburgh Sleep Quality Index**
**RBC**	**Red Blood Cell**
**SADR**	**Serious Adverse Drug Reaction**
**SAE**	**Serious adverse event**
**SPPB**	**Short physical performance battery**
**TSH**	**Thyroid Stimulating Hormone**
**UNL**	**Upper Normal Limit**
**WBC**	**White Blood Cell**

Schedule summary

Visit	0	1	2	3	4	UV*
**Week (day)**	**-1 (-7)**	**1(0)**	**4 (28)**	**8 (56)**	**12 (84)**	**-**
**Visit window (day)**	**-**	**+2**	**±2**	**±2**	**±2**	**-**
서면동의	●					
인구학적 조사	●					
타 임상시험 참여 여부 조사	●					
신체 검사(Physical Exam)	●					
과거력 및 현병력 조사	●	●				
COVID-19 확진에 관한 조사	●					
병용약물/치료 조사	●					
Vital sign 측정	●	●	●	●	●	●
심전도 검사	●				●	●
실험실적 검사	●				●	●
면역, 대사체검사(혈액채혈)	●				●	●
임신여부 평가**	●					
피로 증상 평가***	●					
FSS(Fatigue Severity Scale)	●		●	●	●	●
선정, 제외기준 평가	●					
무작위배정		●				
배정된 중재(경옥고 또는 위약)의 처방 및 교부		●	●	●		
약물복용량의 측정			●	●	●	●
병용약물조사			●	●	●	●
피로의 0-100 VAS(Visual analogue scale)		●	●	●	●	●
ChFS(Chalder Fatigue Scale)		●	●	●	●	●
EQ-5D-5L		●			●	●
PSQI-K(Korean version of Pittsburgh Sleep Quality Index)		●			●	●
K-MOCA(Korean-Montreal cognitive assessment)		●			●	●
BDI(Becks' depression inventory)		●			●	●
숫자 바로/거꾸로 외우기 검사		●			●	●
전산화 신경인지기능 검사 점수		●			●	●
SPPB(Short Physical Performance Battery)		●			●	●
이상반응 조사			●	●	●	●

●해당 방문에 시행; *UV: Unscheduled visit (이상반응 등의 이유로 원래 방문일정이 아닌 날 방문하는 경우); **가임기 여성만 해당; ***선정기준 평가를 위하여 COVID-19 에 이환된 이후 피로가 발생하였는지 여부 조사

1. 임상시험 명칭과 단계

만성 코로나19증후군(Long COVID) 환자의 피로에 대한 경옥고의 효과 평가 임상시험: 이중맹검, 무작위배정, 다기관, 예비, 연구자임상시험

단계: 해당사항 없음

2. 임상시험 실시기관명과 주소

경희대학교한방병원(서울특별시 동대문구 경희대로 23)

동의대학교부속한방병원(부산광역시 부산진구 양정로 62)

3. 임상시험의 책임자

실시기관		성명	직위
경희대학교한방병원	한의약임상시험센터	김태훈	교수
동의대학교부속한방병원	한방신경정신과	권찬영	교수

4. 임상시험 지원기관

한국한의학연구원 (대전광역시 유성구 유성대로 1672, 042-861-1994)

5. 임상시험의 배경과 목적

1) 연구 배경

코로나바이러스감염증-19 완차자의 후유증 현황

코로나바이러스감염증-19(이하 COVID-19)의 전세계적인 대유행으로 인해 감염증의 예방을 위한 백신의 개발과 감염 전파의 차단, 급성감염증의 치료에 의료 및 연구역량이 총 결집되고 있는 것이 현실이다. 그런데 생명을 위협하는 급성감염증 뿐만 아니라, COVID-19의 완치 이후 후유증 또한 광범위하게 보고되고 있다. 미국의 미시간대학 연구팀은 2020년도 봄부터 여름까지 미국 미시간주의 38개 병원에 입원했던 중증 COVID-19 환자 1,250 명의 퇴원 후 후유증 유무에 대하여 전화 인터뷰를 통하여 조사한 결과 인터뷰에 응한 488명의 중증 퇴원 환자 중 약 39%가 두 달이 지나도록 정상적인 활동을 못 하고 있다고 응답하였으며, 특히 응답자의 12%는 COVID-19에 걸리기 전처럼 일상적인 기본 관리를 혼자서 해내기 어렵다고 보고하였다. 또한 23%는 계단을 오를 때 호흡이 가쁘다고 했고, 3분의 1은 미각이나 후각 이상 같은 COVID-19 급성 증상이 사라지지 않았다고 호소했다. 게다가, 건강 문제로 이전의 직장에 돌아가지 못한 환자가 40%에 달했고, 일부는 실직하였으며, 직장에 복귀한 사람 중에서도 26%는 건강 문제로 근무 시간을 단축하였다고 응답하였다 [1]. 따라서, COVID-19의 급성기 증상에 못지않게, 완치 후 후유증에 의한 영향도 상당히 심각함을 알 수 있다.

최근에 발표된 코로나의 후유증 유병률에 관한 연구에 의하면, 피로는 735,006명의 COVID-19 환자 중 입원 환자의 28.4%, 외래 환자의 34.5% 정도가 호소하는 가장 흔한 증상이다 [2]. 아일랜드 더블린 트리니티대 연구진은 더블린에 위치한 세인트 제임스병원에 입원했거나 외래 진료를 받은 COVID-19 환자 128명을 대상으로 추적 조사를 진행한 결과 이들 중 절반 이상(52.3%)이 COVID-19 확진 판정 이후 최소 6주간 피로감을 호소하였다고 보고하였다. 이들 중 심한 경우 최장 10주까지 피로감을 가지고 있었다. 이에 반면 COVID-19 후유증이 없다고 답한 사람은 54명(42.2%)이었다. 이 연구에서 흥미 있는 결과는 조사 대상의 COVID-19 중증도와

피로감 사이의 관계를 조사했지만, 상관관계를 찾을 수 없었다고 밝혔다. 곧 COVID-19의 절반 이상의 완치자에서 증상의 경중에 상관없이 피로감을 호소하고 있음을 알 수 있다 [3]. COVID-19 완치 후 경험하는 증상은 매우 다양하며, 명확한 기전이 아직 밝혀지지 않았다. 다만, 다양한 종류의 바이러스 감염질환에 이환되었던 환자에게서 만성피로증후군(Chronic fatigue syndrome, CFS)이 관찰된다는 보고가 있고, 2003년도에 유행했던 SARS 확진 후 생존자의 약 27%가 확진 후 수년이 지나도 CFS 범주에 해당하는 증상을 보이는 것으로 알려졌다[4]. 따라서, 이들 증상은 광범위한 의미에서 바이러스후피로증후군(post-viral fatigue syndrome)의 범주에 해당한다고 보는 것이 타당할 것이다.

이러한 상황은 국내에도 동일하게 적용된다. 국내 중앙방역대책본부는 9월 29일 정례브리핑에서 "COVID-19에 걸렸다가 완치된 뒤 겪는 후유증을 조사한 결과, 완치자 중 90% 이상이 피로감과 집중력 저하 등의 후유증을 겪는 것으로 나타났다"고 밝혔다. 권준욱 중앙방역대책본부 부본부장은 "경북대병원의 경우 전체 5,762명의 대상자에 대해 COVID-19 완치 후 후유증에 대한 답변을 구해, 그중 참여자 965명이 응답했다"며, "그중 91.1%에 해당하는 879명의 완치자가 최소 1개 이상의 후유증이 있다고 답변했다"고 보고하였다. 국내의 대표적인 완치 후 후유증으로는 피로감(26.2%), 집중력 저하(24.6%)을 비롯해 심리적·정신적 후유증, 후각·미각 손실 등을 보고하였다[5]. COVID-19의 관리방안 중 후유증기 치료에 대한 필요성이 요구되는 상황이라는 것을 알 수 있다.

만성 코로나19증후군(Long COVID)의 가설적 기전 및 통상치료

흔히 코로나19 후유증으로 불리는 만성 코로나19증후군(long COVID)에 대한 정의는 명확히 정립되지 않았으며, 여러 나라와 기관마다 다른 용어나 정의를 사용하고 있다. WHO는 보통 코로나19 증상발현 이후 3개월 이내 발생하여 최소 2개월 동안 지속되는 다른 대체 진단으로 설명될 수 없는 증상을 Post-COVID Conditions로 정의하였다[6]. 미국 CDC는 코로나19 감염 4주

후에도 증상이 지속되는 경우 Post-COVID Conditions(Long COVID)로 정의하였으며, 영국 NICE는 코로나19 증상이 4-12주 지속되는 경우 Ongoing symptomatic COVID-19, 코로나19 진단 12주 이후에도 증상이 지속되는, 다른 진단으로 설명되지 않는 경우 Post-COVID syndrome으로 정의하였다[7].

국내의 경우 코로나19 이후 후유증에 대한 연구 및 사례정의 등을 위해 용어 통일이 필요하며, 이를 위해 질병관리청과 대한감염학회의 논의를 통해 용어 및 정의가 확립되었으며, 이는 코로나 진단 12주 이후에 다른 질환으로 설명되지 않는 하나 이상의 증상/징후가 지속되는 증상을 만성 코로나19증후군(long COVID)으로 정의하고 있다. 코로나19 급성기 또는 아급성기 합병증으로 정맥혈전색전증, 심근염, 심낭염, 뇌염 및 갑상선염 등이 발생할 수 있으며, 이를 만성 코로나19증후군으로 분류하지는 않으며, 일반적으로 피로감, 호흡곤란, 우울・불안, 인지저하 등 증상을 이 범주에서 이해하는 경향이다[8].

피로감

피로는 코로나19 환자들의 흔한 호흡기 외 증상 중 하나로, 보통 41% 정도에서 호소하며, 감염 후 4주 째에 35-45%, 8주째에 30-77%, 12주 째에 16-55%에서 나타난다고 보고되고 있다. 코로나19 환자들의 피로는 중동호흡기증후군(Middle East respiratory syndrome, MERS)이나 중증급성호흡증후군(severe acuter respiratory syndrome, SARS), 지역사회획득폐렴 이후에 설명되는 만성피로증후군(chronic fatigue syndrome)과 유사한 특징을 가지는데, 아직까지 코로나19와 연관된 장기간의 피로 증상과 코로나19의 중증도, 혹은 염증과 관련된 실험실적 검사수치들 사이의 연관성은 보고되지 않았고, 다만 이전에 우울이나 불안을 진단받은 여성 환자들에게서 과도하게 피로 증상이 발생되는 것이 보고되었다. 만성 코로나19증후군에서 나타나는 피로 증상은 단순한 피로보다 더 광범위하고 심각할 수 있으며 여기에는 인후통, 몸 전체의 통증 및 통증, 혈압 변화, 과민성 대장 증후군과 같은 위장 장애, 두통, 수면 장애, 우울증

및 현기증이 포함될 수 있다. 또한 새로운 민감성 또는 알레르기 반응, 사지의 작열감 또는 따끔거림을 포함하여 더 심각한 신경 학적 증상이 발생할 수도 있다. 또한 이 질환의 주요 특징은 최소한의 신체적 또는 정신적 활동만으로 증상이 갑자기 악화될 수 있다는 것이다. 증상은 본질적으로 만성 피로 증후군(근육성 뇌척수염 또는 ME라고도 함)의 증상과 동일하므로 WHO는 이러한 증상을 동일한 범주의 신경계 장애로 분류하며, 바이러스 후 피로 증후군에 대한 임상 평가는 만성 피로 증후군에 대한 임상 평가와 동일하게 진행하고 있는 실정이다. 이러한 이유는 바이러스 감염증 후 증상이 발생하는 기전에 대해서는 아직까지 명확하게 설명할 수 없다는데 기인한다. 체내에 잠복할 수 있는 바이러스의 비정상적인 반응이나, 염증을 촉진하는 전염증성 사이토카인 수치 증가, 신경조직 염증 등이 이유가 거론되고 있으며, 특히 이중에서 ‘사이토카인 폭풍(Cytokine storm)’이라고 불리우는 현상 곧 IL-2, IL-7, 과립구, 인터페론 감마, monocyte, TNF-alpha 등의 사이토카인이 바이러스등에 의해 급증하고, 이로 인한 다발성 장기의 손상 등을 유발하게 되면, 감염증이 회복된 이후에도 정상기능으로 회복하지 못하게 된다고 하고, post-encephalitis syndrome과 같은 장애를 초래하며, 염증반응경로 상 관련 사이토카인들의 신호전달체계에 문제가 발생하게 되어 피로 등의 증상이 유발된다고 추정하고 있다. 결국 만성 코로나19증후군에서 나타나는 다양한 후유증들은 개인의 면역력을 회복하고, 장기의 기능을 개선시키는 등 전신적인 접근이 필요할 것으로 예측된다[9]. 하지만 만성 코로나19증후군 환자의 피로 관리를 위한 치료 및 중재의 효과를 뒷받침하는 높은 근거 수준의 연구는 현재 부족한 상황이며[8], 대증요법 및 생활관리 위주의 치료가 제공되고 있어서, 이 분야에 대한 연구가 시급한 상황이다.

COVID-19의 후유증에 대한 경옥고의 근거

만성피로증후군에 대한 문헌 연구에서 한의학에서는 피로증후군에 대한 문헌 연구를 통해, 비기휴허(脾氣虧虛), 간비불화(肝脾不和), 기허혈어(氣虛血瘀), 간신음허(肝腎陰虛), 비신양허(脾腎陽虛)가 만성피로증후군의 주된 변증분형이며, 이 중 비기허가 가장 주된

변증분형이 된다고 주장하였다[10]. 최근 우리나라 한의사를 대상으로 이루어진 조사연구에서, 한약은 만성피로증후군과 특발성만성피로환자의 치료 또는 증상 관리를 위해 가장 효과적이고 널리 사용되는 치료 수단이며, 한약 처방 중에서 보중익기탕과 쌍화탕, 십전대보탕, 귀비탕, 팔물탕, 공진단, 경옥고 등의 보제가 다빈도로 선택되는 기본방인 것으로 조사되었다[11]. 이 중 경옥고는 병후 발생하는 기음양허형 피로와 제반증상의 개선을 목적으로 임상에서 활용되고 있다. 경옥고(600mg/kg)를 실험동물 ICR 마우스에 4주간 처리한 결과 유의성 있는 혈중 젖산 감소와 혈중 글루코스 증가 및 골격근 내 글리코겐 함량 증가가 관찰되었으며, 악력 증진, 탈진 트레드밀 운동기능 향상, 강제 수영운동 지속 시간 증가 등 항피로 효능 및 운동수행능력 개선 활성이 관찰되었다. 이는 경옥고가 피로 개선에 효과를 보일 수 있는 전임상 근거로 판단해 볼 수 있다[12]. 임상근거로서 경옥고가 점진적 운동부하 후 젖산 및 암모니아의 감소에도 효과가 있는 것으로 보고되었으며[13], 축구선수를 대상으로 한 연구에서 최대산소섭취량의 증가와 운동 후 심박수 회복률을 증가시킴으로써, 유산소성 운동능력을 증가시키고 운동 후 피로회복에 도움을 줄 수 있다는 연구결과 또한 보고되었다[14]. 해당 연구에서는 4주간 경옥고의 복용과 운동으로 인한 간기능 손상 여부를 관찰하기 위하여 살펴본 SGOT, SGPT는 집단 간에는 유의한 차이는 없었다. 또한 섭취 후나 운동 후 모두 정상범위(40 U/L 이하)내 수치였으므로 간 기능손상 등의 우려는 없는 것으로 파악된다[14].

더욱이 한국에서 2020년 전반기 COVID-19에 대한 한의 치료를 받은 환자군이 가장 많이 처방받은 약은 경옥고였고, 한약 치료를 받고 피로를 포함한 COVID-19 관련 증상 전반이 개선되었다고 보고되었다[15]. 피로 외에도 경옥고를 신경 염증이나 신경 퇴행성 질환에 활용할 수 있는 가능성을 확인한 연구도 있다. 해당 연구에서는 Mouse의 미세아교세포주인 BV2 cell에서 염증시 발생하는 NO, iNOS, COX-2, 및 각종 cytokine을 억제하는 효과가 경옥고에 있다고 보고하였다[16]. 또한 경옥고에 대한 문헌 연구에 따르면 경옥고의 항산화, 항암, 항염증, 면역 및 성장 증진 등의 효과를 통해 중추신경계, 심혈관계, 소화기계, 호흡기계 등의 다양한 질환에

독성이나 부작용 없이 적용할 수 있다고 하였다[17]. 식품의약품안전처에 등재된 한약제제 중 경옥고는 병중병후, 허약체질, 육체피로 등의 적응증이 표기되어 있어, 만성 코로나19증후군에 수반되는 피로 증상을 완화시키는데 활용 가능할 것으로 판단되며, 이에 대한 임상적 근거를 마련하는 것이, 만성 코로나 19 증후군대한 한의학적인 관리방안을 마련하기 위한 기초 자료로 활용될 수 있으므로 중요하다.

2) 연구 목적

본 연구는 COVID-19에 확진되고 완치된 이후 만성 코로나19증후군(Long COVID)의 대표 증상 중 피로를 호소하는 사람들을 대상으로 시판중인 한약제제를 투여한 후 안전성 관련 지표와 유효성 관련 지표를 평가하는 전향적 예비임상시험으로, 해당 중재와 연구설계의 적용가능성(feasibility)를 평가하고, 향후 대규모 연구를 위한 근거를 마련하는 것을 연구목적으로 한다.

본 연구의 1차 목적은 만성 코로나19증후군 환자의 피로에 대한12주 한약제제(경옥고) 복용 후 FSS(**Fatigue severity scale**)의 변화를 탐색해 보는 것이다. 2차 목적은 연구기간동안 대상자의 모집율과 중도탈락율 등 연구설계의 적용가능성(feasibility)을 평가하는 것이다. 더불어 COVID-19완치 후 한약제제 복용이 COVID-19 면역반응에 미치는 영향 및 피로와 유관한 대사체(metabolites)에 미치는 영향을 평가하는 것을 부수적으로 탐색하는 것을 목적으로 한다.

6. 대상질환

COVID-19에 감염된 이후에 발생한 지속되는 피로

7. 대상자 선정

1) 선정 기준

- 만19세 이상 성인으로 COVID-19에 확진 된 후 최소 12주를 경과한 자
- COVID-19에 확진 되기 이전에 경험하지 않았던 피로를 지난 4주이상 지속적으로 경험하는 자
- FSS 점수가 4점 이상인 자
- 전반적 인지기능에 문제가 없고, 자발적으로 서면동의를 통해 연구 참여에 동의한 자

2) 제외 기준

- 피로를 유발할 수 있는 기질적인 질환(암, 수면장애, 만성 간염, 간경화, 만성 신부전, 결핵, 천식, 다발성경화증)의 과거력 혹은 현병력이 있는 자
- 약물의 복용이나 흡수에 영향을 줄 수 있는 질환(연하장애, 임상적으로 심각한 소화장애, 갈락토오스 불내성(galactose intolerance), Lapp 유당분해효소 결핍증(Lapp lactase deficiency), 포도당-갈락토오스 흡수장애(glucose-galactose malabsorption) 등의 유전적인 문제 등)이 있는 경우
- 조절되지 않는 당뇨환자
- 임상시험용 의약품(한약제제)에 대한 알러지 과거력이 있는 경우
- 간질환이나 신장질환의 과거력이 있거나 혈액검사상 AST, ALT, BUN, Creatinine이 정상상한치의 3배를 초과하는 경우
- 임신 중이거나, 임신가능성 있거나, 수유 중인 여성
- 임상시험 참여 전 30일 이내에 다른 연구에 참여한 경우
- 2주 이내에 한약이나 생약 제제(임상시험용의약품의 구성 약재와 동일한 한약(생약)이 포함된 경우)를 복용한 경우
- 임상적으로 유의한 정신과적인 증상이나 의학적인 질환, 검사실 소견 등에 의해 연구 참여가 어렵다고 연구자가 판단한 경우

3) 목표한 대상자 수와 산출 근거

본 연구의 경우 전향적 예비 연구자주도 임상시험로, 본 연구를 통해 얻은 결과를 이용하여 향후 확증적 임상시험을 위한 연구대상자 수를 계산할 계획이다. 연구대상자는 치료군(경옥고투여군) 50명, 대조군(위약투여군) 50명으로 총 100명을 모집한다.

실시기관	치료군	대조군	합계
경희대학교한방병원	30	30	60
동의대학교부속한방병원	20	20	40
합계	50	50	100

8. 대상질환의 표준 치료 방법

COVID-19에 이환된 이후에 발생한 피로 증상의 해결을 위해 다양한 약물, 대체의학, 인지 행동 치료, 운동 등 여러 방법들이 현재 시도 되고 있다. 일부 연구에서 만성피로증후군의 치료에 활용되는 rintatolimod라는 약물과 상담요법 및 단계별 운동요법이 이점이 있음을 시사했지만, 이들 중재의 근거가 아직까지는 제한적이다. 미국 질병관리본부(CDC)에서도 COVID-19이후 발생한 피로를 만성피로증후군(Chronic Fatigue Syndrome/Myalgic Enchephalomyelitis, CFS/ME)의 범주하에서 설명하고 있으며, 명상, 요가와 테라피와 같은 부드러운 운동, 상담, 식이보조제의 투여, 휴식 등 증상 관리에 초점을 두고 있다[18]. 영국의 NHS에서도 휴식, 활동수준을 낮추는 것, 점진적인 휴식과 운동, 수면의 조절, 균형잡힌 식생활, 집중력 훈련 등을 증상 관리의 측면에서 관리방안을 권고하고 있다[19].

9. 임상시험에 사용되는 의약품

9.1임상시험용의약품의 코드명, 원료 약품의 분량, 제형(성상) 등등

시험약

코드명(제품명)	CV1(진경옥고)			
제조회사	경진제약㈜			
투여경로	경구			
성상 및 제형	알루미늄 스틱포에 충전된 흑갈색의 점도가 있는 연조엑스제			
성분 및 함량 (주성분)	(이 약 112.5 중)			
	원료명	규격	분량	단위
	생지황즙	별첨규격	39.9	g
	복령	KP	12.4	g
	인삼	KP	6.2	g
	꿀	JP	41.5	g
사용기한	제조일로부터 36 개월			
저장방법	기밀용기, 실온보관(1~30°C)			

위약

코드명(제품명)	P(위약)			
제조회사	경진제약㈜			
투여경로	경구			
성상 및 제형	알루미늄 스틱포에 충전된 흑갈색의 점도가 있는 연조엑스제			
성분 및 함량 (위약)	이 약 112.5 g 중			
	원료명	규격	분량	단위
	정제수	KP	65.0	g
	벤조산나트륨	KP	0.06	g
	시트르산수화물	KP	0.09	g
	시트르산나트륨수화물	KP	0.04	g
	고과당55	별규	21.0	g

	백당	KP	14.0	g
	카라멜	NF	5.0	g
	잔탄검	EP	3.8	g
	농글리세린	KP	3.5	g
	진생후레바에이-980306	별규	0.01	g
사용기한	제조일로부터 36개월			
저장방법	기밀용기,실온보관(1~30°C)			

9.2 용량 선정의 근거

본 임상시험에서 제공되는 한약제제는 식품의약품안전처에서 품목허가된 의약품을 투여하며, 용량 및 용법도 모두 허가사항을 준수하여 사용한다. 품목허가서에 기재된 대로 CV1(진경옥고)는 성인 1회 22.5g(1포) 투여하며, 1일 2회 아침, 저녁 식전 또는 식간에(식사 때와 식사 때 사이에) 복용한다. P(위약)의 경우도 시험약과 동일하게 성인 1회 22.5g(1포) 투여하며, 1일 2회 아침, 저녁 식전 또는 식간에(식사 때와 식사 때 사이에) 복용한다.

9.3 포장과 라벨

임상시험용 의약품은 임상시험 의뢰자가 제조 후, 포장 및 라벨링을 수행하여 임상시험 기관의 관리약사에게 공급한다. 시험약과 대조약의 생산과 포장은 모두 GMP 설비에서 GMP 규정에 따라 수행한다. 임상시험용 의약품 라벨의 기재는 의약품 제조 및 품질관리에 관한 규정(2017.01.01 제정) 별표 11에 따라, 이 약의 포장에는 아래의 내용이 포함된 라벨을 제작한다. 단, 임상시험용 의약품 1차 포장에는 (*) 항목만을 표기하고, 2차 포장에는 전체 항목을 모두 표기한다. 대상자 배정 번호의 경우 1차포장에서는 표시하지 않고, 해당 내용을 서류화 하여 보관 한다.

▌대상자 배정 번호
▌참조 코드*
▌임상시험용의약품 명칭*

▌유효기한 또는 재검사일자
▌배치번호 또는 코드번호*
▌보관조건
▌복용(섭취)방법
▌**임상시험계획 승인을 받은 자의 명칭과 주소 및 전화번호***
▌어린이의 손이 닿지 않는 곳에 보관한다.
▌**임상시험 외의 목적으로 사용할 수 없음(일차포장은 "임상시험용")***

9.4 임상시험용 의약품의 관리

임상시험용 의약품의 인수, 보관, 조제 및 반납에 대한 책임은 해당 실시기관의 연구책임자와 관리약사에게 있다. 임상시험용 의약품은 실시기관의 관리약사(또는 연구책임자)에게 전달되고, 관리약사(또는 연구책임자)는 공급받은 모든 임상시험용 의약품의 수령사실 및 수량을 서면으로 확인하고 서명해야 하며, 적절히 관리해야 한다. 임상시험용 의약품은 시건 장치가 되어 있으며, 접근이 제한된 보관장소에 보관되어야 하며, 임상시험용 의약품이 임상시험계획서에 의하여 연구대상자에게 투여되도록 하고, 각 연구대상자에게 지급된 임상시험용 의약품의 수량 및 관리에 대한 기록을 관리한다. 모니터는 사용된 임상시험용 의약품의 수불을 확인하기 위하여 연구자나 관리약사에 의해 보관된 재고량을 정기적으로 확인해야 한다. 관리약사(또는 연구책임자)는 연구대상자에게 지급된 임상시험용 의약품의 사용 후 빈포장용지 및 잔량을 반납받아야 하고, 연구대상자로부터 반납받는 임상시험용 의약품과 미사용 임상시험용 의약품, 사용/미사용 포장용지와 약품을 연구책임자에게 반납한다.

9.5 병행치료

허용되는 병용투여 약물 및 치료

피로에 대한 약물치료(한약, 건기식 등) 및 시험약의 구성 약재가 포함된 한약의 복용을 금한다. 또한 인지행동치료나 상담 등 의학적인 치료에 참여하는 것을 제한한다. 다만 일반적인 건강을 증진시키기위한 운동 등은 허용한다.

10. 연구기간

IRB승인 후 3년

11. 연구방법

11.1 연구설계

환자는 본 임상시험에 대한 충분한 설명을 듣고 동의서에 서명한 다음 연구대상자로서 적합한지 스크리닝 과정을 거친다. 연구대상자로 적합한 환자는 무작위배정을 통해 치료군(CV1, 진경옥고 투여군)과 대조군(P, 위약군)으로 배정한 후 12주간 해당 중재를 투여 받게 된다. 각 대상자는 중재 투여기간동안 한달간격으로 방문하며, 12주 중재 시행 종료 후 증상평가 및 혈액 등의 검체 채취를 위한 방문을 시행하고 연구를 종료한다. 연구대상자의 모집을 위해 원내외 광고를 통하여 대상자들을 모집할 계획이다.

11.2 한약제제의 투여량 및 투여 방법

연구기간 중 한약제제의 용법·용량은 다음에 따른다.

[용법·용량]

코드명(한약제명)	용법	1 회투여단위(용량)
CV1(진경옥고)	●/○/●	1포(22.5g)
P(위약)	●/○/●	1포(22.5g)

●복용, ○복용하지 않음

[한약제제의 투여방법]

정해진 용법에 따라 1일 2회 식전 혹은 식간에 복용한다.

11.3 눈가림 등

연구자는 방문 0(Visit0)에 각 연구대상자에게 고유한 스크리닝번호(Screening number)를 배정한다. 해당 번호는 경희대학교한방병원은 CVS1-01, 동의대학교한방병원은 CVS2-01로 시작하며, 배정된 일련 번호 중 사용 가능한 최소 번호로 구성된다. 한번 배정된 대상자 번호는 재사용하지 않는다. 스크리닝을 통해 연구대상자로 선정이 되면 대상자배정번호(Random number)를 부여받게 되며, 번호는 경희대학교한방병원은 CVR1-01, 동의대학교한방병원은 CVR2-01로 시작하고, 배정된 일련 번호 중 사용 가능한 최소 번호로 구성된다. 이유를 불문하고 방문 1(V1)에서 대상자의 치료가 시행되지 않은 경우, 해당 대상자 번호의 부여와 치료가 시행되지 않은 사유를 선별검사 기록(Screening Log) 및 증례기록서(case report form [CRF])에 기재한다.

블록 무작위 배정을 시행할 계획이며, 맹검의 유지를 위해 블록의 크기는 공개하지 않는다. STATA Version 4.2 (StataCorp LLC., Texas) 통계프로그램을 이용하여 독립적인 통계학자가 무작위번호를 생성하고, 번호를 시험약의 라벨링하는 곳으로 전송하여 무작위 배정 순서에 따라 시험약과 대조약을 포장하고 대상자 배정 번호(Random number)를 라벨에 기입한다. 이를 통해 연구자와 연구대상자 모두 눈가림될 수 있다. 치료군과 대조군의 배정확률은 1:1이다.

배정은폐(allocation concealment)를 위하여 독립적인 통계학자가 생성한 무작위번호에 따라 포장된 임상시험약을 임상시험 실시기관에 전달하면, 임상시험에 대해 충분한 설명을 듣고 선정기준을 충족하고, 제외기준에 해당하지 않으며, 자발적으로 서면 동의를 한 대상자에 대해 각 기관의 시험자는 대상자가 연구에 참여한 순서대로 사용 가능한 최소 번호부터 대상자배정번호를 부여하고, 이 번호를 증례기록서에 기재한 후, 시험약을 교부한다.

11.4 관찰 및 검사항목

11.4.1 임상시험 진행 일정

11.4.1.1 방문 0(스크리닝 방문)

이 임상시험에 참가하도록 지원한 대상자는 연구에 대한 설명을 듣고 다음에 해당하는 검사를 받는다. 스크리닝 검사는 1회에 한하여 스크리닝 기간 내에 재검사를 실시할 수 있으며, 대상자 번호 부여 전에 완료한다. 재검사가 실시된 경우 대상자의 참여 적합성 여부는 재검사 결과를 기준으로 하며, 시험자는 재검 사유, 판단 근거, 최종 결과 등에 대해서 근거문서에 가능한 상세하게 기록한다.

① 임상시험 대상자를 참여시키기 전에 연구책임자 또는 담당자는 연구 과정을 설명하고, 대상자 동의서에 서면 동의를 받는다.
② 동의서를 작성한 순서대로 대상자 번호(Screening number) 대상자배정번호를 부여 받는다.
③ 타 임상시험 참여여부(30 일 이내), 인구학적 정보와 의학적 병력(당뇨 등), COVID-19 정보(발병일, 완치일, 백신 접종(날짜, 제조사, 횟수 부작용 등)), 피로와 관련된 과거 병력, 현재 피로 증상 유무를 조사/기재한다. 이때 당뇨치료를 받고 있더라도, 검사결과상 조절되면 연구에 참여할 수 있다(HbA1c 가 정상범위인 경우)
④ 신체검사(Physical Exam)는 일반상태, 영양상태, 피부/점막, 눈(시력손상 제외), 이비인후계,갑상선,폐,심장/순환계,복부,신장/비뇨기계,신경/정신계,척추/사지/종양, 말초순환, 림프계 에 대해서 조사 한다.
⑤ COVID-19 확진 여부에 대하여 가능한 경우 다음의 항목 조사를 통해 확진여부와 확진일 등을 조사/기재하고, 확진 당시의 증상에 대한 평가(조기경고점수(EWS))를 실시한다.
   - 질병관리청의 확진관련 안내 문자메시지
   - COVID-19 확진 및 치료 당시 의무기록, 진단서 등
   - 확진당시 PCR 검사 기록
   - 코로나19 확진 또는 확진 후 격리해제 등과 관련된 공공기관에서 발급된 증명서 등
⑥ 복용약물을 확인한다.
⑦ Vital sign 을 측정한다.
⑧ 심전도 검사를 실시한다.
⑨ 실험실적 검사 및 면역, 대사체검사를 위해 채혈한다(실험실적 검사의 경우 스크리닝 방문 기준으로 4 주(28 일) 이내 실시한 검사 결과 사용 가능).
   - 혈액화학: AST, ALT, γ-GTP, ALP, HbA1c(검사 이상시 당뇨로 판단), BUN, creatinine, total bilirubin, Glucose

- 면역검사: COVID-19 특이항체/중화항체 검사, 단일세포 전사체 분석 (scRNA seq.)을 이용한 면역관련유전자발현 비교분석, FACS 를 이용한 면역표현형 (immunophenotyping) 분석, 혈청내 사이토카인 수준 검사
- 대사체검사: 일차 및 이차 대사체 프로파일링

⑩ 가임 여성을 대상으로 임신반응 검사(Urine 또는 Serum β-hCG)를 실시한다(스크리닝 전 28 일 이내 검사가 있는 경우 사용 가능).

⑪ 대상자는 시험자의 지시에 따라 아래에 해당하는 평가를 실시한다.
- FSS 를 통한 피로도평가[20]
- 피로 증상이 4 주 이상 지속되었는지 여부 평가

⑫ 선정/제외기준을 확인한다.

11.4.1.2 방문 1(중재시작, 1 주)

스크리닝 방문일에 모든 검사 및 적합성 평가가 완료된 경우, 스크리닝 방문 당일에 방문 1 을 실시하여 대상자 번호를 부여할 수 있다. 스크리닝 방문과 대상자 번호 부여일이 동일한 경우, 중복되는 평가는 1 회만 실시한다.

① Vital sign 을 측정한다.
② 과거력 및 현병력 조사(방문 0 과 방문 1 사이의 변동사항)를 실시한다.
③ 대상자배정번호(Random number)를 부여한다.
④ 대상자배정번호에 해당하는 한약제제를 4 주분량 처방한다.
⑤ 대상자는 시험자의 지시에 따라 아래에 해당하는 평가를 실시한다.
- 0-100 VAS 를 통한 피로증상의 중증도 평가
- ChFS(Chalder Fatigue Scale)를 통한 피로도 평가
- EQ-5D-5L 을 통한 삶의 질 평가
- PSQI-K(Korean version of Pittsburgh Sleep Quality Index)를 통한 수면상태 평가
- K-MOCA(Korean-Montreal cognitive assessment)를 통한 인지기능 검사
- Becks' depression inventory (BDI)를 통한 우울증 평가
- 숫자 바로/거꾸로 외우기 검사
- 전산화 신경인지기능 검사
- 간단 신체 수행 검사(Short Physical Performance Battery)

11.4.1.3 방문 2, 3(중간 방문, 4 주(28 일) ±2 일, 8 주(56 일) ±2 일)

중간 방문에는 아래에 해당하는 검사를 실시한다.

① Vital sign 을 측정한다.

② 지난 방문 때 처방한 한약제제를 반납 받은 뒤 복용량을 확인한다.
③ 대상자는 시험자의 지시에 따라 아래에 해당하는 평가를 실시한다.
  - FSS 를 통한 피로도평가
  - ChFS(Chalder Fatigue Scale)를 통한 피로도 평가
  - 0-100 VAS 를 통한 피로증상의 중증도 평가
④ 대상자배정번호에 해당하는 임상시험약을 4 주분량 처방한다.
⑤ 병용약물조사를 실시한다.
⑥ 임상시험약 투여 후 발생한 이상반응에 대해 확인한다.

11.4.1.4 방문 4(투여 종료, 12 주(84 일) ±2 일)

방문 4 에서 임상시험약의 투여는 전부 종료되며, 아래에 해당하는 검사를 실시하고 임상시험이 종료된다.

① 지난 방문 때 처방한 한약제제를 반납 받은 뒤 복용량을 확인한다.
② Vital sign 을 측정한다.
③ 혈액 검사 및 심전도 검사를 실시한다.
  - 혈액화학: AST, ALT, BUN, creatinine
  - 심전도검사: EKG
④ 면역, 대사체 검사를 위한 채혈을 실시한다.
  - 면역검사: COVID-19 특이항체/중화항체 수준 검사, 단일세포 전사체 분석 (scRNA seq.)을 이용한 면역관련유전자발현 비교분석, FACS 를 이용한 면역표현형 (immunophenotyping) 분석, 혈청내 사이토카인 수준 검사

  - 대사체검사: 일차 및 이차 대사체 프로파일링
⑥ 대상자는 시험자의 지시에 따라 아래에 해당하는 평가를 실시한다.
  - FSS 를 통한 피로도평가
  - ChFS(Chalder Fatigue Scale)를 통한 피로도 평가
  - 0-100 VAS 를 통한 피로증상의 중증도 평가
  - EQ-5D-5L 을 통한 삶의 질 평가
  - PSQI-K(Korean version of Pittsburgh Sleep Quality Index)를 통한 수면상태 평가
  - K-MOCA(Korean-Montreal cognitive assessment)를 통한 인지기능 검사
  - BDI(Becks' depression inventory)를 통한 우울증 평가
  - 숫자 바로/거꾸로 외우기 검사
  - 전산화 신경인지기능 검사
  - 간단 신체 수행 검사(Short Physical Performance Battery)
⑤ 병용약물조사를 실시한다.

⑥ 지난 방문 이후 새롭게 발생한 이상반응을 확인하고, 기 발생한 이상반응에 대해 추적관찰 한다.

⑦ 연구를 종료하고 필요시 재방문하도록 안내한다.

11.4.1.7 예정되지 않은 방문 (Unscheduled Visit)

이상반응, 병용약물의 변화, 중도탈락 여부, 임상시험 중 실시된 측정 결과에 따른 의학적 처치 필요 등의 사유가 있는 대상자가 예정된 날짜가 아닌 날에 방문하였다면, 관련 내용을 근거 문서에 가능한 상세하게 기록해둔다. 또한, 예정되지 않은 방문으로 인해 계획된 시험 진행 일정이 변경되어서는 안된다. 이때, 임상시험 시작 전부터 계획되어 있던 대상질환 외 기저질환, 검진 등으로 인한 병원의 방문은 예정되지 않은 방문에 해당되지 않는다.
예정되지 않은 방문은 예정된 방문 외에 대상자의 요청 또는 연구자의 판단에 따라 필요하다고 판단될 때 수시로 이루어질 수 있다. 예정되지 않은 방문에 실시되는 검사는 가급적 방문 4 에 해당하는 모든 검사를 실시하도록 하지만, 강제적으로 제한을 두지 않으며 시험자의 판단 하에 대상자에게 필요한 추가의 적절한 검사를 시행하도록 한다.

11.4.2 관찰항목

11.4.2.1 타 임상시험 참여 여부

연구대상자가 타 임상시험 등에 중복하여 참여하는 것을 방지하기 위하여 본인 확인 절차를 마련하고, 스크리닝 방문을 포함하여 매 방문시 타 임상시험 참여 여부를 확인하도록 한다. 임상시험 참여 전 30일 이내에 다른 연구에 참여하였는지 확인하여 참여한 경우 연구에 등록할 수 없다.

11.4.2.2 인구학적 조사

생년월일, 나이, 성별, 키, 몸무게, BMI, 최종학력, 직업군 등을 조사한다. 생년월일은 주민등록증 등에 기재된 공인 양력 날짜로 기입하며, 나이는 서면동의일 기준 만으로 계산한다. 최종학력은 초등학교 미만, 초등학교, 중학교, 고등학교, 대학 이상에서 선택하도록 한다. 직업군은 육체 노동, 비육체 노동, 기타(무직 등)에서 선택하도록 한다.

11.4.2.3 과거력 및 현병력 조사

연구대상자가 과거에 경험하였거나 현재 진행중인 임상적으로 유의한 의학적 상태 또는 비정상 상태를 조사하여, 진단명, 시작일, 종료일, 스크리닝시 지속여부, 소견을 기록한다. 또한 COVID-19 확진일에 대하여 수집한다. 특별히 제외기준에 해당하는 연하장애, 임상적으로 심각한 소화장애, 갈락토오스 불내성, Lapp 유당분해효소 결핍증, 포도당-갈락토오스 흡수장애 등의 유전적인 문제 등 약물의 복용이나 흡수에 영향을 줄 수 있는 질환을 가지고 있는지의 여부,

심근경색이나 심부전과 같은 심장질환의 과거력 있는 지 여부, 연구약에 대한 알러지 과거력이 있는지 여부, 간질환이나 신장질환의 병력이 있는지 여부, 당뇨 여부 등에 대해서 조사한다.

11.4.2.4 피로에 대한 과거력 조사

연구대상자가 피로를 유발할 수 있는 기질적인 질환(암, 수면장애, 만성 간염, 간경화, 만성 신부전, 결핵, 천식, 다발성경화증)을 과거에 경험하였거나 현재 진행중인 임상적으로 유의한 의학적 상태에 있는지 조사한다.

11.4.2.5 현재 피로에 대한 증상의 평가

다음과 같은 피로의 증상을 가지고 있는지 평가하며, 다음 기준을 모두 만족해야 피로를 가지고 있는 것으로 확정할 수 있다[21, 22]

- 지속적으로 육체적 활동 혹은 정신적 활동에 의해 심해지는 피로를 경험함
- 일상생활활동, 직업활동, 사회적활동 및 여가활동 등 4 가지 활동영역에서 최소 3 개의 영역에서 어려움을 겪음
- 피로를 야기하는 육체적, 정신적 질환에 대해서 진단받지 않음
- 개운치 않은 수면

11.4.2.6 COVID-19 확진에 대한 조사

COVID-19 확진여부를 판단하기 위해서 대상자가 발부하여 제출한 질병관리청의 확진관련 안내 문자메시지, 코로나관련 진단서 혹은 소견서, 입원관련 의무기록, 확진당시 PCR 검사기록 등을 확인한다. COVID-19관련해서 백신 접종력(백신접종여부, 백신종류, 접종일, 접종일자, 이상반응 유무), 코로나가 확진된 시기와 다음 자료를 수집한다

- COVID-19 확진 및 치료 당시의 병력 문진
- 조기경고점수(EWS; early warning score)

EWS는 연구대상자의 의무기록이 확인 가능한 경우 의무기록을 통해 추출한다. 만일 확인이 불가능한 경우에는 대상자의 기억에 의거하여 수집한다. EWS의 평가는 SpO2, suppl Oxygen, Heart rate, Systolic BP, Respiratory rate, Body Temp, CNS level을 통해 평가하며 다음과 같은 자료를 분석한다. The National Early Warning Score (NEWS; mild < 5, severe ≥ 5)에 따라 중증 또는 경증 COVID-19로 구분한다.

1) EWS 평가 일자(증상이 가장 심한 날), 호흡률(respiratory rate), 산소포화도 (oxygen saturation), 산소보충제(oxygen supplement), 체온(body temperature), 수축기혈압 (systolic blood pressure), 심박수(heart rate), 그리고 의식 (consciousness)
2) 다음 표에 의거하여 EWS를 계산하여 CRF에 기록한다.

변수	3	2	1	0	1	2	3
산소포화도	91이하	92-93	94-95	96이상			
산소투여여부	예	예	아니오	아니오			
맥박	40이하		41-50	51-90	91-110	111-130	130이상
수축기혈압	90이하	91-100	101-110	111-219			220이상
호흡수	8이하		9-11	12-20		21-24	25이상
체온	35.0이하		35.1-36.0	36.1-38.0	38.1-39.0	39.1이상	
의식수준				정상			이상

11.4.2.7 복용약물조사

현재 복용 중인 약물에 대해 조사한다. 조사항목은 약물명, 용법, 용량, 단위, 투여경로, 복용시작일, 복용종료일, 지속여부, 복용목적 등이다.

11.4.2.8 Vital sign 측정

혈압(수축기, 이완기), 체온, 호흡수, 맥박수를 측정한다. 모두 연구대상자가 앉은 자세로 5분 휴식 후 측정한다.

11.4.2.9 심전도검사

급성심근경색 및 심실세동 등의 중증심장질환의 여부를 평가한다.

11.4.2.10 실험실적 검사

실험실적 검사를 실시하여, 다른 질환의 여부에 대한 기초적인 검진을 실시한다. 구체적인 사항은 "관찰항목과 관찰검사방법"의 "기타 관찰항목" 중 "임상검사항목" 참조한다.

11.4.2.11 면역검사 및 대사체분석

1. 검체 채취 및 보관

1.1 채혈

채혈은 임상시험검체분석기관으로 지정된 경희의료원내 채혈실에 의뢰하여 진행하며, 피험자의 동의를 얻어 채혈 1회당 25ml 채취한다(5ml EDTA튜브 4 개, 5ml 일반튜브 1 개). 검체 익명화 처리 후 얼음을 채운 보냉박스에 담아 신속하게 scRNA-seq 분석기관인 ㈜로킷제노믹스로 이송하여 5ml EDTA 튜브 2 개에 채취한 혈액은 적혈구 용해법을 이용하여 말초단일세포를 분리하고, 나머지 5ml EDTA 튜브 2 개에 채취한 혈액은 원심분리법을 이용하여 말초단일세포를 분리한다. 5ml 일반튜브 1 개에 채취한 혈액은 혈청을 분리한다. 전처리가 완료된 인체유래물은 ㈜로킷제노믹스에 임시로 보관한다.

임시보관 사업장 주소: ㈜로킷제노믹스, 서울시 금천구 디지털로 10 길 9, 가산하이힐 12 층

보관 조건: 말초혈액단일세포 (-190℃, 액체질소), 혈청(-80℃, 초저온냉동고)

㈜로킷제노믹스에 임시로 보관한 인체유래물 전량은 특수배송을 이용하여 한국한의학연구원으로 이송하여 인체유래물 연구 동의서의 보존기간에 의거하여 보관한다. 경희대학교 한방병원에서 수집된 개인정보는 익명화 처리 후 한국한의학연구원으로 제공되고, 수집된 개인정보는 인체유래물 분석연구에 한정하여 활용하며, 연구보안규정을 준수하여 보관한다. 인체유래물의 폐기는 인체유래물 연구 동의서의 보존기간에 의거하여, 질병관리청 의료폐기물처리매뉴얼에 명시된 폐기물처리방법에 따라 멸균처리 한다.

사업장 주소: 한국한의학연구원 구암관 201 호 감염질환 연구실, 대전 유성구 유성대로 1672

보관 조건: 말초혈액단일세포 (-190℃, 액체질소), 혈청(-80℃, 초저온냉동고)

인체유래물의 보존기간은 연구대상자가 자의에 의해서 작성한 연구동의서의 보존기간 동안 보존하며, 인체유래물의 2 차 활용은 하지 않기 때문에 2 차 사용목적으로 제공가능한지에 대한 동의는 별도로 받지 않으며, 2 차 사용 목적의 개인정보 또한 첨부되지 않는다.

1.2 혈액 단일세포 및 혈청 분리 및 보관

혈액 단일세포는 말초정맥혈에서 적혈구 용해(RBC Lysis) 또는 원심분리법 (density gradient centrifugation)으로 분리한다. 사용 시까지 동결배지에서 동결하여 액체질소에 보관하고, 해동 시 모든 샘플에서 약 90% 이상의 생존율을 보유하고 있음을 확인한다. 혈청은 채혈 후 바로 clotting을 위하여 30-60분 간 방치하고, 원심분리하여 획득한 후 cryovials에 0.5ml씩 분주하여 각 혈청 당 약 2-3개의 cryovials을 만든 후 freezer box에 넣어 각 지표의 측정 전까지 -80℃의 초저온 냉동고에 보관한다.

① 면역학적 검사

면역학적검사항목, 검채채취 및 보관, 이송방법, 검체분석하는 주체 및 분석방법 등

1. 면역학적 검사를 위하여 관찰하고자 하는 항목

- Single-cell RNA sequencing (scRNA-seq) 을 이용한 면역 유전자 발현 분석

- 유세포분석 (Flow cytometry analysis)를 이용한 SARS-CoV-2 특이 항체 immunophenotyping 분석
- 혈청 내 사이토카인 수준 정량분석
- 혈청 내 SARS-CoV-2 특이 항체 정량분석
- 혈청 내 SARS-CoV-2 중화항체 역가 정량분석
- 면역학적 검사항목 측정시기

한약제제 투여 직전, 투여 종료 직후, 종료 후 12주, 종료 후 24주 (총 4회)

2. 실험방법

2.1 단일세포 전사체분석 (single-cell RNA sequencing, scRNA-seq) 을 이용한 면역관련 유전자 발현 비교 분석

피험자에서 얻은 PBMC의 전사체 분석에 의하여 면역세포 (B cell, plasma 세포, NK 세포, CD4 T세포, CD8 T 세포, myeloid 세포, epithelial 세포) 등의 분포 및 발현양상 분석 후, 변화가 있는 특정 면역세포 내 특이적인 전사체 발현 양상 (사이토카인 발현, transcription factor, signal pathway 등)을 조사하여 피험자의 한약제제 복용 전후 전사체의 변화를 파악하고자 한다. scRNA-seq 분석 관련 검체 이송 및 데이터 분석까지의 검체 보관은 ROKIT Genomics社가 수행한다. scRNA-seq libraries는 Chromium Single Cell 3′ Library & Gel Bead Kit v3 (10×Genomics)로 제작한다. 수천개의 세포를 nanoliter-scale droplets로 분리하고, 각 droplet에서 역전사로 complementary DNA (cDNA)를 제작하여 세포 barcoding 서열과 고유분자표지 (unique molecular identifiers, UMI)를 각 cDNA 분자에 부여한다. 라이브러리를 제작하고 the Nextseq 550 또는 Novaseq 6000 platform (Illumina)을 이용하여 염기서열분석하여 데이터를 분석한다. 한약제제 복용 전후 면역관련 유전자에 관하여 발현차이가 나는 RNA를 대상으로 Barplot-Significant mRNAs, Scatter plot, Volcano plot, Hierarchical clustering heatmap 등으로 표현하고 target mRNA DB를 사용하여 후보 mRNA 들을 정리한다. 데이터 분석 완료 후 잔존하는 검체는 한국한의학연구원으로 이송하여 보관한다. 후보 mRNA의 재확인을 위하여 선별된 significant maker에 대해 개별 샘플에서 real-time qPCR을 진행한다.

2.2. 유세포분석 (Flow cytometry analysis, FACS)를 이용한 면역표현형 (immunophenotyping) 비교 분석

한국한의학연구원에서 동결 보존 된 단일세포를 해동하고, 죽은 세포는 Live/Dead Fixable Cell Stain kit를 이용하여 염색하여 확인한다. 한약제제 복용 전후의 면역세포의 분포 및 활성 마커의 발현을 비교 분석을 위하여, anti-CD3 (BV605; BD Biosciences), anti-CD4 (BV510; BD

Biosciences), anti-CD8 (BV421; BD Biosciences), anti-CD19 (Alexa Fluor 700; BD Biosciences) 등을 포함하는 fluorochrome-conjugated antibodies로 염색하여 FACS로 분석한다.

2.3 혈청 내 사이토카인, SARS-CoV-2 특이 항체, 중화항체 역가 정량분석

혈청 내 pro-inflammatory cytokine (IL-8, IL-1beta, IL-6, IL-10, TNF and IL-12p70) 등의 수준 정보는 염증반응의 진행단계 및 양상, 과염증반응에 의한 장기부전 등의 후유증 정도를 평가할 수 있는 지표로 피험자의 한약제제 복용 전후의 사이토카인 발현 변화를 조사하고자 한다.

- 한약제제 복용 전후의 혈청 내 사이토카인 수준의 비교는 한국한의학연구원에서 Cytometric Bead Array 키트 (BD Biosciences)를 이용하여 FACS로 비교 분석한다.
- 한약제제 복용 전후의 혈청내 SARS-COV-2 특이항체는 SARS-CoV-2 S 단백질에 특이적인 IgG 또는 IgM을 측정하기 위하여, 한국한의학연구원에서 SARS-CoV-2 S 단백질 (Sino Biological)을 96웰 플레이트에 코팅하고 혈청샘플을 반응시켜 horseradish peroxidase (HRP)-conjugated anti-human IgG, IgG1, IgG2a, IgG2B 또는 IgM 항체 (Jackson immunoresearch)로 검출하여 분석한다.
- 혈청내 SARS-COV-2중화항체 역가는 한국한의학연구원에서 SARS-CoV-2 Neutralization Detection Kit (GenScript)를 이용한 분석을 시행하고, 충북대학교 생물안전3등급 연구시설에서 플라그 감소 중화항체 검사 (plaque reduction neutralization test)를 이용하여 분석한다.

① 대사체 분석

대사체(metabolites)는 대사과정에서 생성되는 다양한 생성물을 지칭하는 것으로 생물의 직접 대사산물뿐만 아니라 외인적 요소(감염, 장내세균등)로부터 생산되기도 하며 생리학, 생화학적으로 광범위한 영향을 미치는 것으로 알려져 있다. 본 연구에서는 LC-MS와 GC-MS 분석 방법을 이용하여 혈청 내 일차 및 이차 대사산물 등의 대사체 프로파일을 얻어 한약제제 복용 전후의 대사 변화를 파악하고자 한다.

1. 질량분석기 기반 혈액 metabolomics 분석 개요

- 대사체(metabolites)는 대사과정에서 생성되는 다양한 생성물을 지칭하는 것으로 생물의 직접 대사산물뿐만 아니라 외인적 요소(감염, 장내세균등)로부터 생산되기도 하며 생리학, 생화학적으로 광범위한 영향을 미침. 대사체는 직접적인 작용 기작의 조절에 관여하고, 또한 박테리아의 대사 기전을 조절함으로써 항상성 유지에 기여 하기도 함. 따라서 대사체의 작용 기전에 대한 정보를 기존의 생물학적 정보와 통합/해석하여 새로운 분석적 접근법을 제시한다는 것은 기초 생물학적 작용 및 질병을 이해하는데 매우 중요함. 분해능 질량분석기는 대사체 전체를 한꺼번에 분석하는 매우 효율적인 기기로 대부분은 가스크로마토그래피(GC), 액체크로마토그래피(LC) 등의 분리분석법을 결합한 방법이 사용되고 있음.

2. 대사체 분석을 통하여 관찰하고자 하는 항목

- LC-MS, GC-MS에 따라서 분석 되는 대사체의 종류가 아래의 그림과 같이 다르게 분포하고 있으며, 본 연구에서는 LC-MS와 GC-MS 분석 방법을 모두 선택하여 한국한의학연구원에서 혈청 내 Alkaloids and derivatives, Benzenoids, Homogeneous non-metal compounds, Lipids and lipid-like molecules, Nucleosides, nucleotides, and analogues, Organic acids and derivatives, Organic nitrogen compounds, Organic oxygen compounds, Organohalogen compounds, Organoheterocyclic compounds, Phenylpropanoids and polyketides 류를 포함한 일차 및 이차 대사산물 등의 대사체 프로파일을 얻어 한약제제 복용 전후의 대사 변화를 파악하고자 함.

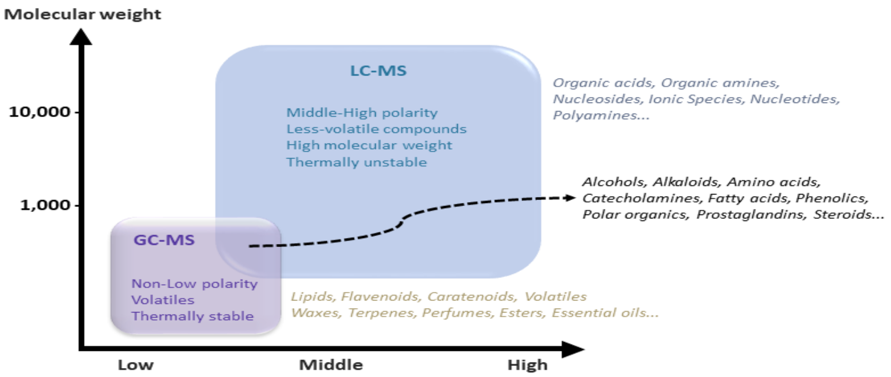

검체	분석 방법	분석 수행기관	분석 목적
혈액/단일세포	scRNA-Seq	ROKIT Genomics社	한약제제 복용 전후 면역관련 전사체 변화 확인
	Real-time qPCR	한국한의학연구원	한약제제 복용 전후 면역관련 전사체 변화 재확인
	유세포분석 (FACS)을 이용한 Immunophenotyping	한국한의학연구원	한약제제 복용 전후 면역세포 분포 변화 확인
혈액/혈청	사이토카인 분석(CBA)	한국한의학연구원	한약제제 복용 전후 혈청내 사이토카인 수준변화 확인
	SARS-CoV-2 특이항체 분석	한국한의학연구원	한약제제 복용 전후 SARS-CoV-2 특이 항체수준 변화 확인
	SARS-CoV-2 중화항체분석	한국한의학연구원/ 충북대학교	한약제제 복용 전후 SARS-CoV-2 중화 항체수준 변화 확인
	대사체 분석	한국한의학연구원	한약제제 복용 전후

			대사체 변화 확인

<Table> 인체유래물 검체 종류, 분석 방법, 분석 수행기관 및 분석 목적

11.4.2.12 임신, 수유 여부 평가

가임기 여성에 한해 혈청 또는 뇨 검사(스틱)로 β-hCG 검사를 실시한다. 한약제제 투여 전 28 일 이내 검사 결과가 있는 경우 생략할 수 있다. 폐경(최소 1 년 이상 무월경), 자궁적출술, 양측난소(또는 난관) 절제술 실시자는 검사에서 제외된다. 임신가능성과 수유 여부에 대해서는 구두로 확인한다.

11.4.2.13 FSS (Fatigue severity scale)

FSS 는 지난 1 주 동안의 피로를 평가하는 지표로, 연구책임자 또는 담당자의 지시 하에 대상자가 직접 설문지를 이용하여 평가한다. 대상자가 설문지를 평가하기 전, 시험담당자는 설문지 평가방법을 대상자에게 설명한다. 총 9 개의 문항으로 구성되며, 각 문항은 1 점에서 7 점으로 평가하고, 증상이 심할수록 점수가 높다. FSS 점수는 9 개 항목의 평균으로 계산하며, 만성 코로나 19 증후군 증후군의 진료지침과 임상연구 결과에 근거하여 4 점이 넘는 경우 상당한 피로증상을 가진 사람으로 판단하는 기준으로 삼는다[23, 24]

11.4.2.14 피로 증상이 4 주이상 지속되었는지 여부 조사

피로 증상의 지속기간에 대하여 조사하고, 4 주이상 지속인지 확인한다.

11.4.2.15 피로의 0-100 VAS(Visual analogue scale)

지난 일주일동안 평균적으로 느끼는 피로에 대해서 대상자가 체크하게 하도록 한다. 0-100mm VAS 선에서 0 은 증상 없음, 100 은 증상이 매우 심함을 의미한다.

11.4.2.16 ChFS(Chalder Fatigue Scale)

Chalder Fatigue Scale 의 한국어판을 참고하여 평가한다.. 점수는 총점과 physical(1-7 번)/mental health(8-11 번) 로 나누어 sub-scale 을 평가한다[25].

11.4.2.17 EQ-5D-5L

EQ-5D 는 5 가지 차원에서 5 가지 수준으로 건강상태 프로파일을 분류한다. 5 가지 차원은 '운동능력(mobility)', '자기관리(self care)', '일상활동(usual activity)', '통증/불편(pain/discomfort)', '불안/우울(anxiety/depression)'이며, 5 가지 수준은 1= 문제가 없는 경우, 5=중증의 문제가 있는 경우이다.

삶의 질 점수는 가중치를 추정한 아래 산출식을 이용하여 계산한다[26].

Quality weight=1-(0.096 + 0.046 × M2 + 0.058 × M3 + 0.133 × M4 + 0.251 × M5 +
0.032 × S2 + 0.050 × S3 + 0.078 × S4 + 0.122 × S5 +
0.021 × U2 +0.051 × U3 + 0.100 × U4 + 0.175 × U5 +
0.042 × P2 + 0.053 × P3 + 0.166 × P4 + 0.207 × P5 +
0.033 × A2 + 0.046 × A3 + 0.102 × A4 + 0.137 × A5 + 0.078 × N4)

(N4: 수준 4 이상이 하나라도 있는 경우 1, 아니면 0)

M=Mobility, S=Self care, U=Usual activities, P=Pain/discomfort, A=Anxiety/depression

11.4.2.18 PSQI-K (Korean version of Pittsburgh Sleep Quality Index)

PSQI-K 는 19 개의 대상자 질문지와 5 개의 룸메이트 또는 같은 잠자리를 사용하는 파트너의 질문지로 구성되어 있다. 19 개의 대상자 질문지 점수만 global score 로 계산된다. 19 개의 대상자 질문지 답변은 7 개의 component 점수로 평가되고, 7 개의 component 점수의 합산으로 global score 를 계산된다. Global score 의 총점은 0~21 점 이다.

11.4.2.19 K-MOCA(Korean-Montreal cognitive assessment)

K-WAIS-IV 가 인지기능을 종합적으로 평가하는데 좋은 도구임에도 불구하고, 1 회 평가에 1 시간 이상이 소요되어, 다회 반복평가의 어려움으로 인해 인지기능을 평가하기 위하여 별도의 평가도구가 필요하여, 치매의 평가도구인 MMSE 보다 인지기능의 저하에 민감도가 높으면서 간단한 평가도구인 K-MOCA 평가를 실시한다. K-MOCA 는 시각공간력, 실행력(3 점), 언어능력-어휘력(3 점), 언어능력-문장력(3 점), 주의집중능력(6 점), 수상력(2 점), 지남력(6 점)으로 구성된다. 기억력은 항목은 있으나 점수에 산정되지 않으며, 총 점수는 30 점 만점이고, 23 점이상이면 인지기능이 정상으로 판단할 수 있다[30].

11.4.2.20 Becks' depression inventory (BDI)

COVID-19 확진 혹은 완치 후 발생한 우울증상의 유무와 중증도를 평가하기 위해 실시한다. 우울증의 인지적, 정서적, 동기적, 신체적 증상의 4 개 영역, 총 21 개의 문항으로 구성되어 있으며, 문항 점수의 총합은 0-63 점으로 점수가 높을수록 우울증의 증상이 심함을 의미한다 [31][32].

11.4.2.21 숫자 바로/거꾸로 외우기 검사(DF, DB 검사)

Wechsler 지능척도는 임상에서 가장 많이 활용되는 지능검사도구이며 국내에서는 K-WAIS 가 널리 통용되고 있다. 이 중 숫자외우기 소검사는 인지기능저하를 평가하기 위한 단기기억과

작업기억 등의 기능을 평가하는 간편한 도구로 인식되고 있다. 숫자외우기 소검사는 바로 따라 외우기(DF)를 실시한 후 거꾸로 따라 외우기(DB)를 실시한다. DF 에서는 검사자가 불러준 숫자를 대상자가 똑같이 대답하도록 하고, DB 에서는 거꾸로 대답하도록 하고, 1 초에 1 개 정도의 속도로 숫자를 불러준다. DF 는 3 자리수에서 9 자리까지 불러주고, DB 는 2 자리수에서 8 자리수까지 불러주며, 응답이 맞을 경우 O, 틀릴 경우 X 표기를 하고, 연속으로 두번 틀릴 경우 검사를 중지한다.

점수는 대상자가 바르게 말한 가장 큰 숫자의 자리수가 되고, DB 점수는 대상자가 정확하게 거꾸로 외운 가장 큰 숫자의 자리수가 된다. DF 점수, DB 점수, DF-DB 점수를 구하며, DF, DB 점수는 높을수록 단기기억력이 좋다고 판단할 수 있으며, DF-DB 점수가 5 이상인 경우 작업기억을 비정상으로 판단할 수 있다[44].

11.4.2.22 전산화 신경인지기능 검사 (CNT40)

전산기기를 활용한 17종의 검사를 통해 대상자의 신경인지기능을 평가한다. 해당 검사는 언어능력, 기억력, 주의력, 계획적 사고능력, 운동능력과 같은 다양한 신경인지 기능을 평가하는 도구로, 만성 코로나19증후군환자의 인지기능저하를 평가하는 검사법이다. 본 연구에서는 국내에서 개발되어 임상에서 활용되고 있는 CNT40을 이용하며, 이 중 주의력과 고위인지기능을 평가하는 5가지 항목을 분석한다[33, 34].

- Auditory C.P.T (언어 지속 검사) : 청각 자극을 주고 피검사자가 정확히 반응하는지 측정하는 검사, 정반응수의 측정값을 분석한다.
- Auditory Controlled C.P.T (조건 언어 지속검사) : 동일한 연속 청각 자극에 피검사자가 정확히 반응하는지 측정하는 검사, 정반응수의 측정값을 분석한다.
- Visual C.P.T (시각 지속 검사) : 시각 자극을 주고 피검사자가 정확히 반응하는지 측정하는 검사, 정반응수의 측정값을 분석한다.
- Visual Controlled C.P.T (조건 시각 지속검사) : 동일한 연속 시각 자극에 피검사자가 정확히 반응하는지 측정하는 검사, 정반응수의 측정값을 분석한다.
- Trail Making Test (선로잇기 검사) : 숫자와 문자를 순서대로 연결하도록 하고 반응시간을 측정하는 검사, 두가지 측정변수의 소요시간을 분석한다.

CNT 40(주 맥스메디카) 장비	검사결과 예시

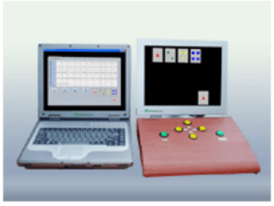

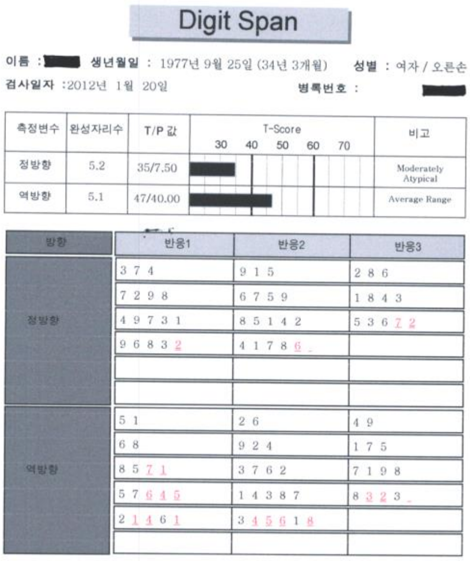

Digit Span

이름 : [redacted] 생년월일 : 1977년 9월 25일 (34년 3개월) 성별 : 여자 / 오른손
검사일자 :2012년 1월 20일 병록번호 : [redacted]

측정변수	완성자리수	T/P 값	T-Score 30 40 50 60 70	비고
정방향	5.2	35/7.50		Moderately Atypical
역방향	5.1	47/40.00		Average Range

방향	반응1	반응2	반응3
정방향	3 7 4	9 1 5	2 8 6
	7 2 9 8	6 7 5 9	1 8 4 3
	4 9 7 3 1	8 5 1 4 2	5 3 6 7 2
	9 6 8 3 2	4 1 7 8 6	
역방향	5 1	2 6	4 9
	6 8	9 2 4	1 7 5
	8 5 7 1	3 7 6 2	7 1 9 8
	5 7 6 4 5	1 4 3 8 7	8 3 2 3
	2 1 4 6 1	3 4 5 6 1 8	

11.4.2.23 간단신체수행검사 (Short Physical Performance Battery)

간단신체수행검사(SPPB)는 노인 혹은 병후 허약자의 신체 기능을 평가하는 검사로, 정적 균형 검사, 보행속도 검사, 의자에서 일어나기 검사 등 3 가지 검사로 구성되어 있다. 만성 코로나 19 증후군 환자의 신체기능 감소 및 기능회복을 평가하기 위해 시행한다. 구체적인 방법은 다음과 같다.

정적 균형 검사: 일반 자세, 반일렬 자세, 일렬 자세 3 가지 자세에서의 균형을 평가한다. 각 자세에서 10 초 동안 균형을 잃지 않고 유지할 수 있는지 확인한다. 총 4 점 만점으로, 일반자세와 반일렬 자세 완수 시 각 1 점씩, 일렬자세 유지 시 10 초는 2 점, 3-9.99 초는 1 점, 3 초 미만은 0 점을 부여한다.

보행속도 검사: 4 미터 거리를 걷는 데 소요되는 시간을 측정한다. 총 4 점 만점으로, 4.82 초 미만 소요 시 4 점, 4.82-6.20 초는 3 점, 6.21-8.70 초는 2 점, 8.70 초 초과 소요 시 1 점, 걸음이 불가능한 경우 0 점을 부여한다.

의자에서 일어나기 검사: 팔의 힘을 사용하지 않고 다리 힘만으로 의자에서 5 회 일어나는 데 걸리는 시간을 측정한다. 총 4 점 만점으로, 11.20 초 미만 소요 시 4 점, 11.20-13.69 초는 3 점, 13.70-16.69 초는 2 점, 16.70 초 이상 소요 시 1 점, 5 회 일어서는 동작을 완수하지 못하거나 소요 시간이 60 초가 초과된 경우 0 점을 받게 된다.

본 연구에서는 정확한 측정을 위해 안단테핏(Andante fit)이라는 시스템을 이용한다.
안단테핏은 보행속도 측정기기, 일어서기 측정기기, 정적균형능력 측정기기 등으로 구성되며, 2-3 분의 시간이 소요되고, 측정 완료 후 각 항목의 점수와 종합점수가 자동으로 도출된다[35].

안단테핏의 구성	검사결과 예시
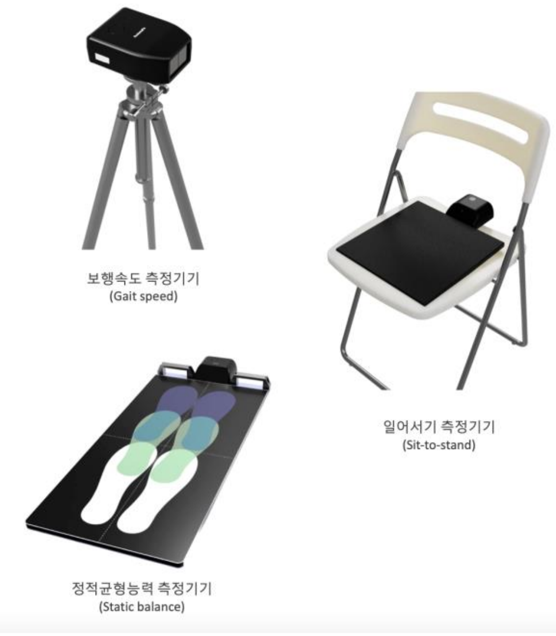	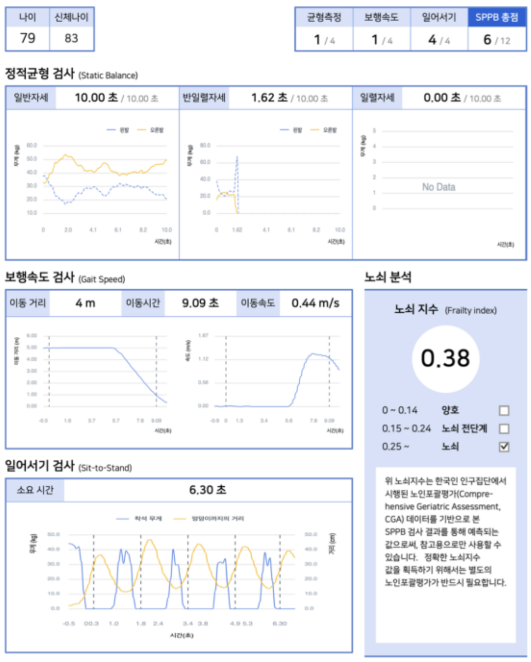

11.4.2.24 복약 순응도

각 방문마다 반납된 약의 개수 및 복용한 약의 개수를 조사하여 기록한다. 만일 처방된 약 대비 반납된 약의 개수와 복용한 약의 개수가 일치하지 않는 경우 이에 대한 사유를 증례기록서에 기록하도록 한다. 복약순응도는 다음과 같이 평가한다.

$$\text{복약 순응도(\%)} = \frac{\text{실제 복용한 약의 개수}}{\text{해당 기간 동안 복용해야 하는 약의 개수*}} \times 100$$

* 해당 기간 동안에 복용해야 하는 약의 개수 = 최초로 의약품을 투여하는 시점부터 의약품을 반납하는 방문 직전까지 복용하는 의약품 개수(포)

최종 복약 순응도는 방문 4 에 평가되며, 방문 2, 방문 3, 방문 4 에 수집된 실제 복용한 한약제제의 합과 해당 기간 동안 복용해야 하는 임상시험용의약품의 개수를 합산하여 계산한다.

11.4.2.25 Feasibility 평가

연구설계의 feasibility 평가를 위해 연구기간동안 대상자의 모집율(전체, 기관별), 대상자의 중도탈락율(전체, 기관별), 중도탈락 이유 등에 대한 자료를 수집한다.

11.4.2.26 이상반응 확인

연구 대상자에게 발생한 이상반응에 대한 정보는 연구 대상자 또는 대리인이 수시로 자발적인 보고를 하도록 하며, 연구책임자 또는 연구담당자의 면담 및 문진 등 진료를 통하여 확인한다. 이상반응 조사에는 발현일 및 소실일, 이상반응의 정도 및 결과, 취해진 조치 및 한약제제투여와의 인과관계, 이상반응에 대한 치료 여부 및 내용 등이 포함된다.

-자·타각적 증상에 대한 점검

자·타각적 증상은 시험자의 진료를 통하여 이상반응 발현 정도를 조사하고, 평가기준에 따라 발현 정도를 기록하며, 약제 투여 및 한약제제와의 인과관계도 평가한다.

-실험실적 검사, 활력징후 등 측정된 검사에 대한 점검

실험실적 검사, 활력징후 등 검사 결과에서 임상적으로 유의한 비정상적 변화는 증상호전 때까지 추적검사를 실시하도록 한다. 또한 검사 시 검사내용에 지장을 줄 수 있는 요인이 있었는지 면담을 통하여 기록한다.

12. 임상시험용의약품의 사용상 주의사항

12.2 CV1(진경옥고)과 P(위약)

**1. 다음과 같은 사람은 이 약을 복용하지 말 것.**<br>3 개월 미만의 영아(젖먹이, 갓난아기)<br>**2. 다음과 같은 사람은 이 약을 복용하기 전에 의사, 한의사, 치과의사, 약사, 한약사와 상의할 것.**<br>1) 다른 약물을 투여 받고 있는 환자<br>2) 고령자(노인)(일반적으로 고령자(노인)는 생리기능이 저하되어 있으므로 감량(줄임)하는 등 주의할 것.)<br>3) 현저하게 위장이 허약한 환자(식욕부진, 위부불쾌감, 구역, 구토, 설사 등이 나타날 수 있다.)<br>4) 식욕부진, 구역, 구토의 증상이 있는 환자(증상이 악화될 수 있다.)<br>5) 1 세 이하의 영아(젖먹이, 갓난아기)

3. 다음과 같은 경우 이 약의 복용을 즉각 중지하고 의사, 한의사, 치과의사, 약사, 한약사와 상의할

것. 상담 시 가능한 한 이 첨부문서를 소지할 것.

1) 이 약의 복용에 의해 다음의 증상이 나타난 경우

(1) 피부 : 발진, 두드러기 등

(2) 소화기계 : 식욕부진, 위부불쾌감, 구역, 구토, 설사 등

2) 수일간 복용하여도 증상의 개선이 없을 경우

4. 기타 이 약의 복용시 주의할 사항

1) 정해진 용법·용량을 잘 지킬 것.

2) 다른 한약(생약)제제 등과 함께 복용할 경우에는 함유 생약의 중복에 주의할 것.

3) 어린이에게 복용시킬 경우에는 보호자의 지도·감독 하에 복용시킬 것.

4) 온수나 냉수에 담근 수저를 약병에 넣지 말 것(엑스제에 한함).

5) 약수저를 재 사용시 물기 없이 깨끗이 닦아서 쓸 것(엑스제에 한함).

5. 저장상의 주의사항

1) 직사광선을 피하고 되도록 습기가 적은 서늘한 곳에 보관할 것(사용 후 반드시 밀폐 보관할 것.).

2) 어린이의 손이 닿지 않는 곳에 보관할 것.

3) 의약품을 원래 용기에서 꺼내어 다른 용기에 보관하는 것은 의약품 오용(잘못 사용)에 의한 사고 발생이나 의약품 품질 저하의 원인이 될 수 있으므로 원래의 용기에 넣고 꼭 닫아 보관할 것.

13. 연구대상자의 중지 및 탈락기준, 임상시험 종료기준

본 임상시험에 참여하는 모든 대상자의 연구참여 완료여부를 기록하고, 임상시험약의 투여나 관찰이 중단된 경우에는 그 이유를 기록한다. 연구대상자가 참여를 중단할 수 있는 경우는 다음과 같다.

① 한약제제에 대하여 급성반응 (알러지, 과민반응 등)을 보이는 경우
② 선정/제외기준에 적합하지 않는 연구대상자가 참여한 경우
③ 예측되지 않은 질환이나 사고로 연구자가 투여 및 관찰이 불가능하다고 판단되는 경우
④ "중대한 이상반응"이나 "약물이상반응"이 심각하여 지속적인 투여 및 관찰이 적절하지 않다고 연구자가 판단하는 경우
⑤ 연구대상자가 임신한 경우

⑥ 연구대상자 또는 대리인이 참여의 중단을 요구하는 경우 (동의 철회)
⑦ 기타 연구자의 판단에 의해 연구의 진행이 적합하지 못하다고 판단되는 경우

본 임상시험의 종료기준은 다음과 같다.

임상시험은 목표 대상자 수가 모두 등록되고 데이터의 완결성이 확보되면 종료된다. 마지막 연구대상자의 추적관찰이 완료되면, 시험책임자는 종료 일자를 기록하고 데이터를 분석하여 분석을 실시한다. 개별 연구대상자의 임상시험은 계획된 방문 및 평가를 모두 완료하고, 계획서에 명시된 과정을 수행한 경우 종료된다.

14. 통계 분석 방법

14.1 결과분석의 일반적 원칙

유효성 평가에 대한 자료는 FAS 를 주 분석으로 한다. 안전성 평가에 대한 자료는 safety set 에서 평가한다. 별도 정의하지 않은 모든 통계 검정은 양측 검정, 5% 유의수준에서 검정한다.
유효성 평가 시 FAS 에 대해 결측치가 발생한 경우, Last Observation Carried Forward(이하, 'LOCF') 방법을 적용하여 통계분석을 실시하고, 그 외에는 원래 자료대로 통계분석을 실시한다.

Safety Set(SS): 임상시험약을 1 회 이상 투여한 대상자 중, 안전성 관련 추적 관찰이 한번이라도 시행된 대상자 집단

Full Analysis Set(FAS): ITT 원칙에 따르며, 임상시험약을 투여 후 주요 유효성 평가변수에 대한 측정이 1 회 이상 이루어진 대상자 집단

유효성 평가 외에 Feasibility 평가를 위한 대상자 모집율, 중도탈락율, 중도탈락 이유 등에 대한 분석의 경우 대상자 모집율은 총 연구대상자를 총 스크리닝 대상자 수로 나누어 계산하며(%), 중도탈락율은 전체 및 각 한약제제 처방군의 중도탈락율과 이유에 대하여 빈도를 제시하는 등 기술적인 분석을 시행한다.

유효성 평가변수에 대한 분석을 위해 두 군간 차이가 있는지 확인하기 위해, 배정된 군을 요인, baseline 값을 공변량으로 하는 ANCOVA 를 실시하여 군간 차이를 검정한다(양측 $p<0.05$). 만약 공분산분석의 가정에 대한 검정을 실시하여 자료의 정규성, 등분산성 등의 기본 가정이 위배되고 outlier 가 관찰된다면 Ranked ANCOVA 를 실시한다. 범주형 자료의 경우 군간 비교를 위해 각 군의 빈도수 및 비율을 구하고, Chi-square test 또는 Fisher's exact test(기대빈도가 5 이하인 경우)를 이용하여 분석한다.통계분석은 R-software(최신버전) 혹은 R 을 이용하는 jamovi software(최신버전)를 이용한다.

14.2 인구학적 기초자료

인구학적 조사, FSS 점수 및 증상에 대한 0-100 VAS 점수 등 기초조사 자료에 관하여 두 군간 통계학적 차이가 있는지 검정하기 위해 연속형 자료는 평균, 표준편차, 중앙값, 최소값 및 최대값 등을 구하고, independent two sample t-test 또는 Wilcoxon rank sum test 로 두 군간 비교를 실시한다. 범주형 자료의 경우 군간 비교를 위해 각 군의 빈도수 및 비율을 구하고, Chi-square test 또는 Fisher's exact test(기대빈도가 5 이하인 경우)를 이용하여 분석한다. 두 군간의 비교가능성이 확보되었는지 확인하기 위해서 FSS 점수, ChFS 점수 및 subscale 점수, EQ-5D-5L 점수, PSQI-K 의 Global 점수, K-MOCA 점수, BDI 점수, DF 점수, DB 점수, DF-DB 점수, K-BNT-15 점수, 전산화 신경인지기능 검사 점수, SPPB 검사 점수 등에 대하여 평균, 표준편차, 중앙값, 최소값 및 최대값 등을 구하고, independent two sample t-test 또는 Wilcoxon rank sum test 로 두 군간 비교를 실시한다.

14.3 유효성 평가변수에 대한 분석

14.3.1 일차 평가변수

12 주 중재의 복용 후 FSS 점수

14.3.2 이차 평가변수

1) 최종 복약 순응도
2) 최종 복약 순응도의 군별 차이
3) 방문 2, 3 시점에서의 FSS 점수
4) 방문 2, 3, 4 시점에서의 ChFS 점수 및 subscale 점수
5) 방문 4 시점에서의 EQ-5D-5L 점수
6) 방문 4 시점에서의 PSQI-K 의 Global score 점수
7) 방문 4 시점에서의 K-MOCA 의 총점수와 영역별 점수
8) 방문 4 시점에서의 BDI 점수
9) 방문 4 시점에서의 DF 점수, DB 점수, DF-DB 점수
10) 방문 4 시점에서의 전산화 신경인지기능 검사 점수
11) 방문 4 시점에서의 SPPB 검사의 보행속도 검사, 일어서기 검사, 정적균형 검사, 종합점수
12) Feasibility 평가를 위한 대상자 모집율, 중도탈락율, 중도탈락 이유 등에 대한 분석
13) 피로 증상에 대한 12 주 중재의 복용 후 치료군과 대조군 간의 치료성공률

14.4 안전성 변수에 대한 분석

14.4.1 이상반응

이상반응은 임상시험용의약품을 투여한 시험대상자에게 발생한 모든 유해하지 않은 증후, 증상 또는 질병으로 정의하며 각 중재군 별로 모든 이상반응과 약물이상반응(ADR), 중대한

이상반응(SAE), 중대한 약물이상반응(SADR)에 대한 발생환자 수, 발생률, 발현건수 및 발생률에 대한 95% 신뢰구간을 제시하고 투여군 간 차이는 Chi-square test 또는 Fisher's exact test 로 검정한다.
모든 이상반응은 MedDRA(Medical Dictionary for Regulatory Activities)의 신체기관(SOC) 및 선호용어(PT)로 코드화하여 군 별로 빈도, 비율 및 건수를 제시한다.

14.4.2 실험실 검사, 활력징후

활력징후 및 정량적인 실험실적 검사치, 심박변이도 검사는 한약제제군별로 투여 전/후 그리고 변화량에 대해 기술통계량을 제시하고 변화량에 대한 군간 차이는 independent two sample t-test 또는 Wilcoxon rank sum test, 각 군내 변화의 차이는 paired t-test 또는 Wilcoxon signed rank test 로 검정한다. 추가적으로, 실험실적 검사 결과는 한약제제 투여 전/후 정상(임상적으로 의미 없는 비정상 포함)/임상적으로 의미 있는 비정상 변화에 대한 분할표를 제시하고 각 군 내 변화는 McNemar's test(또는 McNemar's Exact test)로 검정한다.

15. 유효성 평가기준, 평가방법 및 해석방법

15.1 평가항목 및 평가방법

15.1.1 일차 평가변수

1) 12 주 중재의 복용 후 FSS 점수
FSS 점수를 12주 중재의 복용 후 군간 비교한다.

15.1.2 이차 평가변수

1) 최종 복약 순응도
: 최초 임상시험약의 투여 후 12주째(방문4 또는 종료시점) 전체 연구 대상자의 최종 복약 순응도(%)의 평균, 표준편차, 중앙값, 최소값 및 최대값을 구하여 제시한다.

2) 최종 복약 순응도의 군별 차이
: 최초 임상시험약의 투여 후 12주째 (방문4 또는 종료시점) 두 군의 최종 복약 순응도(%)의 평균, 표준편차, 중앙값, 최소값 및 최대값을 구하여 제시하고, 비교한다.

3) 방문 2, 3 시점에서의 FSS 점수
: FSS 점수를 각 시점에서 군간 비교한다.

4) 방문 2, 3, 4 시점에서의 ChFS 점수 및 subscale 점수
: 총 점수 및 physical(1-7번)/mental health(8-11번) sub-scale로 나누어 기록하고, 각 시점별 총 점수과 각 sub-scale 점수를 구하여 군간 비교한다.

5) 방문 2, 3, 4 시점에서의 EQ-5D-5L 점수

: EQ-5D-5L의 점수를 산출하고, 각 시점 별 점수를 군간 비교한다.

6) 방문 2, 3, 4 시점에서의 PSQI-K 의 Global score 점수
: 대상자 설문지 중 7종류의 component 점수를 구하고 이를 총 합산하여 구한 Global score의 총점을 산출하고, 각 시점 별 점수를 군간 비교한다.

7) 방문 4 시점에서의 K-MOCA 의 총점수와 영역별 점수
: 7개 영역의 점수를 구하고, 이를 총 합산하여 총점수를 산출하여, 군간 비교한다.

8) 방문 4 시점에서의 BDI 점수
: 4개 영역의 점수를 총 합산하여 총점수를 산출하여, 군간 비교한다.

9) 방문 4 시점에서의 DF 점수, DB 점수, DF-DB 점수
: 각 점수를 군간 비교한다.

10) 방문 4 시점에서의 전산화 신경인지기능 검사 점수
: 5 종 검사의 점수를 각각 군간 비교한다.

11) 방문 4 시점에서의 SPPB 검사의 보행속도 검사, 일어서기 검사, 정적균형 검사, 종합점수
: 3 종 검사의 점수와 종합점수를 군간 비교한다.

12) Feasibility 평가를 위한 대상자 모집율, 등록율, 중도탈락율, 중도탈락 이유 등에 대한 분석
: 대상자 모집율은 계획된 연구대상자 중 연구기간동안 모집한 대상자의 수로 계산한다. 대상자 등록율은 총 연구대상자를 총 스크리닝 대상자 수로 나누어 계산한다. 중도탈락율 및 중도탈락의 이유를 전체 연구대상자 및 각 처방군별로 계산하여 비교한다.

13) 최종 치료성공률
피로에 대한 임상적으로 유의미한 최소한의 차이(Minimal clinically important difference)에 관한 기존 자료에 근거하여, 임상시험약 투여전(방문1)과 투여 후(방문4)에서 평가한 피로증상 0-100 VAS의 차이가 15점이상인 경우 치료성공으로 정의하고, 두 군에서 치료성공한 대상자의 빈도수 및 비율을 제시한다[36].

16. 이상반응을 포함한 안전성 평가방법, 평가기준 및 해석방법

16.1 평가방법

이상반응(Adverse Event, AE)이란, 한약제제를 투여한 연구대상자에게 발생한 모든 유해하고 의도하지 않은 증후(sign, 실험실 실험 결과의 이상 등을 포함), 증상(symptom) 또는 질병을 말하며, 해당 한약제제와 반드시 인과관계를 가져야 하는 것은 아니다.

약물이상반응(Adverse Drug Reaction, ADR)이란 한약제제의 임의 용량에서 발생한 모든 유해하고 의도하지 않은 반응으로서 한약제제와의 인과관계를 부정할 수 없는 경우를 말한다. 본 연구에서는 인과관계가 '관련성 없음'으로 평가된 이상반응을 제외한 모든 이상반응은 약물이상반응으로 분류한다.

증례기록서에 이상반응의 증상 및 징후, 지속시간(시작 및 종료 날짜), 중증도, 한약제제와의 인과관계, 이상반응에 관련하여 취해진 조치, 결과 등에 대하여 빠짐없이 기록한다.

안전성 평가를 위해 실시되는 평가항목은 다음과 같다.
- 이상반응, 활력징후, 실험실 검사, 심박변이도 검사

16.2 평가기준

(1) 이상반응의 중증도

이상반응의 중증도(Intensity)는 다음과 같은 기준으로 평가 한다.

중증도(intensity)	설명
경증<br>(Mild)	대상자가 거의 느끼지 못할 정도로 정상적인 일상생활(기능)을 저해치 않는 정도, 특별한 처치가 필요 없음
중등증<br>(Moderate)	대상자가 불편감을 느낄 수 있으며, 정상적인 일상생활(기능)을 저해하는 정도, 대상자가 시험을 계속 할 수는 있으나 치료가 필요할 수도 있는 정도
중증<br>(Severe)	대상자가 매우 불편하여 일상생활(기능)이 불가능하고, 시험의 계속적인 참여가 불가능한 정도, 치료나 입원이 필요할 수 있는 정도

(2) 투여하는 한약제제와의 인과관계

한약제제와의 연관성 여부는 의약품 등의 안전에 관한 규칙 별지 제 77호에 준하여 연구자가 하기와 같이 분류하고, 연구자의 견해도 함께 기재한다.

인과관계	판단근거
관련성이 명백함	• 이 약을 투여하였다는 증거가 있고 이상반응 발현의 시간적 순서가 타당한 경우<br>• 이상반응이 다른 어떤 이유보다 의약품 투여에 의해 가장 개연성 있게 설명되는 경우<br>• 투여 중단으로 이상반응이 사라지는 경우<br>• 재 투여(re-challenge, 가능한 경우에만 실시) 결과가 양성인 경우<br>• 이상반응이 의약품 또는 동일 계열의 약물에 대해 이미 알려져 있는 정보와 일관된 양상을 보이는 경우
관련성이 많음	• 이 약을 투여하였다는 증거가 있고 이상반응 발현의 시간적 순서가 타당한 경우<br>• 이상반응이 다른 이유보다 의약품 투여에 의해 더욱 개연성 있게 설명되는 경우<br>• 투여 중단으로 이상반응이 사라지는 경우
관련성이 의심됨	• 이 약을 투여하였다는 증거가 있고 이상반응 발현의 시간적 순서가 타당한 경우<br>• 의약품의 투여가 다른 가능성이 있는 원인들과 같은 수준으로 이상반응에 기인한다고 판단되는 경우<br>• 투여중단으로(실시된 경우) 이상반응이 사라지는 경우
관련성이 적음	• 이 약을 투여하였다는 증거가 있는 경우<br>• 이상반응에 대해 보다 가능성이 있는 다른 원인이 있는 경우<br>• 투여 중단 결과(실시된 경우)가 음성이거나 모호한 경우<br>• 재 투여(re-challenge, 가능한 경우에만 실시) 결과가 음성이거나 모호한 경우
관련이 없음	• 시험대상자가 이 약을 투여 받지 않은 경우<br>• 약물투여와 이상반응 발현간의 시간적 순서가 타당하지 않은 경우<br>• 이상반응에 대해 다른 명백한 원인이 있는 경우
평가 불가능	• 이상반응에 대한 일부의 정보는 있으나 이 약과 관련성에 대한 평가를 할 수 없는 경우

(3) 이상반응 발현시 조치

이상반응 발현 시 투여 한약제제에 대하여 취해진 조치에 대해서 다음과 같이 분류한다.

- 투여중지(Drug withdrawn)

- 감량(Dose reduced)
- 용량변화 없었음(Dose not changed)
- 알 수 없음(Unknown)
- 해당사항 없음(Not applicable)

(4) 이상반응 결과

본 연구기간 중 발현된 이상반응의 결과를 다음과 같이 분류한다.

- 회복됨/해결됨(Recovered/Resolved)
- 회복중/해결중(Recovering/Resolving)
- 회복되지 않음/해결되지 않음(Not recovered/Not resolved)
- 회복되었으나 후유증이 남음/해결되었으나 후유증이 남음(Recovered with Sequelae/Resolved with Sequelae)
- 사망(Death)
- 알 수 없음(Unknown)

(5) 임상시험약의 예측가능한 이상반응

- 피부 : 발진, 두드러기, 습진, 피부염 등의 악화
- 소화기계 : 식욕부진, 위부불쾌감, 구역, 구토, 설사 등

(6) 주요 이상반응 관련 증상의 확인

- 피부 : 주관적 감각, 신체검진
- 소화기계 : 주관적 감각

16.3 이상반응 보고방법

연구책임자는 연구담당자 및 연구대상자 또는 보호자에게 임상시험약의 투여 후 나타날 수 있는 모든 이상반응에 대하여 교육을 실시하고 투여 후 나타나는 모든 현상에 대하여 보고하도록 교육을 실시한다.

임상시험약의 투여 후 전신적 또는 임상병리학적으로 나타나는 제반 증상에 대하여 종류, 발생기간, 정도, 처치, 치료약제, 경과, 인과관계 등에 대한 기록 및 보관은 임상시험 관리기준에 준하도록 연구대상자의 증례기록서에 기입한다.

16.3.1 중대한 이상반응·약물이상반응 (Serious AE·ADR)

중대한 이상반응·약물이상반응(Serious AE·ADR)이란 한약제제의 임의 용량에서 발생한 이상반응 또는 약물이상반응 중에서 다음의 어느 하나에 해당하는 경우를

말한다.

① 사망하거나 생명에 대한 위험이 발생한 경우
② 입원할 필요가 있거나 입원 기간을 연장할 필요가 있는 경우
③ 영구적이거나 중대한 장애 및 기능 저하를 가져온 경우
④ 태아에게 기형 또는 이상이 발생한 경우
⑤ 1)부터 4)까지의 사례 외에 약물 의존성이나 남용의 발생 또는 혈액질환 등 그밖에 의학적으로 중요한 상황이 발생하는 사례

또한, 위에서 열거한 상황이 아니더라도 기타 의학적으로 임상시험 대상자의 안위와 건강상태에 중대한 영향을 미칠 것으로 사료되는 상황이 발생한 경우, 시험자 및 관련 전문가의 의학적 판단에 따라 중대한 이상반응으로 간주할 것인가의 여부를 결정하고 이에 따라 적절한 조치를 취한다.

본 임상시험기간 중 새롭게 발생한 모든 중대한 이상반응에 대하여 연구책임자는 해당 연구기관의 규정에 따라 IRB에 보고한다. 연구책임자는 중대한 이상반응이 발생한 대상자의 상태를 지속적으로 관찰하고, 그 결과를 임상시험지원기관 및 IRB에 추가적으로 보고한다. 또한, 연구책임자는 해당 중대한 이상반응에 대하여 본 연구의 의뢰기관인 한국한의학연구원과 모니터링을 담당하는 모니터요원에게 인지 즉시 연락하여 적절한 조치가 취해지도록 한다.
사망 사례를 보고하는 경우 연구책임자는 임상시험지원기관과 심사위원회에 부검 소견서(부검을 실시한 경우만 해당한다)와 최종 의무기록(terminal medical reports) 등의 추가적인 정보를 제출하여야 한다.

아래 항목을 위한 ‘입원 또는 입원기간의 연장’인 경우 중대한 이상반응이 아닌 것으로 간주한다.

- 질병 진단을 위한 경우
- 성형수술을 위한 경우
- 정밀검진을 위한 경우
- 사소한 입원 (예, 파산 후 안정을 위한 입원, 의사가 필요하지 않다고 판단했음에도 불구하고 자발적인 입원)
- 장기 요양이나 재활을 위한 경우
- 간병인이 더 이상 재택간병을 할 수 없기 때문에 요양소나 재활시설에 입소하는 경우
- 연구와 관련된 유효성과 안전성 평가를 위한 경우
- 연구계획서의 대상질환의 계획된 치료를 위한 경우
- 환자 상태의 악화 없이 시험 전에 계획된 경우

16.3.2 예상하지 못한 약물이상반응(Unexpected Adverse Drug Reactions)

예상하지 못한 약물이상반응(Unexpected Adverse Drug Reactions)이란, 이용 가능한 의약품 관련 정보(예: 의약품의 첨부문서 등)에 비추어 약물이상반응의 양상이나 위해의 정도에서 차이가 나는 것을 말한다.

연구기간 중 대상자에게 "예상하지 못한 중대한 약물이상반응(Suspected Unexpected Serious Adverse Drug Reactions, SUSAR)"이 발생하였을 때에는 임상시험심사위원회(IRB)에 보고하여 시험의 지속 또는 중단 여부를 결정하고, 임상시험지원기관는 다음 각 항에서 정한 기간 내에 신속히 식품의약품안전처장에게 보고하도록 한다.

연구책임자는 연구를 시행함에 있어서 모든 제반 사항을 Declaration of Helsinki 에 준하여 시행한다.

16.4 이상반응의 추적관리

연구책임자는 모든 중대한 이상반응이 해결되거나 안정화 또는 추적관찰에 실패할 때까지 연구대상자를 추적 관찰하여야 한다. 연구대상자가 입원에서 퇴원하는 등 상태의 변화가 있는 경우, 중대한 이상반응 보고 이후 일정 기간 내에 진행 경과에 대한 보고서를 기록하여 대상자의 변화 양상을 살피고, 임상시험지원기관에게 이메일 또는 팩스로 제출하여야 한다.

17. 피해자 보상에 대한 규약

본 연구에 의한 연구대상자에게 신체적 손상(사망 포함)이 발생할 경우에는 연구임상시험지원기관이 법적인 책임을 지고 피해자 보상에 대한 규약에 의거하여 보상한다. 임상시험와 관련하여 발생한 이상반응의 교정처치 과정에서 발생한 손상의 경우에도 치료비를 지급한다.

18. 연구대상자 동의

연구자는 연구대상자가 연구에 참여하기 전 연구의 내용, 연구용 의약품의 효과, 이상반응 및 안전성에 대한 모든 사항을 충분히 설명하고 연구대상자의 자발적 동의서 서명을 득한 후 연구를 진행해야 한다.

19. 이상반응 발생 시 조치

이상반응 발생시 즉시 연구자로부터 필요 검사 및 치료를 받을 수 있도록 관리한다. 중대한 약물이상반응 발생시는 연구를 즉시 중지하고 이상반응 발생시 대응 원칙에 따라 신속하고 적절한 조치를 취한다.

이상반응이 발생한 연구대상자에 대해서는 이상반응이 해결되거나 안정화되거나, 추적관찰에 실패할 때까지 전화 또는 대상자의 외래방문으로 이상반응에 대해 계속 모니터링 하여 대상자의 안전을 확인하도록 하며 그 지속일을 증례기록서에 기록하여야 한다.

임상시험 중 “중대한 이상반응” 발생시 각 담당자의 의무는 다음과 같다.

1) 연구책임자의 의무

연구책임자는 연구 중 모든 중대한 이상반응이 발생한 경우 즉시(연구자자가 인지한 시간으로부터 24 시간 이내에) 연구지원기관 또는 모니터 요원에게 보고하고 기관의 임상시험심사위원회 규정에 따라 심사위원회에 보고한다. 추후에 상세한 내용이 포함된 추가보고 또한 동일한 시간 안에 보고 하여야 한다. 단, 예상하지 못한 중대한 약물이상반응의 경우 연구지원기관 및 임상시험심사위원회에 신속 보고 하여야 한다. 사망 사례를 보고할 경우 연구책임자는 연구지원기관 와 임상시험심사위원회에게 부검보고서(부검을 실시한 경우에 한함)와 최종 의무기록 등의 추가적인 정보를 제공하여야 한다.

2)연구담당자의 의무

연구담당자는 연구 실시 중에 중대한 이상반응 등이 발생한 경우에는 즉시 연구책임자 및 연구지원기관에게 보고하여야 한다.

3) 임상시험심사위원회의 의무

임상시험심사위원회는 예상하지 못한 중대한 약물이상반응이나 연구대상자의 안전성이나 임상시험의 실시에 부정적인 영향을 미칠 수 있는 새로운 정보에 관한 사항이 발생한 경우 연구의 일부 또는 전부에 대하여 중지명령 등 필요한 조치를 연구책임자에게 하여야 한다.

4) 연구지원기관의 의무

연구지원기관는 연구책임자 또는 연구담당자로부터 중대하고 예상하지 못한 모든 약물이상반응을 보고 받거나 알게 된 날로부터 15일 이내에 기타 관련된 연구자, 식품의약품안전처장 및 필요한 경우 심사위원회에 보고하여야 한다. 단, 사망을 초래하거나 생명을 위협하는 경우에는 연구지원기관이 이 사실을 보고 받거나 알게 된 날로부터 7일 이내에 보고하고, 이 경우에 상세한 정보를 최초 보고일로부터 8일 이내에 추가로 보고하여야 한다. 연구지원기관이 식품의약품안전처장에게 예상하지 못한 중대한

약물이상반응을 보고하려는 경우에는 약물이상반응 보고서에 CIOMS-Ⅰ서식 등 약물이상반응 요약서를 첨부하여 제출하여야 한다.

연구지원기관은 위의 보고와 관련하여 추가적인 정보가 있을 경우에는 안전성 정보를 주기적으로 해당 이상반응이 종결(해당 약물이상반응의 소실 또는 추적조사의 불가)될 때까지 보고하여야 한다. 이때 연구자는 보고에 대한 자료와 정보를 제공하는데 적극 협조하여야 한다.

20. 연구대상자의 안전 보호에 관한 대책

20.1 임상시험실시기관

실시기관의 장은 각 임상시험 단계별로 해당 임상시험의 실시에 필요한 임상시험실 및 설비와 전문인력을 갖추고, 해당 임상시험을 적정하게 실시할 수 있도록 준비에 완벽을 기해야 한다.

20.2 연구책임자

연구책임자는 연구기간 동안 해당 연구를 적절하고 안전하게 실시하기 위하여 필요한 인원 및 수량의 연구담당자와 장비 및 시설을 확보하여야 한다.

20.3 연구담당자

연구담당자는 연구계획서, 연구용 한약제제에 관한 정보, 연구와 관련된 의무 및 업무 등을 자세히 파악하고 연구에 임해야 한다.

20.4 연구계획서의 승인 및 수정

연구계획의 승인을 얻거나 승인 받은 연구를 변경하여 실시하고자 하는 경우, 연구 단계별로 계획서 또는 변경 계획서에 대하여 임상시험심사위원회(IRB)의 승인을 받는다.

20.5 연구계획서의 숙지

본 임상시험는 헬싱키 선언(Declaration of Helsinki)에 입각하여 연구대상자의 권리와 복지를 염두에 두고 준비된 것으로서, 연구책임자 및 담당자들은 임상시험계획을 정확히 분석 및 숙지하고, 연구대상자의 문제점을 적극적으로 대응하여야 한다.

20.6 정확한 연구 대상자의 선정

본 임상시험에 앞서 대상자와의 충분한 면담 및 검사를 통하여 대상자 적합여부에 대하여 철저히 평가한다.

20.7 이상반응 발생 시 조치

이상반응 발생시 즉시 연구자로부터 필요 검사 및 치료를 받을 수 있도록 관리한다. 중대한 약물이상반응 발생시는 시험을 중지하고 이상반응 발생시 조치사항에 따라 신속하고 적절한 조치를 취한다.

이상반응이 발생한 연구 대상자에 대해서는 이상반응이 해결되거나 안정화되거나, 추적관찰에 실패할 때까지 연구자의 전화 또는 대상자의 외래방문으로 이상반응에 대해 계속 모니터링 하여 대상자의 안전을 확인하도록 하며 그 지속일을 증례기록서에 기록하여야 한다.

21. 기타 임상시험를 안전하고 과학적으로 실시하기 위하여 필요한 사항

21.1 모니터링

연구대상자의 권리와 복지 보호, 보고된 임상시험 관련 자료가 근거문서와 대조하여 정확하고, 완전하며, 검증이 가능한지 여부 확인, 연구가 승인된 계획서, 의약품 임상시험 관리기준, 의약품 등의 안전에 관한 규칙 제 30 조 및 제 38 조 2 의 규정에 따라 수행되는지의 여부 확인을 위하여 모니터링을 실시한다.

연구에 대한 모니터링은 임상시험지원기관 또는 임상시험기관의 위임을 받은 모니터 요원의 정기적인 시험기관 방문과 전화를 통해서 이루어 질 것이다. 방문 시 모니터 요원은 기본적으로 연구대상자 기록 원본, 자료 보관(연구 파일) 등을 확인한다.

또한, 모니터 요원은 연구 진행과정을 잘 살피고, 문제가 있을 경우 연구책임자와 상의한다.

이들 방문의 적절한 시간은 연구자와 모니터 요원 간 협의하여 배분하여야 한다. 연구자는 또한 의약품 임상시험 관리기준에 정의된 것과 같이, 모니터 요원이 증례기록서에 기입된 자료들을 확인할 수 있는 대상자의 근거문서들(source documents: 병원 또는 개인 차트, 실험실 결과 기록, 예약 기록 등)을 열람할 수 있도록 해주어야 한다.

21.2 연구의 진행 점검

연구책임자는 임상시험지원기관에게 주기적으로 이상반응, 시험 진행 상황, 결과 등에 대하여 보고하며, 임상시험지원기관는 해당 임상시험에서 수집된 자료의 신뢰성을 확보하기 위하여

해당 연구계획서, 임상시험지원기관 표준작업지침서 및 관련규정 등에 따라 연구가 수행되는지 확인이 필요한 경우 점검을 실시할 수 있다.

21.3 자료의 보관

연구책임자는 연구관련 기본문서를 유지하고 제공할 의무가 있다. 기본문서란 연구의 수행과 그로부터 얻어진 자료의 질에 대하여 개별적 또는 전체적으로 평가가 가능하도록 해주는 문서를 말한다. 연구의 기본문서에는 모든 작업일지, 근거문서, 모니터링 기록과 약속 일정, 임상시험지원기관와 시험자간의 서신교환과 규정문서 등이 포함된다(예: 연구계획서와 그 개정판, 임상시험심사위원회 관련 서신, 허가/서명된 연구대상자 동의서, 증례기록서, 임상시험용 의약품수령증, 수불기록, 실험실검사 결과 등). 근거문서는 모든 관찰기록과 임상활동에 관한 기록, 임상시험의 평가와 재구성에 필요한 모든 보고서와 기록을 포함한다. 따라서 근거문서에는 연구계획서에 근거하여 실시된 모든 처치에 대한 기록 또는 이와 유사한 기록이 모두 포함된다. 관찰기록은 가능한 한 그 원본을 근거문서로서 보관해야 한다.

근거문서 및 기타 연구관련 문서는 항상 연구기관에 보관되어 있어야 하며, 연구자는 연구와 관련한 모든 기록을 결과보고서 작성 완료될 때까지 보관하고, 이 후 연구기관의 보관 책임자에게 이관한다. 연구실시 기관장 및 임상시험지원기관는 연구와 관련한 연구계획서, 서류, 승인서 및 다른 모든 자료를 본 임상시험의 완료일로부터 3 년간 보존하도록 한다. 보관 기간이 경과한 기록과 문서는 기밀 유지를 위하여 문서의 내용이 유출되지 않도록 파쇄할 것이며, 개인정보 관련 문서는 개인정보보호법 시행령 16 조에 따라 파기한다.

21.4 연구대상자의 비밀유지

연구대상자의 신원을 파악할 수 있는 기록은 비밀로 보장될 것이며, 연구 결과가 출판될 경우에도 대상자의 신원을 비밀상태로 유지한다. 구체적인 내용은 다음과 같다.

본 연구에 관련된 임상시험지원기관, 모니터 요원 및 점검자는 본 시험의 모니터링과 점검 및 진행사항 관리를 위한 목적으로 연구대상자의 기록을 열람할 수 있다. 연구자는 본 계획서에 서명함으로써, 국내의 법규와 윤리적 측면에서 임상시험지원기관 또는 임상시험수탁기관의 모니터 요원 및 점검자가 연구대상자의 차트와 증례기록서 기록을 검증하기 위하여 해당 문서를 검토하거나 복사할 수도 있음을 인정한다. 이러한 정보들은 기밀로 보관되어야 하며, 기밀 보관을 위한 시설과 그 관리기준을 갖추고 있어야 한다. 한편, 증례기록서 등 임상시험에 관련된 모든 서류에는 연구대상자 이름이 아닌 식별코드로 기록하고 구분한다.

21.5 인체유래물정보보호에 대한 대책

작성 필요

- 혈액 샘플 기증자의 개인정보 보호 및 연구 자료의 안전취급 대책: 인체유래물의 검사와 관련하여 임상연구 참가자의 개인정보 보호를 위해, 검체에는 이니셜, 채취 날짜, 검체의 종류(blood sample)가 명시되어 있는 label 을 부착하여 관리할 예정이다. 모든 자료는 임의번호를 부여하여 관리하고, 개인 신상정보에 대한 정보의 외부 유출이 없도록 관리할 계획이다.

- 혈액 샘플의 폐기: 인체유래물 연구 동의서에 기술되어 있는 대로 인체유래물을 기증자의 동의를 얻어 분석 한 후「폐기물관리법」제 13 조에 따른 기준 및 방법에 따라 즉시 폐기되며, 해당 기관의 휴업・폐업 등 해당 연구가 비정상적으로 종료될 때에는 법에서 정한 절차에 따라 인체유래물 등을 이관할 예정이다.

22. 임상시험 참여에 대한 혜택

본 임상시험 과정에서 소요되는 제반 비용(접수, 검사 및 처치 비용)은 연구자가 부담하며, 연구에 참여하는 대상자에게는 교통비 및 보상비 (총 350,000원(총 5회, 100,000원X2회 + 50,000원X3회))는 스크리닝 방문을 포함하여 각 방문 시 혈액을 채혈하는 경우 100,000원(Visit4), 그 외의 방문(Visit1,2,3)시 50,000원이 지급된다. 단, 본 임상시험과 관계없는 진료비는 대상자 본인이 부담해야 한다. 대상자에게 제공되는 교통비 및 보상비는 참여자별로 각 방문이 완료된 이후 회당 지급되며, 중도탈락, 대상자의 동의 철회 등으로 인한 임상시험 참여 중단시 중단 시점 이후의 교통비 및 보상비는 지급되지 않는다. 교통비 및 보상비는 계좌이체를 통해 지급될 것이며, 이에 각 방문이 완료된 이후 입금이 완료될 때까지 일정 기간이 소요될 수 있음을 미리 알리고 양해를 구한다. 위약군에 배정된 대상자는 모든 임상시험이 종료된 이후에 12주 분량의 경옥고를 제공 한다.

23. 임상시험용 검체의 이송, 보관, 분석, 폐기

본 임상시험을 위해 채취된 연구대상자의 검체에 대한 검사의 결과는 본 연구 목적에 한한다. 대상자의 검체는 검사 후 장기 보관되지 않을 것이며, 임상시험 종료 후 분석 기관의 SOP에 따라 폐기될 것이다. 대상자의 검체나 그 결과를 다른 목적을 위해 독립적인 제 3자에게 판매, 대여 또는 공여하지 않을 것이다.

24. 보고서 제출 및 출판에 관한 방침

연구책임자는 가능한 관련된 과학적인 정보의 자유로운 교류를 위해 노력해야 한다. 연구 결과의 발표나 출판은 총괄 연구책임자의 계획과 일치해야 한다. 본 임상시험의 결과보고서는

모든 연구대상자가 모든 방문을 완료하는 시점을 기준으로 작성될 것이다. 단, 지원기관 등의 요구에 따라 연구 도중 결과보고서를 작성할 수 있다.

25. 취약한 연구대상자에 대한 보호대책

취약한 연구대상자란 「의약품 등의 안전에 관한 규칙」 별표 4 <의약품 임상시험 관리기준> 제2호 더목에 따라 임상시험 참여와 관련한 이익에 대한 기대 또는 참여를 거부하는 경우 조직 위계상 상급자로부터 받게 될 불이익에 대한 우려가 자발적인 참여 결정에 영향을 줄 가능성이 있는 대상자 (의과대학, 한의과대학, 약학대학, 치과대학, 간호대학의 학생, 의료기관, 연구소의 근무자, 제약회사의 직원, 군인 등), 불치병에 걸린 사람, 집단시설에 수용되어 있는 사람, 실업자, 빈곤자, 응급상황에 처한 환자, 소수 인종, 부랑인, 노숙자, 난민, 미성년자 및 자유의사에 따른 동의를 할 수 없는 대상자를 말한다. 본 임상시험에서는 의료기관 및 연구소의 근무자, 실업자, 빈곤자, 65세 이상의 고령자의 참여가 가능하며, 연구자는 연구 대상자로 이들을 모집하기 위한 유인 행위를 하지 않을 것이며, 대상자의 자발적 참여 의지가 있는 경우에 한하여 그들의 권리와 복리를 보호하는 범위 내에서 참여를 허용할 것이다. 또한 자발적 의사를 재확인하기 위하여 동의서에 '본인은 본 임상시험에 관한 설명을 듣고 자발적으로 참여하였습니다.'라고 명시한 뒤 서명을 실시할 것이다.

26. 연구대상자 모집방안

연구대상자는 실시기관 내외에 별도의 모집공고문을 부착하고, 홈페이지 등의 온라인 모집공고문을 게시하여 대상자를 모집할 계획이다. 모집공고문에는 연구담당자 또는 연구 코디네이터 등의 연락처를 기재하고 대상자가 전화를 통해 참여 의사를 표현할 시 일정을 약속하여 서면 동의 실시 후 스크리닝 평가를 실시할 계획이다.

27. 위험/이익 평가

연구의 잠재적 위험: 약물투여와 관련하여 위알도스테론증, 근병증, 간기능장애, 간질성폐렴, 피부의 발진, 식욕부진 등의 소화기계 문제 등이 드물게 발생할 수 있다.

연구의 잠재적 이익: 연구대상자의 증상을 개선시키는데 도움이 될 것으로 사료된다.

위험/이익 분석 결과: 위험 및 잠재적 위험과 잠재적 이익을 고려하였을 때 이익 대비 위해가 더 크게 상회하지 않을 것으로 판단되며, 이에 따라 임상시험으로서 가치가 있을 것으로 사료된다.

28. 임상시험의 윤리적 측면

28.1 임상시험 심사위원회

임상시험 심사위원회는 연구계획서 및 본 임상시험 수행을 위한 모든 관련 문서들을 검토하여 헬싱키선언에 근거한 윤리적 원칙이 충분히 반영되었는지 확인하여야 한다. 또한, 대상자의 권리·안전·복지를 보호하고, 취약한 환경에 있는 연구대상자가 연구에 참여한 경우 해당 대상자의 참여가 타당한지 검토하여야 한다.

28.2 연구책임자

연구책임자의 책임 하에 본인이 소속된 임상시험 실시기관에서 임상시험를 실시하도록 한다. 연구책임자는 임상시험계획서와 헬싱키 선언에 따른 윤리적 원칙, ICH의 GCP 규정 (ICH Topic E6) 또는 의약품 임상시험 관리기준 등 관련법규에 따라 임상시험이 수행되도록 한다. 특히 임상시험 참여에 자발적으로 참여 동의한 연구대상자 만을 임상시험에 포함시켜야 한다.

참고문헌

1. Chopra, V., et al., *Sixty-Day Outcomes Among Patients Hospitalized With COVID-19.* Annals of Internal Medicine, 2020.
2. O'Mahoney, L.L., et al., *The prevalence and long-term health effects of Long Covid among hospitalised and non-hospitalised populations: A systematic review and meta-analysis.* EClinicalMedicine, 2023. **55**: p. 101762.
3. Townsend, L., et al., *Persistent fatigue following SARS-CoV-2 infection is common and independent of severity of initial infection.* Plos one, 2020. **15**(11): p. e0240784.
4. Clayton, E.W., *Beyond myalgic encephalomyelitis/chronic fatigue syndrome: an IOM report on redefining an illness.* Jama, 2015. **313**(11): p. 1101-1102.
5. Shin, C.M., et al., *DA-9701 on gastric motility in patients with Parkinson's disease: a randomized controlled trial.* Parkinsonism & related disorders, 2018. **54**: p. 84-89.
6. Soriano, J.B., et al., *A clinical case definition of post-COVID-19 condition by a Delphi consensus.* The Lancet Infectious Diseases, 2022. **22**(4): p. e102-e107.
7. *COVID-19 rapid guideline: managing the long-term effects of COVID-19. NICE. '2022.1.3.*
8. Kim, Y., et al., *Preliminary guidelines for the clinical evaluation and management of long COVID.* Infection & chemotherapy, 2022. **54**(3): p. 566-597.
9. Islam, M.F., J. Cotler, and L.A. Jason, *Post-viral fatigue and COVID-19: lessons from past epidemics.* Fatigue: Biomedicine, Health & Behavior, 2020. **8**(2): p. 61-69.
10. 남동현, *만성피로증후군에 대한 보중익기탕과 그 변방의 효과: 체계적인 문헌고찰.* 대한한의학회지, 2020. **41**(1): p. 93-106.
11. 김지원, et al., *만성피로의 한의임상진료현황에 대한 조사 연구.* 동의생리병리학회지, 2018. **32**(2): p. 126-133.
12. 김용안, et al., *경옥고의 항피로 효능.* 생약학회지, 2016. **47**(3): p. 258-263.
13. 주희철, *경옥고 투여가 유산소 운동시 혈중 피로회복에 미치는 영향.* 중앙대학교 대학원 체육학과 박사학위 논문, 2004.
14. 김동건, 박원형, and 차윤엽, *경옥고 섭취가 고등학교 축구선수의 운동수행능력 향상 및 피로 회복에 미치는 영향.* 동의생리병리학회지, 2011. **25**(5): p. 934-944.
15. Jang, S., et al., *Telemedicine and the use of Korean medicine for patients with COVID-19 in South Korea: observational study.* JMIR Public Health and Surveillance, 2021. **7**(1): p. e20236.
16. Song, H.-S., et al., *Anti-inflammatory activity of Kyungok-go on Lipopolysaccharide-Stimulated BV-2 Microglia Cells.* Journal of Korean Medicine, 2022. **43**(4): p. 20-32.

17. Kim, J.-W., et al., *The efficacy, effectiveness, and safety of Kyung-ok-ko: A narrative review.* Medicine, 2022. **101**(45): p. e31311.
18. Pinto, C., et al., *Automated Mechanical Peripheral Stimulation Improves Gait Parameters in Subjects With Parkinson Disease and Freezing of Gait: a Randomized Clinical Trial.* American journal of physical medicine & rehabilitation, 2018. **97**(6): p. 383-389.
19. Zhang, J., Y.Z. Ma, and X.M. Shen, *Evaluation on the efficacy and safety of Chinese herbal medication Xifeng Dingchan Pill in treating Parkinson's disease: study protocol of a multicenter, open-label, randomized active-controlled trial.* Journal of integrative medicine, 2013. **11**(4): p. 285-290.
20. Bültmann, U., et al., *Measurement of prolonged fatigue in the working population: determination of a cutoff point for the checklist individual strength.* Journal of occupational health psychology, 2000. **5**(4): p. 411.
21. Fukuda, K., et al., *The chronic fatigue syndrome: a comprehensive approach to its definition and study.* Annals of internal medicine, 1994. **121**(12): p. 953-959.
22. Sharpe, M., *A report–chronic fatigue syndrome: guidelines for research.* Journal of the Royal Society of Medicine, 1991. **84**(2): p. 118-121.
23. 대한감염학회, *만성 코로나 19증후군(Long COVID) 진료지침 예비 권고안.* 2022.
24. Naik, H., et al., *Evaluating fatigue in patients recovering from COVID-19: validation of the fatigue severity scale and single item screening questions.* Health and Quality of Life Outcomes, 2022. **20**(1): p. 1-9.
25. Ha, H., et al., *Cross-Cultural Validation of the Korean Version of the Chalder Fatigue Scale.* International Journal of Behavioral Medicine, 2018. **25**(3): p. 351-361.
26. *울산대학교 산학협력단, 건강관련 삶의 질 측정도구(EQ-5D)의 타당도 평가, 질병관리본부.*
27. Herdman, M., et al., *Development and preliminary testing of the new five-level version of EQ-5D (EQ-5D-5L).* Quality of life research, 2011. **20**(10): p. 1727-1736.
28. Buysse, D.J., et al., *The Pittsburgh Sleep Quality Index: a new instrument for psychiatric practice and research.* Psychiatry research, 1989. **28**(2): p. 193-213.
29. Teasdale, H., et al., *Safety and efficacy of high definition tDCS for proprioception and balance in Parkinson's disease: a pilot randomised trial.* Movement disorders, 2018. **33**: p. S516-.
30. Seo, J., H. Lee, and M.K. Sunwoo, *Validation of MoCA–MMSE conversion scales in Korean Patients with Cognitive Impairments (4013)*. 2020, AAN Enterprises.
31. Teasdale, H.E., et al., *Abstract #31: safety and efficacy of high definition tDCS for proprioception and balance in Parkinson's disease.* Brain stimulation, 2019. **12**(2): p. e11-e12.
32. 이영호 and 송종용, *BDI, SDS, MMPI-D 척도의 신뢰도 및 타당도에 대한 연구.* Korean Journal of Clinical Psychology, 1991. **10**(1): p. 98-113.

33. Calabria, M., et al., *Post-COVID-19 fatigue: the contribution of cognitive and neuropsychiatric symptoms.* Journal of neurology, 2022. **269**(8): p. 3990-3999.
34. 구신실 and 박재국, *뇌성마비아동에 대한 전산화신경인지기능검사 (CNT) 의 유용성 연구.* 지체중복건강장애연구 (구 중복· 지체부자유아교육), 2010. **53**(2): p. 137-155.
35. Jung, H.W., et al., *Validation of a multi–sensor-based kiosk for Short Physical Performance Battery.* Journal of the American Geriatrics Society, 2019. **67**(12): p. 2605-2609.
36. Nordin, Å., et al., *Minimal important differences for fatigue patient reported outcome measures—a systematic review.* BMC Medical Research Methodology, 2016. **16**(1): p. 1-16.
